# Hydrosilane σ‐Adduct Intermediates in an Adaptive Zinc‐Catalyzed Cross‐dehydrocoupling of Si−H and O−H Bonds

**DOI:** 10.1002/chem.202101146

**Published:** 2021-05-21

**Authors:** Smita Patnaik, Uddhav Kanbur, Arkady Ellern, Aaron D. Sadow

**Affiliations:** ^1^ Department of Chemistry Iowa State University Ames IA 50011 USA; ^2^ US Department of Energy Ames Laboratory Iowa State University Ames IA 50011 USA

**Keywords:** dehydrogenative cross-coupling, saturation kinetics, sigma-adducts, silyl ethers, zinc catalysis

## Abstract

Three‐coordinate ^Ph^BOX${}^{\mathrm{Me}{}_{2}}$ ZnR (^Ph^BOX${}^{\mathrm{Me}{}_{2}}$ =phenyl‐(4,4‐dimethyl‐oxazolinato; R=Me: **2 a**, Et: **2 b**) catalyzes the dehydrocoupling of primary or secondary silanes and alcohols to give silyl ethers and hydrogen, with high turnover numbers (TON; up to 10^7^) under solvent‐free conditions. Primary and secondary silanes react with small, medium, and large alcohols to give various degrees of substitution, from mono‐ to tri‐alkoxylation, whereas tri‐substituted silanes do not react with MeOH under these conditions. The effect of coordinative unsaturation on the behavior of the Zn catalyst is revealed through a dramatic variation of both rate law and experimental rate constants, which depend on the concentrations of both the alcohol and hydrosilane reactants. That is, the catalyst adapts its mechanism to access the most facile and efficient conversion. In particular, either alcohol or hydrosilane binds to the open coordination site on the ^Ph^BOX${}^{\mathrm{Me}{}_{2}}$ ZnOR catalyst to form a ^Ph^BOX${}^{\mathrm{Me}{}_{2}}$ ZnOR(HOR) complex under one set of conditions or an unprecedented σ‐adduct ^Ph^BOX${}^{\mathrm{Me}{}_{2}}$ ZnOR(H−SiR′_3_) under other conditions. Saturation kinetics provide evidence for the latter species, in support of the hypothesis that σ‐bond metathesis reactions involving four‐centered electrocyclic 2σ–2σ transition states are preceded by σ‐adducts.

## Graphical abstract

**σ‐Silane adducts and alcohol adducts are kinetically detected intermediates** in zinc‐catalyzed σ‐bond metathesis‐type dehydrocoupling reactions. These intermediates feature prominently in the reactivity of coordinatively unsaturated zinc centers, which efficiently mediate Si−O bond formation. The catalyst is responsive to reaction conditions, adapting as a function of concentration to follow the kinetically preferred mechanism.

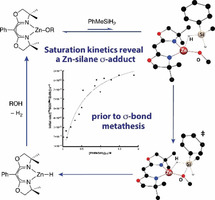

## Introduction

Silicon‐oxygen bond formation has wide‐ranging impact in synthetic applications, ranging from the construction of organic‐inorganic hybrid materials[^1^, ^2^, ^3^] to the assembly of complex molecules.^[4]^ Silyl ethers themselves have important roles in cross‐coupling,^[5]^ as templates for cyclization,^[6]^ as protecting groups,[^7^, ^8^] and even improving the efficacy of medicinal compounds.[^9^, ^10^] These moieties are conventionally formed from alcohols and chlorosilanes; this approach, however, is hindered by the formation of HCl or salts as by‐products, moisture sensitivity and competing hydrolysis of chlorosilanes, as well as the limited reactivity of bulky tertiary alcohols and bulky chlorosilanes,^[11]^ incompatibilities with base‐sensitive groups,[^12^, ^13^] and difficulties selecting for a desired stoichiometry needed to assemble multiple components into synthesis‐enabling scaffolds. Related reactions of alcohols with labile silyl ethers or silazanes also involve hydrolytically sensitive reactants.[^14^, ^15^]

Alternatively, catalytic dehydrogenative cross‐coupling of hydrosilanes and alcohols can provide partly substituted products by influencing reaction rates, the H_2_ by‐product is inert, and alkyl‐ and arylsilanes can be stored in air prior to dehydrocoupling. Late‐transition‐metal complexes based on Re,^[16]^ Rh,[^17^, ^18^] Ni,^[19]^ and Ir^[20]^ are catalysts for these cross‐dehydrocoupling reactions; however, in some cases these systems also mediate isomerization or hydrosilylation of C=C or C=O moieties. Basic catalysts such as sodium hydroxide overcome this limitation but are restricted to secondary and tertiary silanes,[^21^, ^22^] as are B(C_6_F_5_)_3_‐catalyzed reactions.^[23]^

Hydridozinc species also catalyze these cross‐dehydrocouplings;[^24^, ^25^, ^26^, ^27^, ^28^, ^29^] however, a divergent picture of the fundamental nature of hydridozinc catalysts has emerged, obscuring design principles. In particular, catalytic product formation is observed with coordinatively saturated (ZnX_2_L_2_, 8‐electron) hydride and super‐saturated (ZnX_2_L_3_, 10‐electron) alkoxide pre‐catalysts, as well as with dimeric hydride‐bridged *N*‐heterocyclic carbene‐coordinated zinc pre‐catalysts^[24]^ which likely access lower coordinate catalytic sites (ZnX_2_L, 6‐electron). For example, the interconverting four‐coordinate [*κ* ^3^‐Tptm]ZnH and five‐coordinate [*κ* ^4^‐Tptm]ZnOR′ (Tptm=tris(2‐pyridylthio)methyl) catalyzes the methanolysis of phenylsilane with high turnover number (TON) of 10^5^ and turnover frequency of 10^6^ h^−1^.^[26]^ These pre‐catalysts also mediate carbonyl hydrosilylation, which involves related Si−O bond formations. In hydrosilylations, however, TON and rates do not necessarily benefit from coordinatively unsaturated zinc pre‐catalysts.^[30]^ Such behavior suggests that complex reaction pathways underpin deceptively simple transformations.

**Scheme 1:**
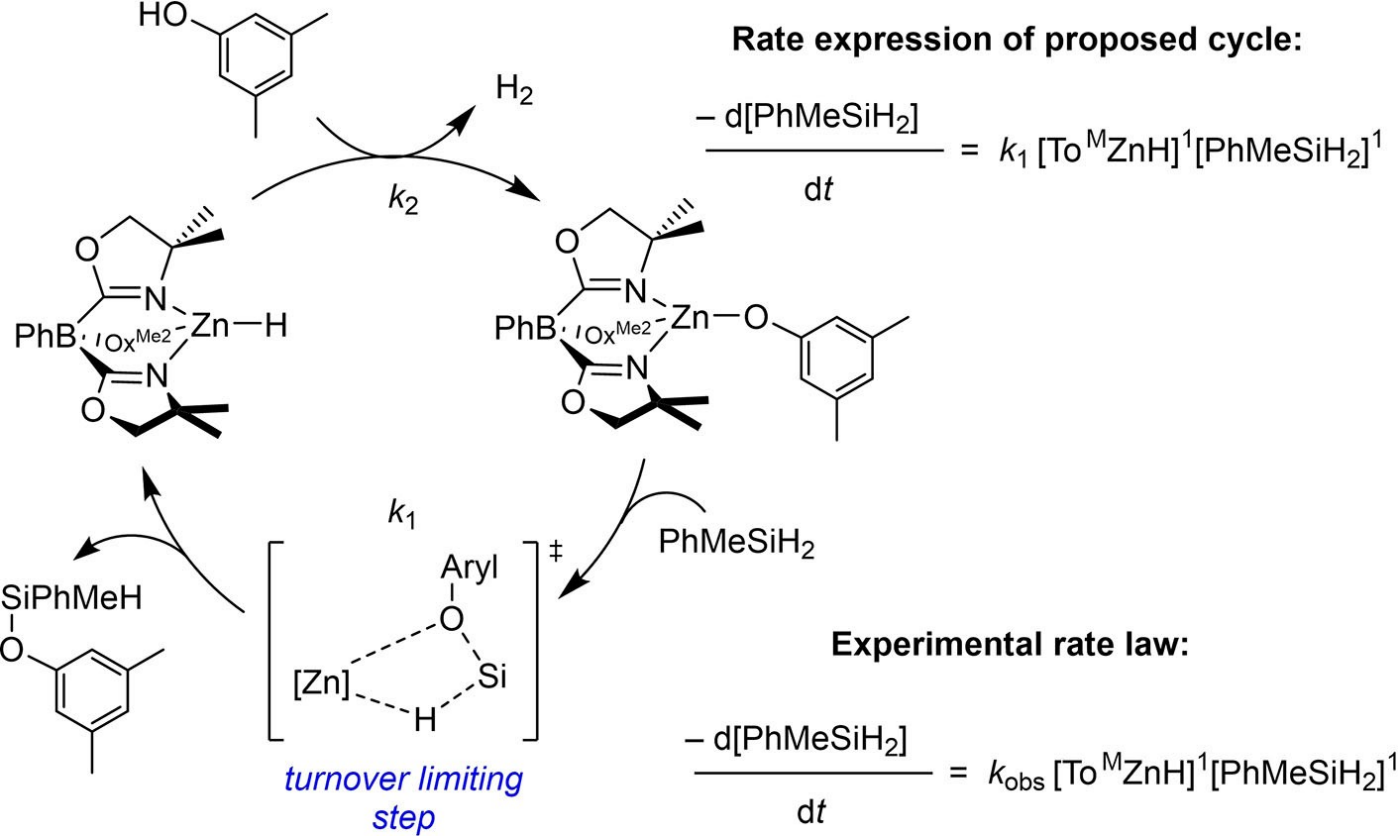
Proposed cycle for To^M^Zn‐catalyzed cross‐dehydrocoupling of hydrosilanes and alcohols.

A two‐step catalytic cross‐dehydrocoupling mechanism has been proposed based on kinetic studies of conversions catalyzed by the four‐coordinate To^M^ZnH (To^M^=tris(4,4‐dimethyl‐2‐oxazolinyl)phenylborate),^[25]^ structurally and spectroscopically characterized hydridozinc and alkoxyzinc intermediates, and kinetically well‐defined elementary steps (Scheme 1). The key turnover‐limiting silicon‐oxygen bond formation is proposed to occur by σ‐bond metathesis,^[31]^ involving cleavage of Zn−OR and Si−H bonds and formation of Zn−H and Si−O bonds via a four‐centered, concerted, electrocyclic transition state. Related steps are generally accepted for C−H and Si−H bond activations by d^0^ early transition metal and rare earth organometallic compounds,[^32^, ^33^, ^34^] and these elementary steps are ubiquitous in catalytic transformations including alkane hydrogenolysis,[^35^, ^36^] silane polymerization,^[37]^ hydrosilylation,[^38^, ^39^, ^40^, ^41^] alkane silylation,^[42]^ and hydromethylation of alkenes.^[43]^ Theoretical results suggest that Si−H or C−H σ‐coordination precedes the four‐centered transition state,[^44^, ^45^, ^46^] although experimental evidence for σ‐complexes as intermediates is limited to kinetic isotope effects (KIEs).

KIEs in σ‐bond metathesis‐type E−E′ bond formations vary considerably, with Si−N and Si−C bond forming steps characterized by *k*_H_/*k*_D_=1,[^47^, ^48^] whereas the *k*_H_/*k*_D_ for Si−Si and P−P bond forming steps are typically ∼3.[^37^, ^49^] The latter, non‐unity KIEs likely result from the long M−E and E−E bonds in the cyclic transition state, involving a M−H−E angle that approaches linearity. In contrast, either transfer of H in a bent geometry or a rate‐determining step which does not break the E−H bond could lead to the KIE ∼1. For example, the nearly unitary primary *k*_H_/*k*_D_ in the reaction of Cp_2_HfHCl and the stannane Mes_2_SnH_2_ (Mes=2,4,6‐C_6_Me_3_H_2_) or Mes_2_SnD_2_, to form a Hf−Sn bond, was interpreted in terms of a σ‐coordinated intermediate prior to H−H bond formation.^[50]^ Also, the near unity isotope effect in reactions of To^M^MgNH*t*Bu and PhMeSiH_2_ or PhMeSiD_2_, along with a companion Hammett study, were instead interpreted as the resulting from an asynchronous σ‐bond metathesis sequence involving rate‐controlling N−Si bond formation prior to H migration to Mg via β‐H elimination‐like step.^[47]^

Noting that To^M^ZnH is an 8‐electron species, a strategy for increasing catalytic performance could involve electronically unsaturated, three‐coordinate zinc centers. To investigate this idea, we targeted zinc species supported by bidentate, monoanionic bis(4‐R‐oxazolinato) ligands (BOX),[^51^, ^52^, ^53^, ^54^] which are the LX analogues of common neutral bis(oxazoline) ligands.^[55]^ The C1‐phenyl ligand was chosen to impede undesired ancillary ligand redistribution reactions, observed for BOXZnOR and diketiminatozinc in the presence of alcohols,[^54^, ^56^] without hindering access to the active site. Here, we report the catalytic properties of ^Ph^BOX alkylzinc compounds in dehydrocoupling of alcohols and hydrosilanes. The straightforward syntheses of the alkylzinc pre‐catalysts, mild conditions, solvent‐free reactions and high TONs make this methodology attractive. Furthermore, detailed kinetic investigations reveal that multiple catalytic pathways become accessible under varying conditions, as a consequence of three‐coordinate electronically unsaturated zinc pre‐catalysts.

## Results and Discussion

### Pre‐catalyst synthesis and characterization

**Figure 1:**
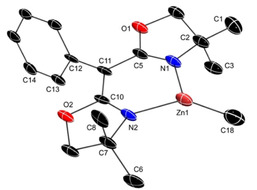
Thermal ellipsoid plot of ^Ph^BOX${}^{\mathrm{Me}{}_{2}}$ ZnMe (**2 a**) at 50 % probability. H atoms are excluded for clarity. Selected interatomic distances [Å]: Zn1−C18, 1.93(1); Zn1−N1, 1.947(9); Zn1−N2, 1.947(9); N1−C5, 1.31(2); N2−C10, 1.31(2); C5−C11, 1.44(2); C10−C11, 1.39(2).

**Figure 2:**
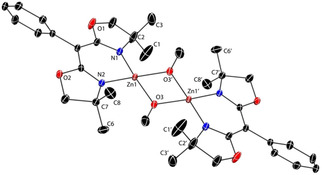
Thermal ellipsoid plot of {^Ph^BOX${}^{\mathrm{Me}{}_{2}}$ Zn(μ‐OMe)}_2_ (**3 a**)_2_ at 50 % probability. H atoms are not included in the plot. Selected interatomic distances [Å]: Zn1−O3, 1.976(2); Zn1−N1, 1.973(2); Zn1−N2, 1.989(3).

Reactions of ^Ph,H^BOX${}^{\mathrm{Me}{}_{2}}$ and dimethylzinc or diethylzinc provide the heteroleptic compounds ^Ph^BOX${}^{\mathrm{Me}{}_{2}}$ ZnMe (**2 a**) and ^Ph^BOX${}^{\mathrm{Me}{}_{2}}$ ZnEt (**2 b**) in high yield after 12 h at room temperature [90–92 %; Eq. (1)]. Singlets assigned to methyl and methylene groups on the oxazoline in the ^1^H and ^13^C{^1^H} NMR spectra of **2 a** and **2 b** were consistent with *C*_2v_ symmetric species. The oxazoline methyl resonance correlated to a single ^15^N NMR resonance at around −215 ppm in ^1^H,^15^N HMBC experiments, much closer to the more shielded chemical shift for the iminoenamine tautomer than to that of the diimine form of the proligand. The IR spectra showed a band, assigned to the carboximidate moiety, at lower frequency (*ν*_CN_=1609 cm^−1^) than in the free proligand (*ν*_CN=_1648 cm^−1^). X‐ray quality crystals of **2 a** (Figure 1) and **2 b** (see the Supporting Information) were obtained from pentane solutions cooled at −30 °C.
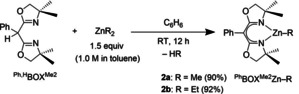



Single‐crystal X‐ray diffraction studies reveal that the approximately *C*_2_‐symmetric alkyl zinc compounds **2 a** (Figure 1) and **2 b** are three‐coordinate, with Zn1, two oxazoline C=N and the backbone C11 as vertices in a nearly planar six‐membered ring. All torsion angles of this planar and symmetrical chelate ring are less than 10°, with equivalent distances for Zn−N pairs, N=C pairs, and C−N pairs. The C1 and C6 dimethyl substituents of the oxazoline rings are pseudoequatorial, whereas C3 and C8 are pseudo‐axial in **2 a**, as defined by transannular torsion angles ∠C1−C2−C7−C6 (−70(1)°) and ∠C3−C2−C7−C8 (165.0(9)°).

**Scheme 2:**
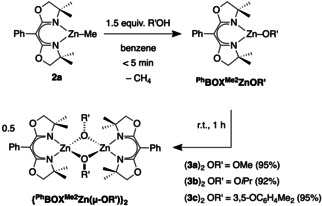
Synthesis and dimerization/precipitation of ^Ph^BOX${}^{\mathrm{Me}{}_{2}}$ ZnOR′.

Reactions of **2 a** with methanol, isopropanol, or 3,5‐dimethylphenol provide ^Ph^BOX${}^{\mathrm{Me}{}_{2}}$ ZnOR′ compounds (R′=Me **3 a**, *i*C_3_H_7_ **3 b**, C_6_Me_2_H_3_ **3 c**) in fewer than 5 mins in [D_6_]benzene or [D]chloroform, as determined by ^1^H NMR spectroscopy. The dimeric products (**3 a**–**c**)_2_ precipitate from the benzene reaction mixture over 1 h at room temperature and are easily isolated in excellent yield (Scheme 2). The qualitative trend in precipitation rate depends on the alcohol substituent (Me>*i*C_3_H_7_>C_6_Me_2_H_3_). The solids are insoluble in [D_6_]benzene, but dissolve in [D_2_]methylene chloride or [D]chloroform. In addition, the compounds may be generated and used in situ in [D_6_]benzene. ^1^H and ^13^C{^1^H} NMR spectra of isolated species, dissolved in [D]chloroform, consisted of singlet resonances assigned to oxazoline methyl and methylene groups. These spectra are identical to those obtained by in situ reaction of **2 a** and R′OH in [D]chloroform. 2D DOSY NMR measurements performed on a mixture of zinc ethyl **2 b** and zinc methoxy **3 a** in [D_6_]benzene revealed their similar diffusion constants (ca. 2.1×10^−8^ and 1.6×10^−8^ m^2^/s, respectively). Because the molecular weight of **2 b** differs by only 2 amu from that of the monomeric form of **3 a**, these results suggest that the soluble form of **3 a** is mostly monomeric.

Single‐crystal X‐ray diffraction studies of (**3 a**)_2_ and (**3 b**)_2_ reveal that the isolated dimeric species contain planar Zn_2_O_2_ cores composed of four‐coordinate zinc centers bridged by alkoxide groups (see Figure 2 for (**3 a**)_2_ and Supporting Information for (**3 b**)_2_). Both structures contain a crystallographic inversion center at the center of the Zn_2_O_2_ core, relating the two ^Ph^BOX${}^{\mathrm{Me}{}_{2}}$ Zn units and the two alkoxide ligands. The Zn−O distances (mean 1.975 Å) within both compounds are equivalent within 3σ error, giving rhombus‐shaped cores. In addition, the ^Ph^BOX${}^{\mathrm{Me}{}_{2}}$ Zn moieties form a similar planar six‐member chelate ring as in the zinc alkyls; however, the methyl substituents are more symmetrically disposed about the plane of the chelate ring compared to the twisted conformations in **2 a** and **2 b**. That is, the torsion angles in (**3 a**)_2_ ∠C1−C2−C7−C8 (115.7(4)°) and ∠C3−C2−C7−C6 (121.5(3)°) provide an approximate *C*_2v_ conformation. These different conformations likely reflect low energy barriers (flexibility) in the BOXZn motif, despite the rigid six‐member chelate ring.

### Catalytic alcohol/hydrosilane dehydrocoupling reactions

The monomeric species **2 a** is a pre‐catalyst for the dehydrocoupling of primary, secondary, or tertiary alcohols and primary or secondary organosilanes to give trialkoxy, dialkoxy, or monoalkoxy organosilanes and H_2_ as the by‐product [Eq. (2); Table 1]. Catalytic conversions occur readily at room temperature, although a few examples are improved upon mild heating. In addition, solvent‐free reactions provide products efficiently.
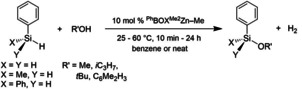



**Table 1:** **2 a**‐Catalyzed dehydrogenative cross‐coupling of hydrosilanes and alcohols.

Reactants	Products	Conditions^[a]^	Yield^[b]^ [%]
PhSiH_3_+1.1 MeOH	PhH_2_SiOMe+PhHSi(OMe)_2_	RT, <10 min	15 : 50
PhSiH_3_+3.5 MeOH	PhSi(OMe)_3_	RT, <10 min	100 (98)
PhSiH_3_+3.5 MeOH	PhSi(OMe)_3_	RT, neat, <10 min	100
PhSiH_3_+3.5 MeOH	PhSi(OMe)_3_	0.00001 mol % **2 a**, RT, neat, 1 h	100
PhSiH_3_+3.5 *i*PrOH	PhSi(O*i*Pr)_3_	60 °C, 4 h	95 (90)
PhSiH_3_+3.5 *i*PrOH	PhSi(O*i*Pr)_3_	RT, neat, 24 h	98
PhSiH_3_+1.1 *t*BuOH	PhH_2_SiO*t*Bu	RT, 24 h	88
PhSiH_3_+1.1 *t*BuOH	PhH_2_SiO*t*Bu	RT, neat, 24 h	100
PhMeSiH_2_+1.1 MeOH	PhMeHSiOMe+PhMeSi(OMe)_2_	RT, 1 h	42 : 30
PhMeSiH_2_+3.5 MeOH	PhMeSi(OMe)_2_	RT, <10 min	88
PhMeSiH_2_+3.5 MeOH	PhMeSi(OMe)_2_	RT, neat, 30 min	100 (92)
PhMeSiH_2_+1.1 *i*PrOH	PhMeHSiO*i*Pr	60 °C, 4 h	73 (68)
PhMeSiH_2_+1.1 *t*BuOH	PhMeHSiO*t*Bu	60 °C, 4 h	80
PhMeSiH_2_+1.1 *t*BuOH	PhMeHSiO*t*Bu	RT, neat, 24 h	100 (82)
PhMeSiH_2_+1.1 ArylOH Aryl=C_6_Me_2_H_3_	PhMeHSiOAryl	60 °C, 3 h	100 (65)
Ph_2_SiH_2_+3.5 MeOH	Ph_2_Si(OMe)_2_	RT, neat, 10 min	100 (94)
Ph_2_SiH_2_+1.1 *i*PrOH	Ph_2_HSiO*i*Pr	RT, 24 h	100
Ph_2_SiH_2_+3.5 *i*PrOH	Ph_2_HSiO*i*Pr	RT, 24 h	100 (89)
Ph_2_SiH_2_+1.1 *t*BuOH	Ph_2_HSiO*t*Bu	RT, 24 h	100 (85)

[a] Standard conditions unless specified: 10 mol % **2 a** pre‐catalyst, benzene (2 mL) or solvent‐free (neat), 0.9 mmol hydrosilane. Reactions performed with 3.15 mmol MeOH, 3.15 mmol *i*C_3_H_7_OH, 0.99 mmol *i*C_3_H_7_OH, 0.99 mmol *t*BuOH. [b] (Isolated yield).

Methanol gives quantitative substitution of all silicon hydrides with 10 mol % **2 a**, producing PhSi(OMe)_3_, PhMeSi(OMe)_2_, or Ph_2_Si(OMe)_2_ within 10 min at room temperature in benzene or under solvent‐free conditions. In addition, **2 a** provides a long‐lived and effective catalyst, resulting in up to 10^7^ turnovers (3×10^6^ equiv. of PhSi(OMe)_3_ are formed) with low catalyst loading. Likewise, the reaction of PhSiH_3_ and excess *i*C_3_H_7_OH provides PhSi(O*i*C_3_H_7_)_3_ after 1 day under neat conditions at room temperature or after 4 h in benzene at 60 °C.

Attempts to synthesize mono‐methoxy PhH_2_SiOMe by Zn‐catalyzed reactions of 1 equivalent of MeOH and PhSiH_3_ afforded a mixture of PhH_2_SiOMe and PhHSi(OMe)_2_. Instead, monoalkoxy species such as PhH_2_SiO*t*Bu, PhMeHSiO*i*Pr, PhMeHSiO*t*Bu, Ph_2_HSiO*i*Pr, and Ph_2_HSiO*t*Bu are synthesized by Zn‐catalyzed reactions of *i*PrOH or *t*BuOH. Secondary silanes easily provide monoalkoxy silane products, such as PhMeHSiO*i*Pr. Even in the presence of excess *i*PrOH after 1 day at room temperature, the tertiary silane Ph_2_HSiO*i*Pr is obtained rather than Ph_2_Si(O*i*Pr)_2_. Remarkably, the reactions involving *t*BuOH can be performed solvent‐free to access partially substituted silanes directly. Tertiary silanes such as triethylsilane and methyldiphenylsilane are inert toward dehydrocoupling reactions with methanol using **2 a** under these conditions. Clearly, tri‐substituted silane intermediates such as Ph(MeO)_2_SiH, PhMe(MeO)SiH, and Ph_2_(MeO)SiH are reactive toward MeOH in the presence of catalytic zinc. Because there are only minor differences in the steric properties at the silicon centers in Ph_2_MeSiH and Ph_2_(MeO)SiH, the greater reactivity of the monoalkoxy silane is most likely the result of its greater electrophilicity.

### Kinetics and mechanism of zinc‐catalyzed dehydrocoupling reactions

**Scheme 3:**
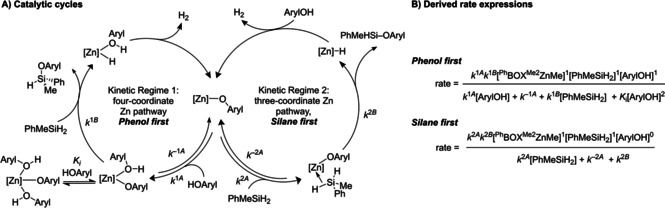
Proposed catalytic mechanisms and rate expressions for zinc‐catalyzed dehydrocoupling.

The kinetic behavior of **2 a**‐catalyzed dehydrocoupling of 3,5‐dimethylphenol and PhMeSiH_2_, as described below, indicates that this reaction follows two distinct mechanisms. The two proposed mechanisms are distinguished by coordination of the aryl alcohol (*Kinetic Regime 1: Phenol first*) or hydrosilane (*Kinetic Regime 2: Silane first*) to the zinc aryloxide catalytic species (Scheme 3). The relative concentration of aryl alcohol and silane substrates appears to be the primary factor that determines which pathway is dominant. In addition, the observed rate constants in both mechanistic regimes are affected by saturation behavior by one or both of the reactants. The saturation in organosilane leads to the important conclusion that a σ‐complex Zn↼H−Si forms prior to the σ‐bond metathesis step that produces the Si−O bond. Saturation kinetics in aryl alcohol indicates that a ZnOAryl(HOAryl) adduct is formed prior to creation of the Si−O bond under conditions of excess arylalcohol. We propose that these mechanisms are a consequence of the three‐coordinate nature of the monomeric ^Ph^BOX${}^{\mathrm{Me}{}_{2}}$ Zn−X catalytic species. This coordinative unsaturation allows the catalyst to adapt its structure to the reaction conditions, to maintain high reactivity.

First, initiation of the pre‐catalyst **2 a** involves its rapid reaction with ArylOH to produce the aryloxido‐zinc **3 c**, or with MeOH or *i*C_3_H_7_OH to give **3 a** or **3 b**, respectively, as described above. In contrast, solutions of **2 a** and PhMeSiH_2_ contained only starting materials after standing at room temperature for 4 d. In addition, NMR spectra and gas chromatograms of reaction mixtures lacking the ^Ph^BOX${}^{\mathrm{Me}{}_{2}}$ ZnMe pre‐catalyst revealed only PhMeSiH_2_ and ArylOH at their initial concentrations, and PhMeHSi−OAryl and H_2_ products were not detected. The rate laws determined under a wide range of reactant concentrations also show first‐order dependence on the initial [**2 a**], suggesting the active catalytic species is monomeric.

#### Kinetic Regime 1

**Figure 3:**
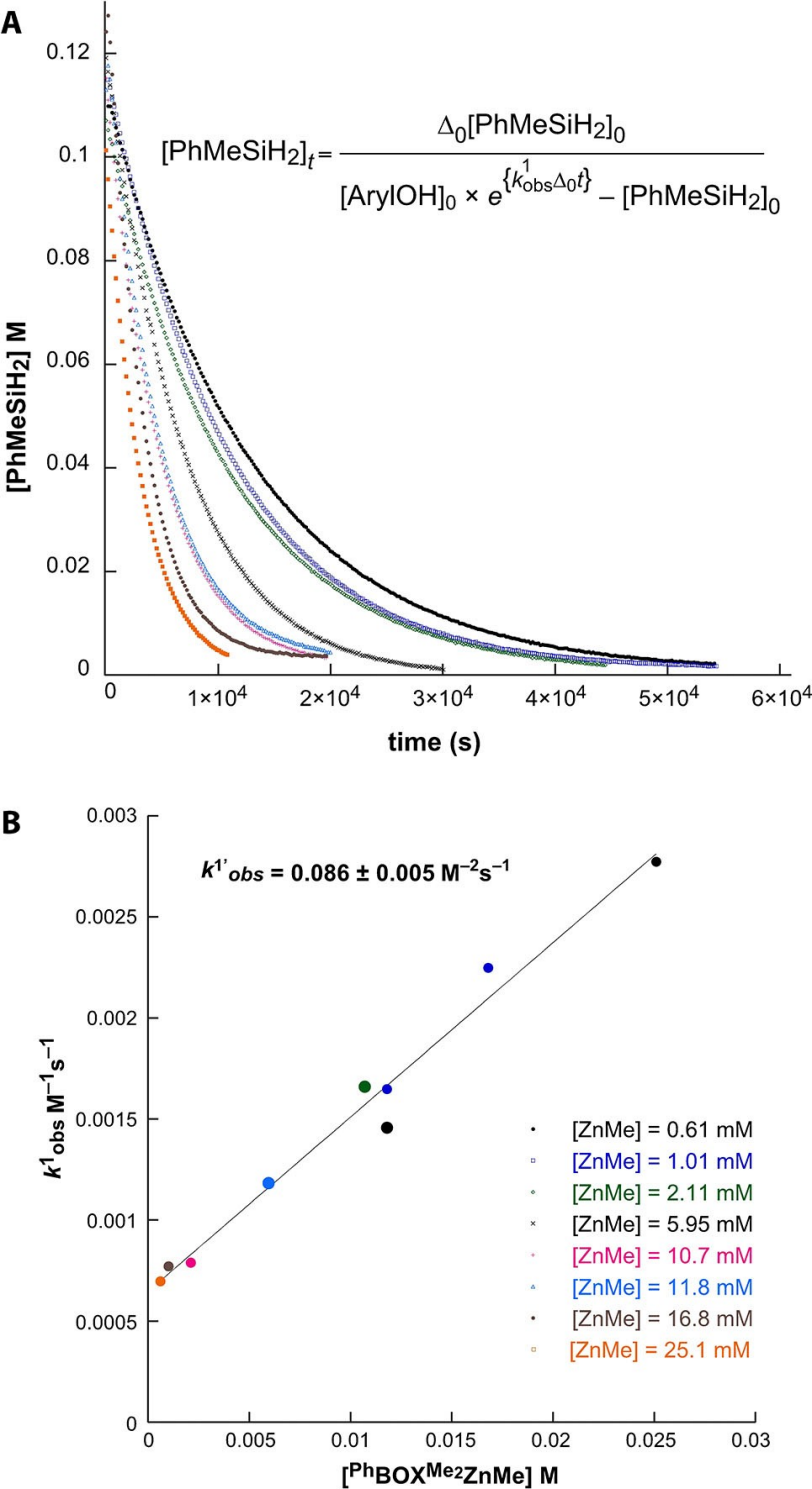
A) Plots of [PhMeSiH_2_] vs. time for its dehydrocoupling reaction with ArylOH catalyzed by ^Ph^BOX${}^{\mathrm{Me}{}_{2}}$ ZnMe at 60 °C, with catalytic concentration ranging from 1.14 to 25.1 mM. [ArylOH]_ini_=0.18±0.01 M, [PhMeSiH_2_]_ini_=0.115±0.008 M. B) Plot of second‐order rate constants k1obs vs. [**2 a**]. The slope corresponds to the ternary rate constant k1'obs =0.086±0.005 M^−2^s^−1^.

In the presence of excess 3,5‐dimethylphenol ([ArylOH]:[PhMeSiH_2_]=1.55 : 1, with [ArylOH]=0.18±0.01 M, 60 °C) plots of [PhMeSiH_2_] against time (Figure 3) analyzed by nonlinear least‐squares regression provide second‐order rate constants k1obs . A plot of second‐order rate constants k1obs against [**2 a**] from 2.1 to 25.1 mM reveals a linear correlation (Figure 3, inset), with the slope corresponding to the observed ternary rate constant k1'obs =0.086±0.005 M^−2^ s^−1^. The small but non‐zero value for the *y*‐intercept of 6×10^−4^ M^−1^ s^−1^ suggests a catalyst‐free background reaction, in conflict with the lack of background reaction under catalyst‐free conditions. Instead, the dependence of this *y*‐intercept value on [ArylOH] results from a [ArylOH]‐dependent displacement of the equilibrium between ^Ph^BOX${}^{\mathrm{Me}{}_{2}}$ ZnOAryl and ^Ph^BOX${}^{\mathrm{Me}{}_{2}}$ ZnOAryl(HOAryl). As further evidence, a subsequent series of kinetic experiments revealed that the slope and intercept of the plot of k1obs against [**2 a**] were affected by the concentration of 3,5‐dimethylphenol, with [ArylOH]/[PhMeSiH_2_]=8 : 1 giving a flatter slope (k1'obs =0.015 M^−2^ s^−1^) and smaller *y*‐intercept (7.6×10^−5^ M^−1^ s^−1^).

**Figure 4:**
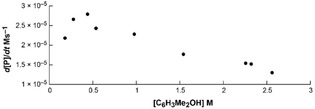
Plot of the initial rate of PhMeHSiOAryl formation vs. [ArylOH] showing decreasing rate constant.

Together, this observation and the unlikely represention of the ternary rate law by a single termolecular elementary step suggest a mechanism involving a two‐step sequence, in which the first reactant and the catalyst form an adduct in a reversible step, followed by reaction of the complex intermediate with the second reactant.^[57]^ This two‐step reaction mechanism is further supported by kinetic saturation of initial rates at high [ArylOH]. The initial rates of product formation (d[PhMeHSiOAryl]/d*t*) increase with increasing concentrations of 3,5‐dimethylphenol until 0.44 M, at which point the reaction rate decreases (Figure 4). The latter effect is attributed to catalyst inhibition by coordination of a second equivalent of phenol. This observation of inhibition by ArylOH provides additional important evidence reinforcing the proposed catalytically relevant sequence involving reversible coordination of one arylalcohol molecule to zinc, followed by reaction with hydrosilane. In particular, the reverse order (coordination by hydrosilane then Si−O bond formation by reaction with ArylOH) would require, unreasonably, ArylOH to simultaneously inhibit the intermediate and react with that same intermediate in a productive catalytic step.

An experimental rate law combining these observations is given in Equation (3), where k1cat corresponds to the product of the two forward steps, *K* ^1^_m_ is related to the Michaelis constant (rates consuming the intermediate divided by the rate constant for the first step), and K1inhib is the inhibition equilibrium constant.

(3) d[PhMeHSiOAryl]dt=k1cat[PhBOXMe2ZnMe]1[PhMeSiH2]1[ArylOH]11+K1m[ArylOH]+K1inhib[ArylOH]2

At low [ArylOH], a ternary rate law is observed, and the observed order of [ArylOH] dependence becomes zero and then inverse as its concentration increases.

#### Kinetic Regime 2

**Figure 5:**
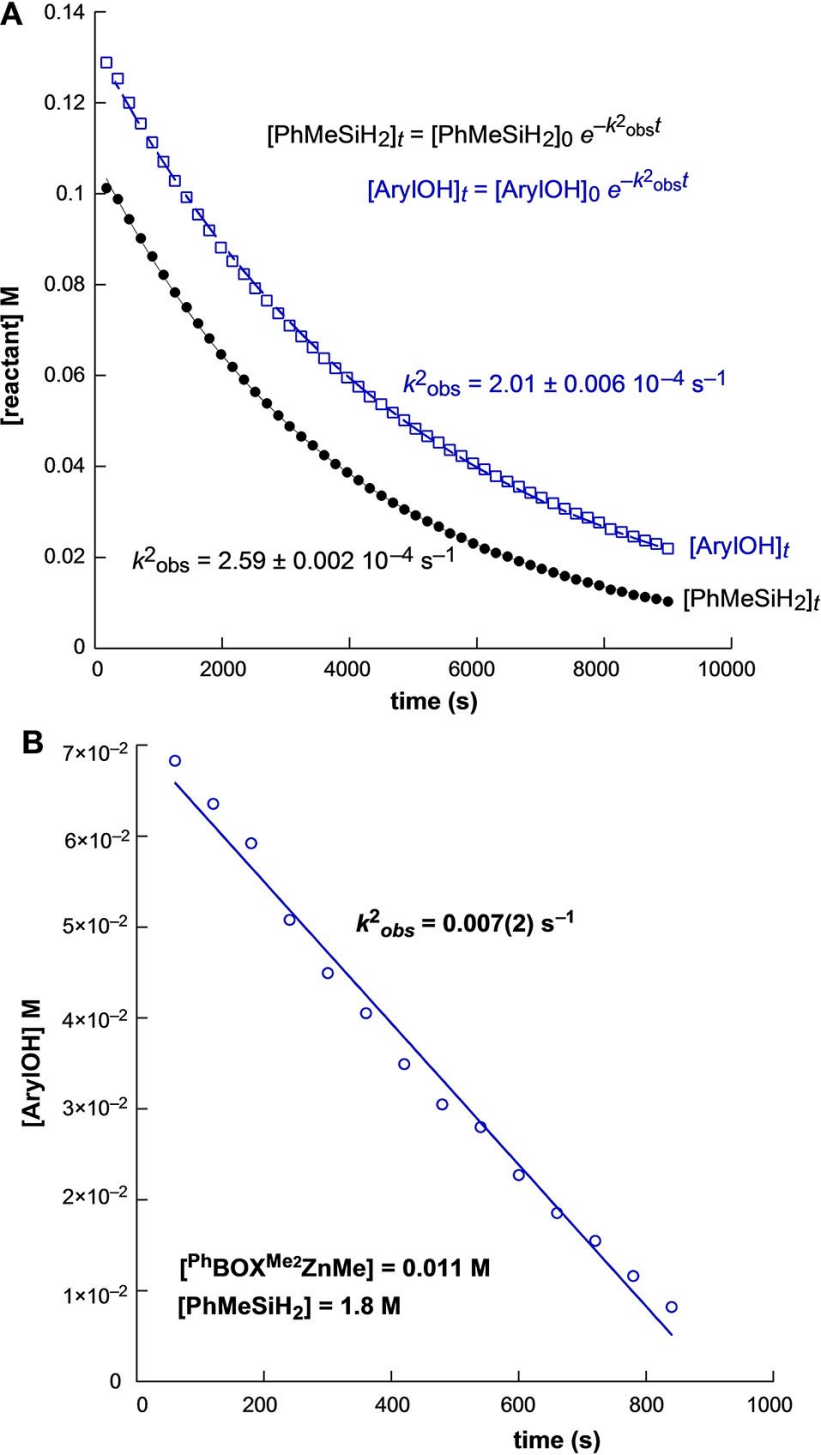
Plots of [ArylOH] and [PhMeSiH_2_] vs. time in **2 a**‐catalyzed dehydrocoupling reactions in Kinetic Regime 2 follow A) exponential time dependence indicative of first‐order behavior or B) linear time dependence indicative of zero‐order behavior, with the observed rate constant k2obs .

At lower ArylOH concentrations, the time dependences of both [PhMeSiH_2_] and [ArylOH] follow an exponential decay (Figure 5A; 1.4>[ArylOH]/[PhMeSiH_2_]>1.2; average [ArylOH]_ini_=0.12 M, average [PhMeSiH_2_]_ini_=0.096 M), indicating that the transformation is first‐order in only one of the reactants. These data indicate that either PhMeSiH_2_ or ArylOH is present in the turnover‐limiting step, in a remarkable contrast to the behavior in Kinetic Regime 1. Experiments varying [**2 a**], with [PhMeSiH_2_]_ini_ and [ArylOH]_ini_ kept constant, reveal first‐order dependence on catalyst concentration.

At high [PhMeSiH_2_]_ini_, **2 a**‐catalyzed dehydrocoupling shows zero‐order kinetic dependence on the ArylOH limiting reactant (Figure 5B). These data indicate that under conditions of moderate [PhMeSiH_2_]_ini_, the catalytic reaction follows the second‐order rate law of Equation (4) (k2'obs =9.8±0.4×10^−3^ M^−1^ s^−1^) in Kinetic Regime 2.

(4) -d[ArylOH]dt=k2'obs[2a]1[PhMeSiH2]1

For comparison, the observed second‐order rate constant for To^M^ZnH‐catalyzed dehydrocoupling is 0.014 M^−1^s^−1^ at 60 °C.^[25]^ Thus, the experimental rate law, rate constant, and reactivity, as well as Si−O bond formation as the turnover‐limiting step for **2 a**‐catalyzed dehydrocoupling of ArylOH and PhMeSiH_2_ in Kinetic Regime 2 are comparable to the catalytic features of four‐coordinate To^M^ZnH. A first major consequence, then, of the coordinative unsaturation of **2 a** is the creation of the new catalytic mechanism in Kinetic Regime 1, rather than increasing the catalytic rate.

**Figure 6:**
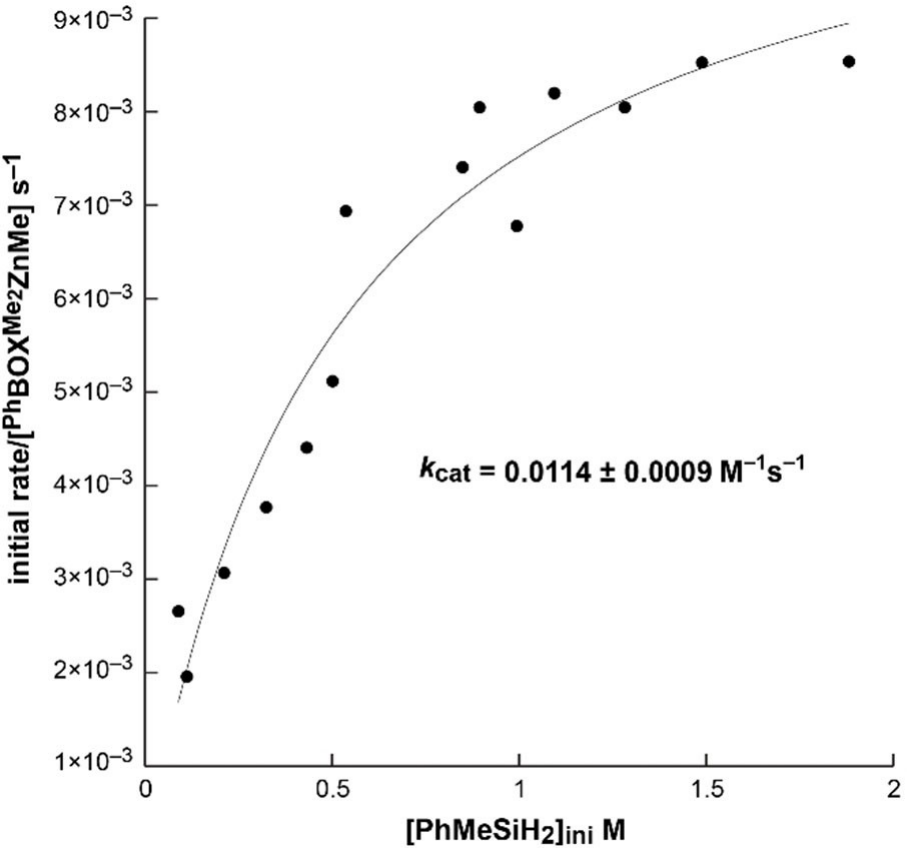
Initial rates vs. [PhMeSiH_2_]_ini_ reveals saturation behavior. k2SATobs (often defined as *k*_cat_) is equal to the rate at saturation (*v*_max_) divided by catalyst concentration.

Zero‐order dependence on [ArylOH] in Figure 5b, however, is ambiguous with respect to the dependence of rate on [PhMeSiH_2_] at that high concentration. Unexpectedly, initial rates of product formation (d[PhMeHSiOAryl]/d*t*) reveal saturation behavior as [PhMeSiH_2_]_ini_ is increased, giving zero‐order dependence on [PhMeSiH_2_] at high initial concentrations (Figure 6). Thus, Equation (4) represents the lower‐concentration limiting case of the bimolecular Michaelis‐Menten‐type description of the catalytic kinetics in Equation 5.

(5) $$\frac{d[\mathrm{PhMeHSiOAryl}]}{dt}=\frac{k{}_{\mathrm{cat}}\left[2\;a\right]{}^{1}\left[\mathrm{PhMeSiH}{}_{2}\right]{}^{1}}{K{}_{m}+\left[\mathrm{PhMeSiH}{}_{2}\right]}$$

This rate law is consistent with the bimolecular reaction of ^Ph^BOX${}^{\mathrm{Me}{}_{2}}$ ZnOAryl and PhMeSiH_2_ occurring in two steps: reversible association of PhMeSiH_2_ and ^Ph^BOX${}^{\mathrm{Me}{}_{2}}$ ZnOAryl to give an adduct, followed by extrusion of the product in an irreversible step (Scheme 3, right). These data, which lead to the revised mechanism of Kinetic Regime 2, reveal this second consequence of the coordinative unsaturation of pre‐catalyst **2 a**.

**Scheme 4:**
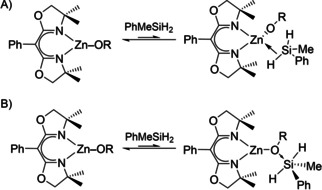
Possible equilibria that could lead to saturation kinetics in [PhMeSiH_2_].

The structure of the alkoxyzinc ⋅ silane adduct could involve hydrosilane coordination to the zinc center (Scheme 4a). As noted in the Introduction, M↼H−Si adducts prior to the four‐centered electrocyclic transition have been postulated in σ‐bond metathesis‐type on the basis of DFT calculations,^[46]^ but these species are typically fleeting.^[31]^ Although hydrosilane adducts to d^*n*^ transition metal centers (*n* ≠ 0) are well established,[^58^, ^59^, ^60^, ^61^, ^62^] including in silane/alcohol dehydrocoupling reactions,^[16]^ the core‐like 3d orbitals of zinc are unable to provide the stabilizing back‐donating interaction important to transition metal σ‐adducts.

Alternatively, coordination of the nucleophilic alkoxide to silicon would give a five‐coordinate silane adduct (Scheme 4b). Formation of this intermediate could also rationalize the higher reactivity of alkoxy‐substituted silanes through stabilization of the higher‐coordinate silicon center by electronegative substituents. Several experimental observations, however, disfavor this kind of intermediate. The formation of such a hyper‐coordinated silane intermediate does not require a coordinatively unsaturated metal center, which appears to be the significant feature of the chemistry of ^Ph^BOX${}^{\mathrm{Me}{}_{2}}$ ZnX. Moreover, the saturation in hydrosilane in Kinetic Regime 2 parallels saturation with excess ArylOH in Kinetic Regime 1, where coordination of phenol to zinc is reasonably established. Finally, the distinct rate laws of the two Kinetic Regimes suggest that their mechanisms are inequivalent, which would also imply that a zinc‐silane adduct is reasonable.

**Figure 7:**
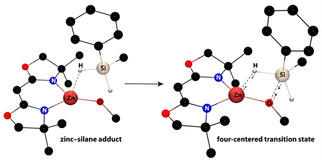
The calculated zinc ⋅ silane adduct is an intermediate leading to a four‐centered transition state associated with σ‐bond metathesis. The phenyl group and all H atoms bonded to carbon were included in the calculation, but not in the illustration for clarity.

DFT calculations also favor the Zn↼H−Si adduct over a hyper‐coordinate silane adduct preceding the four‐centered transition state during σ‐bond metathesis. In particular, the energy of the system increases as the alkoxyzinc oxgen approaches the silicon center of PhMeSiH_2_, and a local minimum involving a ZnO→SiPhMeH_2_ interaction could not be located. In contrast, the Zn↼H−Si, involving a side‐on interaction, is a local minimum (Figure 7). In particular, the Zn⋅⋅⋅H distance of 2.23 Å in this adduct is longer than Zn−H in three coordinate (DIPP‐nacnac)ZnH (1.46(2) Å; DIPP‐nacnac=HC{CHN(2,6‐C_6_H_3_ *i*Pr_2_)}_2_)^[63]^ and in four‐coordinate To^M^ZnH (1.52(2) Å)^[64]^ and [κ^3^‐Tptm]ZnH (1.51(3) Å).^[65]^ The calculated Zn⋅⋅⋅H, as well as Zn⋅⋅⋅Si and Si⋅⋅⋅O distances (3.32 and 2.95 Å, respectively), are also longer than the sum of covalent radii.^[66]^ The Zn↼H−Si angle (124.5°) is far from linear, the Zn−O⋅⋅⋅Si−H torsion is −11.8°, and the silicon center is tetrahedral. In addition, the terminal (1.48 Å) and bridging (1.50 Å) Si−H distances are very similar, although the calculated vibrational frequencies of 2147 and 2072 cm^−1^, respectively, are not equivalent.

**Scheme 5:**
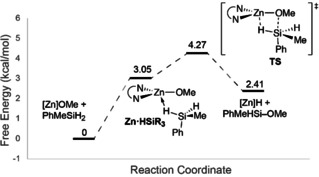
Reaction free energies (298.16 K) showing the σ‐bond metathesis step for Si−O bond formation.

This Zn↼H−Si adduct is connected, as shown by an IRC calculation, to the four‐centered transition state, which is characterized by one negative vibrational mode. The calculated energy coordinate diagram for Si−OMe bond formation is shown in Scheme 5. In the transition state, the Zn⋅⋅⋅H and O⋅⋅⋅Si distances are shortened (2.12 and 2.30 Å, respectively) and the Si−H and Zn−O distances are slightly longer (1.52 and 1.89 Å, respectively) than in the adduct. The calculated *ν*_SiH_ (1912 cm^−1^) of the moiety participating in the electrocyclic transition state is greatly reduced with respect to the non‐bridging ν_SiH_ (2117 cm^−1^). Remarkably, the negative mode corresponds to motion of O and Si along a vector connecting the two atoms. The Zn and H atoms are also moving along a vector connecting the two atoms. Although the Zn↔H motion is smaller in magnitude than the O↔Si motion, the two sets of motion are in phase.

## Conclusions

Three‐coordinate zinc complexes provide highly active and long‐lived catalysts for dehydrocoupling of primary or secondary silanes and alcohols. Selectivity for partial substitution to monoalkoxysilanes is influenced by the steric properties of the alcohol, and extremely fast and efficient conversion of primary silanes and methanol gives a high degree of substitution.

While the previously reported kinetic behavior of four‐coordinate To^M^ZnH/To^M^ZnOAryl implicates a simple, two‐step mechanism, comparison with ^Ph^BOX${}^{\mathrm{Me}{}_{2}}$ ZnMe‐initiated dehydrocoupling provides evidence for several accessible mechanisms. These mechanisms are established under conditions in which one reactant is in excess, as is often the case under pseudo‐first‐order conditions employed in kinetic studies. In contrast, typical reaction conditions for synthetic applications in these dehydrogenative cross‐couplings, as well in other cross‐coupling reactions, match the concentration of reagents to the desired stoichiometry of the conversion. Under such synthetic conditions, it is probable that both the phenol‐first and silane‐first pathways are concurrently operative. This variation of mechanism as a function of reactant concentrations has important consequences for assessing and comparing the performance of catalysts, in typical terms of activity and selectivity, and eventually designing more efficient and effective complexes.

The reactivity of ^Ph^BOx${}^{\mathrm{Me}{}_{2}}$ ZnX is dominated by its open coordination site, which is satisfied in ^Ph^BOX${}^{\mathrm{Me}{}_{2}}$ ZnOR′ by dimerization upon crystallization, formation of alkoxyzinc ⋅ alcohol intermediates at high alcohol concentrations, and formation of alkoxyzinc ⋅ silane intermediates at high hydrosilane concentrations. Kinetic experiments, corroborated by DFT calculations, provide powerful evidence in support of hydrosilane σ‐complexes as intermediates prior to the σ‐bond metathesis transition state.

We also note that phenol is a catalyst inhibitor at high concentration, as depicted in the phenol‐first cycle in Scheme 3. This inhibition, either by coordinating to zinc or hydrogen‐bonding to the reactive phenoxide group, likely blocks interaction with the hydrosilane reactant. Such structures undoubtably affect related catalytic transformations involving organozinc and coordinating reactants because the zinc center is able to effectively adapt its coordination sphere to reaction conditions.

## Conflict of interest

The authors declare no conflict of interest.

## Supporting information

As a service to our authors and readers, this journal provides supporting information supplied by the authors. Such materials are peer reviewed and may be re‐organized for online delivery, but are not copy‐edited or typeset. Technical support issues arising from supporting information (other than missing files) should be addressed to the authors.

Supplementary
