# Supplementary material for: Hydrosilane σ‐Adduct Intermediates in an Adaptive Zinc‐Catalyzed Cross‐dehydrocoupling of Si−H and O−H Bonds
Source: Chemistry. 2021 May 21;27(40):10428–36. doi: 10.1002/chem.202101146 (PMC8362191; doi:10.1002/chem.202101146)
Supplement: Supplementary file 1 — Supplementary [file CHEM-27-10428-s001.pdf]

Chemistry–A European Journal

Supporting Information

Hydrosilane σ-Adduct Intermediates in an Adaptive Zinc-Catalyzed Cross-dehydrocoupling of Si–H and O–H Bonds

Smita Patnaik, Uddhav Kanbur, Arkady Ellern, and Aaron D. Sadow*

Author Contributions

S.P. Investigation:Lead; Writing – original draft:Lead
U.K. Investigation:Supporting; Writing – review & editing:Supporting
A.E. Investigation:Supporting
A.S. Conceptualization:Lead; Writing – original draft:Lead; Writing – review & editing:Lead

Table of Contents

General Experimental Section S-1

Synthesis of Compounds S-2

Catalytic Dehydrocoupling Experiments S-5

Silane Products from Catalytic Dehydrocoupling Reactions S-5

Computational Details S-7

NMR and IR spectra of **2a-b** and **3a-c** S-8

NMR Spectra of Silane Products S-25

Procedure for Kinetic Measurements S-43

Representative Kinetic Plots S-46

References S-58

General Experimental Section

All manipulations were performed under a dry argon atmosphere using standard Schlenk techniques or under a nitrogen atmosphere in a glovebox unless otherwise indicated. Water and oxygen were removed from benzene and pentane solvents using an IT PureSolv system. Benzene-d_6 and THF-d_8 were heated to reflux over Na/K, and vacuum-transferred. Dimethyl zinc and diethyl zinc were purchased from Sigma Aldrich and were used as received. Products of catalytic hydroboration were assigned based on comparison with authentic samples and/or literature values. ^{1}H, ^{13}C{^{1}H}, ^{15}N{^{1}H} HMBC and ^{29}Si{^{1}H} HMBC NMR spectra were collected on a Bruker DRX-400 spectrometer, a Bruker Avance III-600 spectrometer, or an Agilent MR 400 spectrometer. Infrared spectra were measured on a Bruker Vertex 80. ^{15}N chemical shifts obtained via ^{15}N-^{1}H HMBC experiments, originally referenced to liquid NH_3, were re-referenced to CH_3NO_2 by subtracting 381.9 ppm. Elemental analyses were performed using a Perkin-Elmer 2400 Series II CHN/S in the Chemical Instrumentation Facility (CIF) at Iowa State University.

Synthesis of Compounds

Benzylene-bis(4,4-dimethyl-2-oxazoline) (^{Ph,H}BOX^{Me2}). ^{Ph,H}BOX^{Me2} was prepared following the literature procedure,[1] and spectroscopic data matched literature values. Analytical data was not previously reported. Yield 17 g (59 mol, 70%). Anal. Calc. for: C, 71.30; H, 7.74; N, 9.78. Found: C, 70.94; H, 7.72; N, 9.64. Mp: 195-197 °C.

^{Ph}BOX^{Me2}ZnMe (2a). ^{Ph,H}BOX^{Me2} (0.100 g, 0.349 mmol) and $ZnMe_2$ (0.170 mL, 0.349 mmol, 2 M in toluene) were allowed to react in a stirred benzene (5 mL) solution at room temperature over 12 h. Evaporation of the volatile materials afforded ^{Ph}BOX^{Me2}ZnMe as a white powder in good yield. This solid was recrystallized at –30 °C from a minimal amount of pentane to obtain colorless X-ray quality crystals. Yield 0.120 g, 0.331 mmol, 95%. ^{1}H NMR (benzene-d_6, 600 MHz, 25 °C): δ –0.09 (s, 3 H, ZnMe), 1.02 (s, 12 H, NCMe_2CH$_2$O), 3.42 (s, 4 H, NCMe$_2$CH_2O), 7.18 (br, 1 H, *p*-C_6H_5), 7.36 (m, 2 H, *m*-C_6H_5), 7.68 (m, 2 H, *o*-C_6H_5). ^{13}C{^{1}H} NMR (benzene-d_6, 150 MHz, 25 °C): δ –14.68 (ZnMe), 28.80 (CNCMe_2CH$_2$O), 63.48 (CNCMe$_2$CH$_2$O), 74.00 ($C_6H_5$$C$), 77.82 (CNCMe$_2$$CH_2$O), 125.16 ($C_6H_5$), 127.32 ($C_6H_5$), 132.64 ($C_6H_5$), 137.20 ($C_6H_5$), 169.37 ($C$NCMe$_2CH_2$O). ^{15}N NMR (benzene-$d_6$, 60.8 MHz, 25 °C): δ –213. IR (KBr, cm^{-1}): 663 (s), 699 (s), 742 (m), 755 (m), 816 (m), 933 (w), 1043 (s), 1171 (m), 1193 (s), 1252 (m), 1321 (m), 1365 (m), 1401 (m), 1437 (s), 1453 (s), 1490 (s), 1609 (s, ν_{CN}), 2839 (w), 2879 (m), 2902 (w), 2924 (m), 2968 (m), 2994 (s), 3053 (w), 3077 (w). Anal. Calc. for: C, 59.11; H, 6.61; N, 7.66. Found: C, 58.80; H, 6.65; N, 7.53. Mp: 208-210 °C. CCDC Deposition Number: 2062850.

^{Ph}BOX^{Me2}ZnEt (2b). ^{Ph,H}BOX^{Me2} (0.100 g, 0.349 mmol) and $ZnEt_2$ (0.170 mL, 0.349 mmol) were allowed to react in benzene (5 mL) for 12 h at room temperature. The volatile materials were evaporated under reduced pressure to afford ^{Ph}BOX^{Me2}ZnEt as a white powder in good yield. This solid was recrystallized at –30 °C from a minimal amount of pentane to obtain colorless X-ray quality crystals (0.122 g, 0.321 mmol, 92%). ^{1}H NMR (benzene-d_6, 600 MHz, 25 °C): δ 0.77 (q, 2 H, $^{3}J_{HH}$ = 7.4 Hz, ZnCH_2CH$_3$) 1.03 (s, 12 H, NCMe_2CH$_2$O), 1.64 (t, 3 H, $^{3}J_{HH}$ = 7.4 Hz, ZnCH$_2$CH_3), 3.42 (s, 4 H, NCMe$_2$CH_2O), 7.17 (d, 1 H, $^{3}J_{HH}$ = 7.1 Hz, *p*-C_6H_5), 7.37 (m, 2 H, *m*-C_6H_5), 7.69 (d, 2 H, $^{3}J_{HH}$ = 8.5 Hz, *o*-C_6H_5). ^{13}C{^{1}H} NMR (benzene-d_6, 150 MHz, 25 °C): δ –0.44 (ZnCH$_2$CH$_3$), 13.12 (ZnCH$_2$$CH_3$), 28.88 (CNC$Me_2CH_2$O), 63.44 (CN$CMe_2CH_2$O), 73.95 ($C_6H_5$$C$), 77.79 (CNCMe$_2$$CH_2$O), 125.18 ($C_6H_5$), 127.35 ($C_6H_5$), 132.66 ($C_6H_5$), 137.22 ($C_6H_5$),

169.32 (CNCMe$_2$CH$_2$O). ^{15}N NMR (benzene-d_6, 60.8 MHz, 25 °C): δ –214. IR (KBr, cm^{-1}): 661 (m), 697 (m), 760 (w), 802 (w), 851 (w), 990 (w), 1008 (m), 1051 (s), 1071 (s), 1117 (w), 1137 (w), 1180 (w), 1212 (w), 1235 (w), 1248 (w), 1278 (w), 1312 (w), 1337 (w), 1388 (m), 1413 (m), 1457 (s), 1491 (s), 1606 (s), 2872 (m), 2904 (m), 2924 (m), 2962 (s), 3030 (w), 3054 (w), 3080 (w). Anal. Calc. for: C, 60.09; H, 6.90; N, 7.38. Found: C, 59.88; H, 6.90; N, 7.17. Mp: 210-212 °C. CCDC Deposition Number: 2062849.

[^{Ph}BOX^{Me2}Zn(μ-OMe)]$_2$ (3a)$_2$. Methanol (0.026 g, 0.820 mmol, 0.032 mL) was added to a solution of ^{Ph}BOX^{Me2}ZnMe (0.100 g, 0.273 mmol) in benzene. The reaction mixture was stirred for 30 min. at room temperature. Evaporation of the volatile materials afforded [^{Ph}BOX^{Me2}Zn(μ-OMe)]$_2$ as a white powder in good yield (0.1 g, 0.259 mmol, 95%). ^{1}H NMR (chloroform-d, 600 MHz, 25 °C): δ 1.45 (s, 12 H, NCMe_2CH$_2$O), 3.65 (s, 3 H, OMe), 3.87 (s, 4 H, NCMe$_2$CH_2O), 7.09 (m, 1 H, C$_6$H$_5$), 7.31-7.20 (m, 4 H, C$_6$H$_5$). ^{13}C{^{1}H} NMR (chloroform-d, 150 MHz, 25 °C): δ 29.02 (CNCMe_2CH$_2$O), 54.49 (OMe), 63.89 (CNCMe$_2$CH$_2$O), 71.33 (C$_6$H$_5$$C$), 78.67 (CNCMe$_2$$CH_2$O), 124.99 ($p$-C$_6H_5$), 127.20 (C$_6H_5$), 132.14 (C$_6H_5$), 137.32 (C$_6H_5$), 168.96 ($C$NCMe$_2CH_2$O). ^{15}N NMR (chloroform-d, 60.8 MHz, 25 °C): δ –225. IR (KBr, cm^{-1}): 699 (m), 743 (m), 756 (m), 818 (m), 936 (w), 962 (w), 1056 (s), 1194 (s), 1251 (m), 1318 (s), 1365 (m), 1387 (m), 1407 (m), 1458 (s), 1494 (s), 1608 (s, ν_{CN}), 2802 (m), 2874 (m), 2897 (m), 2926 (m), 2967 (m), 3027 (s), 3043 (m), 3079 (w). Anal. Calc. for: C, 56.63; H, 6.34; N, 7.34. Found: C, 56.61; H, 6.41; N, 7.22. Mp: 235 °C (dec). CCDC Deposition Number: 2062852.

[^{Ph}BOX^{Me2}Zn(μ-OiC$_3$H$_7$)]$_2$ (3b)$_2$. iC$_3$H$_7$OH (0.050g, 0.820mmol, 0.062mL) was added to a solution of ^{Ph}BOX^{Me2}ZnMe (0.100 g, 0.273 mmol) in benzene. The reaction was stirred for 30 min. at room temperature. Evaporation of the volatile materials afforded [^{Ph}BOX^{Me2}Zn(μ-OiC$_3$H$_7$)]$_2$ as a white powder in good yield (0.103 g, 0.251 mmol, 92%). This solid was recrystallized from a minimal amount of methylene chloride at –30 °C to obtain colorless X-ray quality crystals. ^{1}H NMR (chloroform-d, 600 MHz, 25 °C): δ 1.08 (d, $^3J_{HH}$ = 5.9 Hz, 2 H, ZnOCHMe_2), 1.40 (s, 12 H, CNCMe_2CH$_2$O), 3.78 (s, 4 H, CNCMe$_2$CH_2O), 4.13 (sep, 1 H, ZnOCHMe$_2$), 7.04 (m, 1 H, C$_6$H$_5$), 7.19-7.15 (b, 4 H, C$_6$H$_5$). ^{13}C{^{1}H} NMR (chloroform-d, 150 MHz, 25 °C): δ 28.56 (ZnOCHMe_2), 29.64 (CNCMe_2CH$_2$O), 64.35 (CNCMe$_2$CH$_2$O), 66.05 (ZnOCHMe$_2$), 71.60 (C$_6$H$_5$$C$), 78.67 (CNCMe$_2$$CH_2$O), 125.04 (C$_6H_5$), 127.31 (C$_6H_5$), 132.44 (C$_6H_5$), 137.75 (C$_6H_5$), 168.89

(CNCMe$_2$CH$_2$O). ^{15}N NMR (chloroform-*d*, 60.8 MHz, 25 °C): δ –225. IR (KBr, cm^{-1}): 501 (w), 522 (w), 577 (w), 662 (w), 699 (w), 740 (w), 759 (w), 817 (w), 935 (m), 970 (s), 1021 (m), 1057 (s), 1129 (m), 1193 (m), 1252 (m), 1318 (m), 1366 (m), 1406 (m), 1441 (m), 1461 (m), 1495 (s), 1606 (s, ν_{CN}), 2876 (w), 2925 (m), 2961 (s). Anal. Calc. for: C, 58.61; H, 6.89; N, 6.84. Found: C, 58.37; H, 6.84; N, 6.68. Mp: 295 °C (dec). CCDC Deposition Number: 2062851.

[^{Ph}BOX^{Me2}Zn(μ-O-2,6-$C_6H_3Me_2$)]$_2$ (3c)$_2$. 3,5-Dimethyl phenol (0.050 g, 0.410 mmol) was added to a solution of ^{Ph}BOX^{Me2}ZnMe (0.100 g, 0.273 mmol) in benzene. The reaction was stirred for 30 minutes at room temperature. Evaporation of the volatile materials afforded [^{Ph}BOX^{Me2}Zn(μ-OC$_6$H$_3$Me$_2$)]$_2$ as a white powder in good yield (0.122 g, 0.259 mmol, 95%). ^{1}H NMR (chloroform-*d*, 600 MHz, 25 °C): δ 1.33 (s, 12 H, NCMe_2CH$_2$O), 2.26 (s, 6 H, C$_6$H$_3$$Me_2$), 3.94 (s, 4 H, NCMe$_2CH_2$O), 6.37 (s, 2 H, OC$_6$$H_3Me_2$), 6.43 (s, 1 H, OC$_6$$H_3Me_2$), 7.24 (m, 1 H, *p*-C$_6H_5$), 7.38 (m, 2 H, *m*-C$_6H_5$), 7.44 (d, $^3J_{HH}$ = 7.6 Hz, 2 H, *o*-C$_6$H$_5$). ^{13}C{^{1}H} NMR (chloroform-*d*, 150 MHz, 25 °C): δ 21.54 (OC$_6$H$_3$$Me_2$), 29.12 (NC$Me_2CH_2$O), 64.24 (NC$CMe_2CH_2$O), 72.28 (C$_6H_5$$C$), 78.85 (CNCMe$_2$$CH_2$O), 116.57 ($C_6H_3Me_2$), 119.70 (OC$_6H_3$), 125.45 (C$_6H_5$), 127.51 (C$_6H_5$), 132.40 (C$_6H_5$), 137.15 (C$_6H_5$), 138.71 (*m*-$C_6H_3Me_2$), 160.60 ($C_6H_3Me_2$), 169.48 ($C$NCMe$_2CH_2$O). ^{15}N NMR (chloroform-*d*, 60.8 MHz, 25 °C): δ –227.28. IR (KBr, cm^{-1}): 522 (w), 602 (w), 621 (m), 662 (m), 698 (m), 740 (m), 759 (m), 817 (m), 828 (m), 939 (m), 949 (m), 1059 (s), 1167 (s), 1194 (s), 1252 (s), 1316 (s), 1369 (s), 1382 (s), 1408 (w), 1442 (w), 1459 (m), 1492 (s), 1594 (s, ν_{CN}), 2892 (m), 2919 (m), 2969 (m), 3023 (m), 3051 (m), 3080 (m), 3594 (w), 3626 (w), 3705 (w), 3731 (w). Anal. Calc. for: C, 63.63; H, 6.41; N, 5.94. Found: C, 63.48; H, 6.32; N, 5.78. Mp: 340 °C (dec).

Catalytic Dehydrocoupling Experiments

A representative procedure for the catalytic dehydrocoupling of silanes and alcohols using $^{Ph}BOX^{Me2}ZnMe$ in benzene as solvent is given. Methanol (0.100 g, 3.15 mmol, 0.127 mL) was added to a solution of **2a** (0.032 g, 0.09 mmol) in benzene-d_6 containing tetrakis(trimethylsilyl)silane (0.01 M) as an internal standard. Phenylsilane (0.100 g, 0.9 mmol, 0.110 mL) was added to the reaction mixture, and the homogeneous solution was mixed on a vortex stirrer at room temperature. The progress of the reaction was monitored using ^1H NMR spectroscopy, specifically observing the appearance of new signals assigned to the alkoxy groups of the product and disappearance of SiH signal of the phenylsilane.

Solvent-free conditions: A mixture of methanol (0.100 g, 3.15 mmol, 0.127 mL) and phenylsilane (0.100 g, 0.9 mmol, 0.110 mL) was added to **2a** (0.032 g, 0.09 mmol). The reaction mixture becomes effervescent. The progress of the homogeneous reaction was monitored by removing small aliquots for analysis by ^1H NMR spectroscopy.

Conditions for high turnovers: Methanol (0.100 g, 3.15 mmol, 0.127 mL) and phenylsilane (0.100 g, 0.9 mmol, 0.110 mL) were added to $^{Ph}BOX^{Me2}ZnMe$ (diluted to 9×10^{-8} mmol; 3.8×10^{-7} M). The homogeneous reaction mixture was allowed to stir for 5 d at room temperature.

Silane Products from Catalytic Dehydrocoupling Reactions

$PhSi(OMe)_3$. ^1H NMR matches literature report in chloroform-d.[2] ^1H NMR given here in benzene-d_6. ^1H NMR (benzene-d_6, 600 MHz, 25 °C): δ 3.48 (s, 9 H, OMe), 7.20 (m, 3 H, C_6H_5), 7.80 (m, 3 H, C_6H_5) $^{13}C\{^1H\}$ NMR (benzene-d_6, 150 MHz, 25 °C): δ 50.25 (O*Me*). 127.89 (C_6H_5), 127.98 (C_6H_5), 130.39 (C_6H_5), 134.89 (C_6H_5).

$PhSi(O\textit{i}Pr)_3$. ^1H NMR matches literature report.[3] ^1H NMR (benzene-d_6, 600 MHz, 25 °C): δ 1.23 (d, $^3J_{HH}$ = 6.2 Hz, 18 H, Me), 4.35 (m, 3 H, C*H*), 7.24 (m, 3 H, C_6H_5), 7.93 (m, 2 H, C_6H_5). $^{13}C\{^1H\}$ NMR (benzene-d_6, 100 MHz): δ 134.9 (C_6H_5), 133.4 (C_6H_5), 129.9 (C_6H_5); 65.3 (OC*H*Me_2), 25.4 ($OCHMe_2$).

PhMeSi(OMe)$_2$. ^{1}H NMR matches literature report in chloroform-*d*.[4] ^{1}H NMR (benzene-d_6, 600 MHz, 25 °C): δ 0.30 (s, 3 H, SiMe), 3.39 (s, 6 H, OMe), 7.22 (m, 3 H, C_6H_5), 7.72 (m, 2 H, C_6H_5). ^{13}C{^{1}H} NMR (benzene-d_6, 150 MHz, 25 °C): δ –5.18 (SiH*Me*), 49.90 (OMe), 127.98 (C_6H_5), 129.43 (C_6H_5), 134.12 (C_6H_5), 134.41 (C_6H_5).

PhMeHSiO*i*Pr. ^{1}H NMR matches literature report.[3] ^{1}H NMR (benzene-d_6, 600 MHz, 25 °C): δ 0.37 (s, 3 H, SiH*Me*), 1.09 (m, 6 H, OCHMe_2), 3.94 (m, 1 H, OC*H*Me$_2$), 5.28 (s, 1 H, SiH), 7.21 (m, 3 H, C_6H_5), 7.63 (m, 2 H, C_6H_5). ^{13}C{^{1}H} NMR (benzene-d_6, 150 MHz, 25 °C): δ –2.23 (SiHMe), 25.07 (O*C*HMe$_2$), 66.56 (OCHMe_2), 127.91 (C_6H_5), 129.87 (C_6H_5), 133.86 (C_6H_5), 136.69 (C_6H_5).

PhMeHSiO*t*Bu.[5] ^{1}H NMR (benzene-d_6, 600 MHz, 25 °C): δ 0.39 (d, $^3J_{HH}$ = 3.1 Hz, 3 H, SiMe), 1.22 (s, 9 H, OCMe$_3$), 5.41 (m, 1 H, SiH), 7.22 (m, 3 H, C_6H_5), 7.66 (m, 2 H, C_6H_5). ^{13}C{^{1}H} NMR (benzene-d_6, 150 MHz, 25 °C): δ –0.47 (SiHMe), 31.21 (OCMe_3), 72.67 (O*C*Me$_3$), 128.07 (C_6H_5), 129.61 (C_6H_5), 133.71 (C_6H_5), 138.11 (C_6H_5).

PhMeHSiOC$_6$H$_3$Me$_2$.[6] ^{1}H NMR (benzene-d_6, 600 MHz, 25 °C): δ 0.43 (d, $^2J_{HH}$ = 3.25 Hz, 3 H, SiH*Me*), 2.05 (s, 6 H, $C_6H_3Me_2$), 5.54 (q, $^3J_{HH}$ = 3.2 Hz, 1 H, Si*H*Me), 6.50 (s, 1 H, C$_6$*H*$_3$Me$_2$), 6.73 (s, 2 H, C$_6$*H*$_3$Me$_2$), 7.16 (m, 3 H, C_6H_5), 7.62 (m, 2 H, C_6H_5). ^{13}C{^{1}H} NMR (benzene-d_6, 150 MHz, 25 °C): δ –2.23 (SiHMe), 20.91 ($C_6H_3Me_2$), 127.45 (*C*$_6$H$_3$Me$_2$), 127.69 (*C*$_6$H$_3$Me$_2$), 130.25 (C_6H_5), 133.86 (C_6H_5), 135.09 (C_6H_5), 139.11 (C_6H_5), 155.63 (*ipso*-C$_6$H$_3$Me$_2$).

Ph$_2$Si(OMe)$_2$. ^{1}H NMR spectrum in chloroform-*d* matches literature report.[7] ^{1}H NMR (benzene-d_6, 600 MHz, 25 °C): δ 3.46 (s, 6 H, OMe), 7.20 (m, 6 H, C_6H_5), 7.81 (m, 4 H, C_6H_5). ^{13}C{^{1}H} NMR (benzene-d_6, 150 MHz, 25 °C): δ 50.26 (OMe), 127.98 (C_6H_5), 130.23 (C_6H_5), 132.74 (C_6H_5), 135.03 (C_6H_5).

Ph$_2$HSiO*i*Pr. ^{1}H NMR matches literature report.[3] ^{1}H NMR (benzene-d_6, 600 MHz, 25 °C): δ 1.13 (d, $^3J_{HH}$ = 7.1 Hz, 6 H, OCHMe_2), 4.07 (m, 1 H, OC*H*Me$_2$), 5.71 (s, 1 H, SiH), 7.18 (m, 5 H, C_6H_5),

7.71 (m, 5 H, C_6H_5). $^{13}C\{^1H\}$ NMR (benzene-d_6, 150 MHz, 25 °C): δ 25.09 (OCH*Me*$_2$), 67.08 (O*C*HMe$_2$), 27.95 (C_6H_5), 130.11 (C_6H_5), 134.69 (C_6H_5), 134.89 (C_6H_5).

Ph_2HSiO*t*Bu. ^1H NMR spectrum in chloroform-*d* matches literature report.[8] ^1H NMR (benzene-d_6, 600 MHz, 25 °C): δ 1.26 (s, 9 H, OCMe$_3$), 5.82 (s, 1 H, SiH), 7.18 (m, 6 H, C_6H_5), 7.72 (m, 4 H, C_6H_5). $^{13}C\{^1H\}$ NMR (benzene-d_6, 150 MHz, 25 °C): δ 31.22 (OC*Me*$_3$), 73.23 (O*C*Me$_3$), 127.89 (C_6H_5), 129.86 (C_6H_5), 134.57 (C_6H_5), 136.21 (C_6H_5).

Computational Details

The ground state geometry optimization, IR frequency and saddle point calculations were performed using Truhlar's Minnesota 06-2X meta-GGA functional,[9] as implemented in NWChem.[10] The Los Alamos National Laboratory double-ζ valence basis set (LANL2DZ)[11] was used along with effective core potentials (ECPs) for Zn and Si. Grimme's dispersion corrections were empirically added through a long-range contribution (DFT-D3) for all calculations.[12] Hessians were performed on all optimized structures. Starting materials, intermediates, and products contain no imaginary (negative) modes. Transition-state structures (saddle points) contained exactly one negative frequency mode. All ground state structures IR frequencies were corrected by a scaling factor of 0.960.

NMR and IR spectra of 2a-b and 3a-c

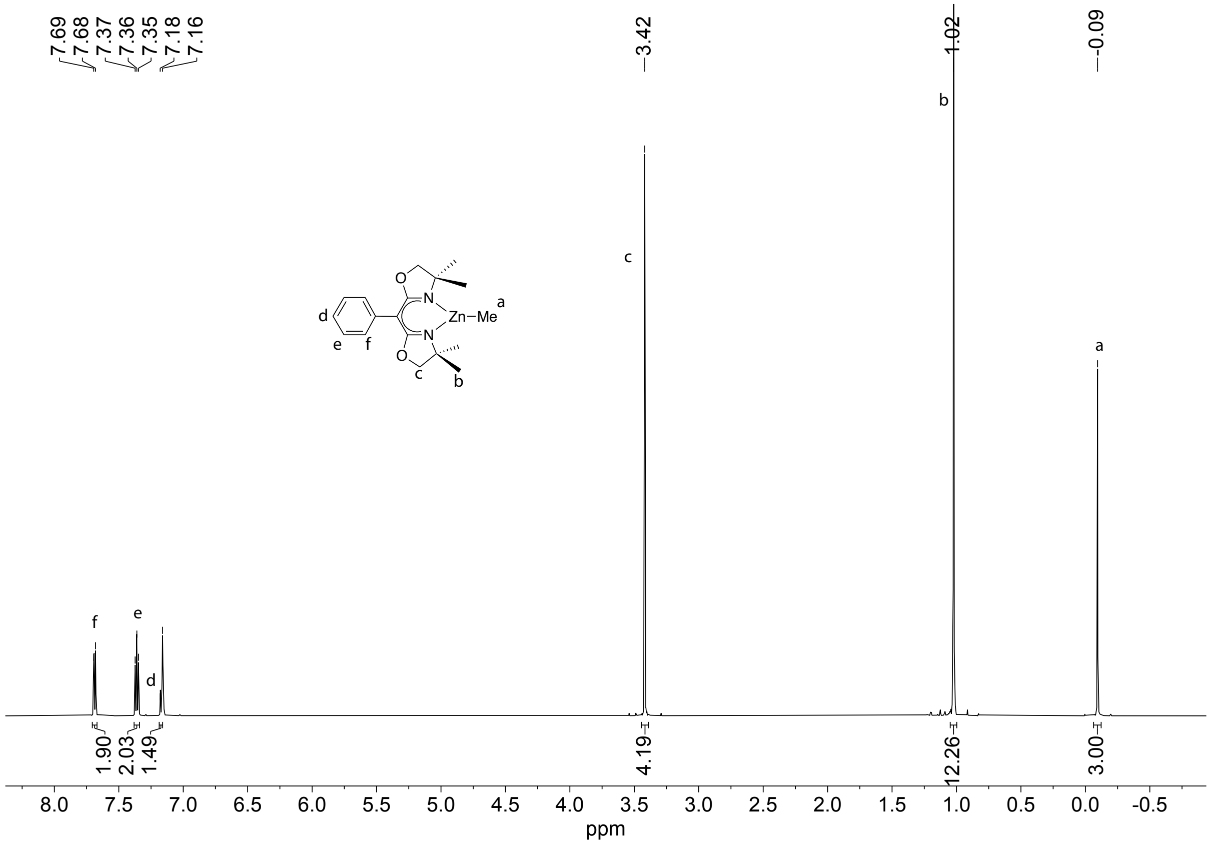

Figure S1. ^{1}H NMR spectrum of ^{Ph}BOX^{Me2}ZnMe (**2a**) acquired in benzene-d_6.

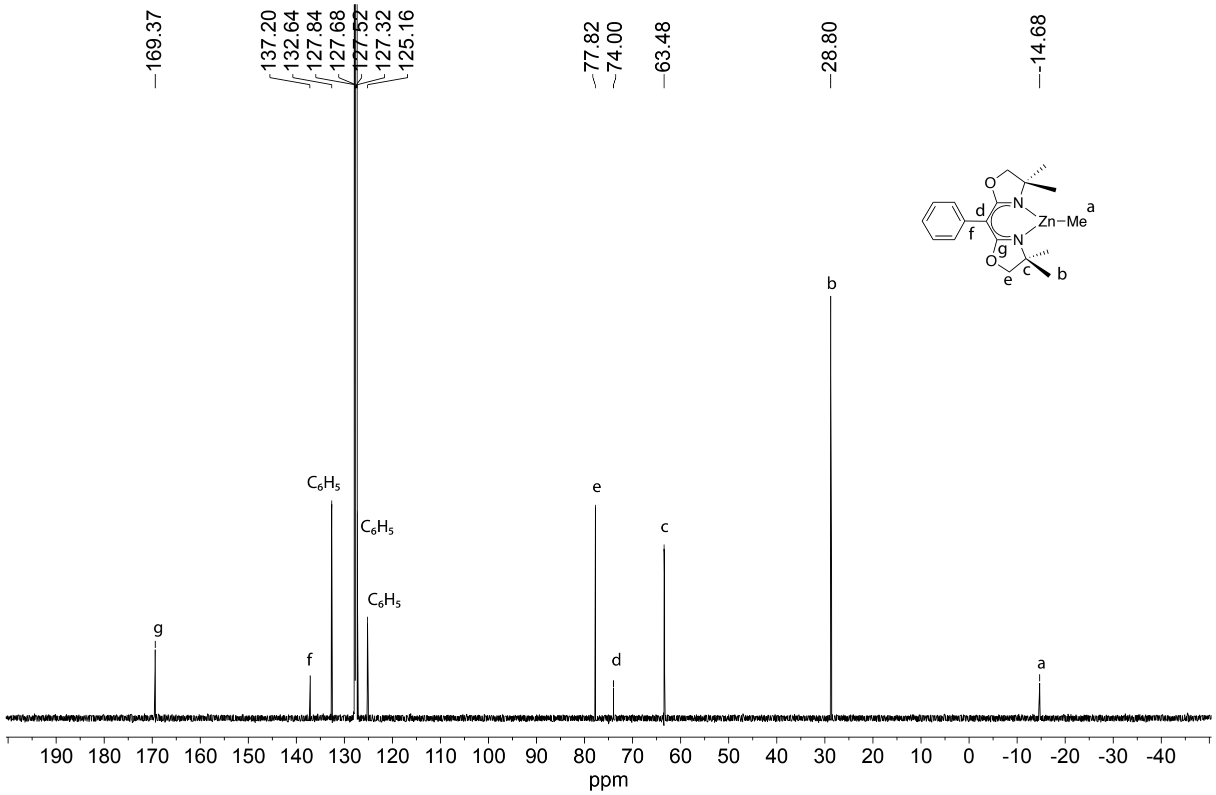

Figure S2. $^{13}C\{^1H\}$ NMR spectrum of $^{Ph}BOX^{Me2}ZnMe$ (**2a**) acquired in benzene-d_6.

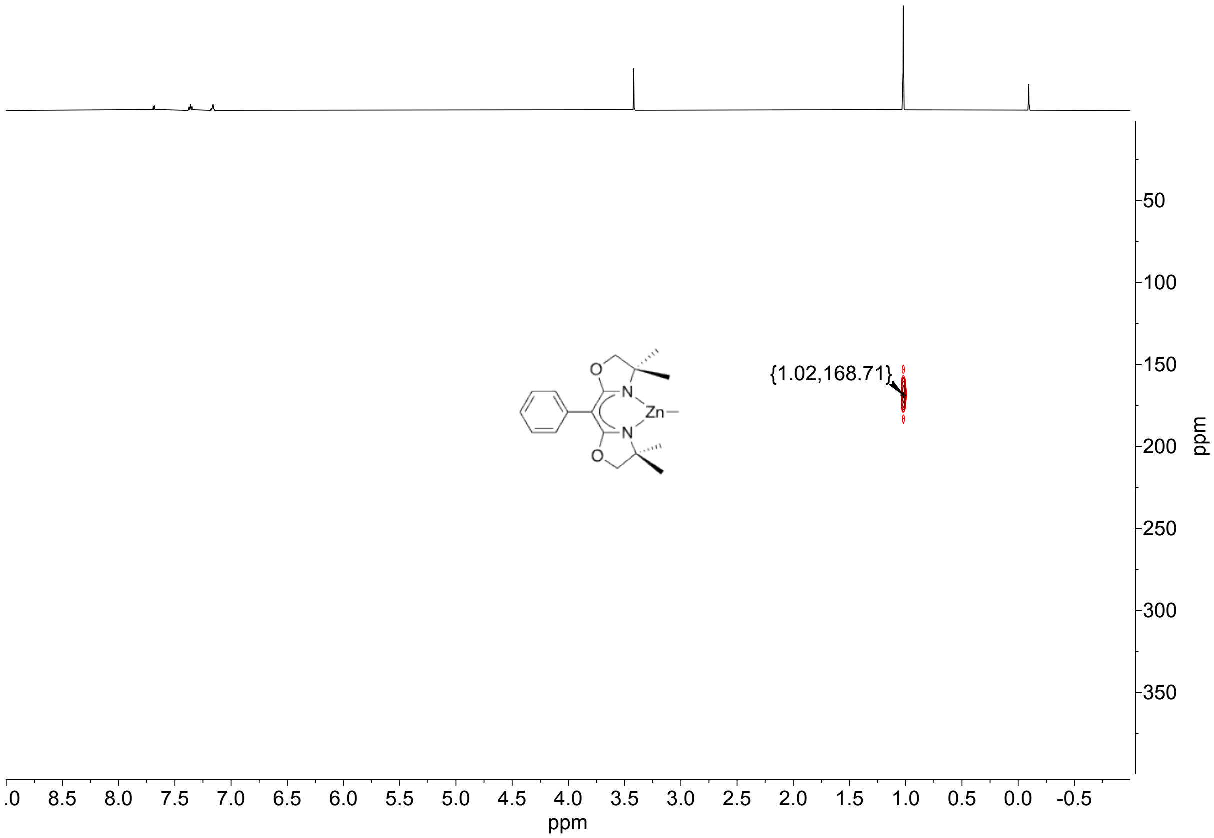

Figure S3. ^{15}N-^{1}H HMBC spectrum of $^{Ph}BOX^{Me2}ZnMe$ (**2a**) acquired in benzene-d_6. Recalibration to nitromethane as an external standard gives $\delta_N = -213$.

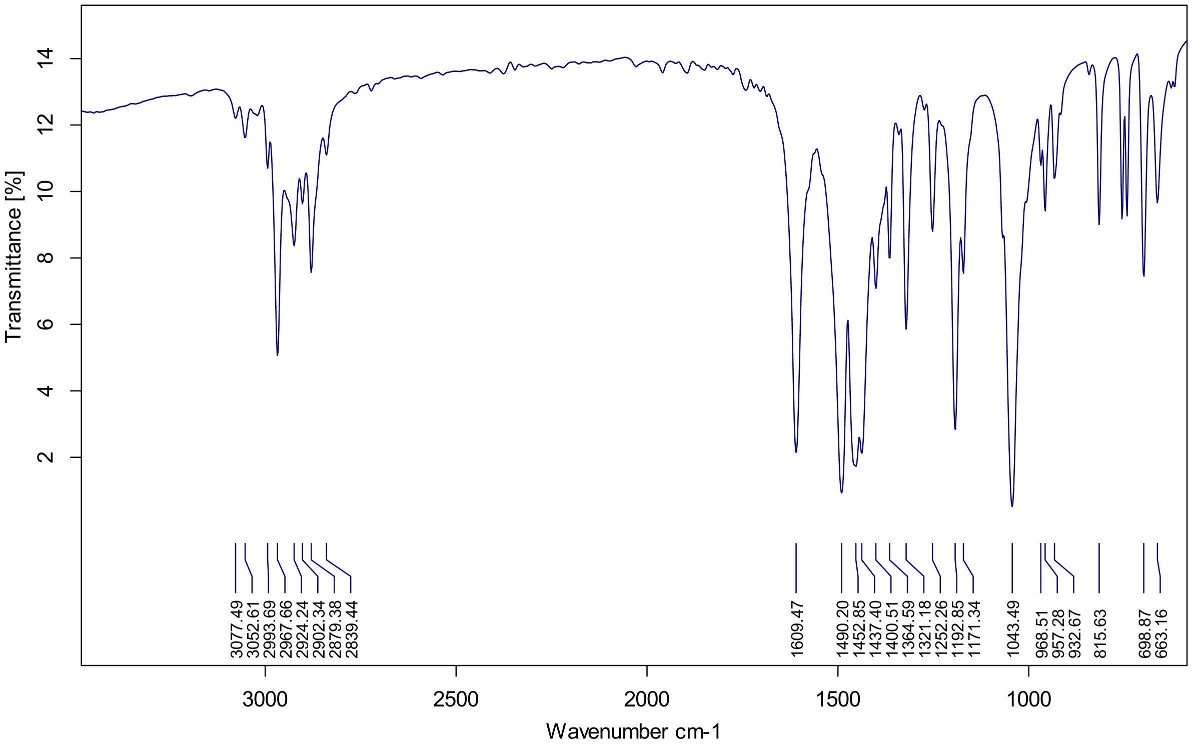

Figure S4. Infrared spectrum of $^{Ph}BOX^{Me2}ZnMe$ (**2a**; KBr).

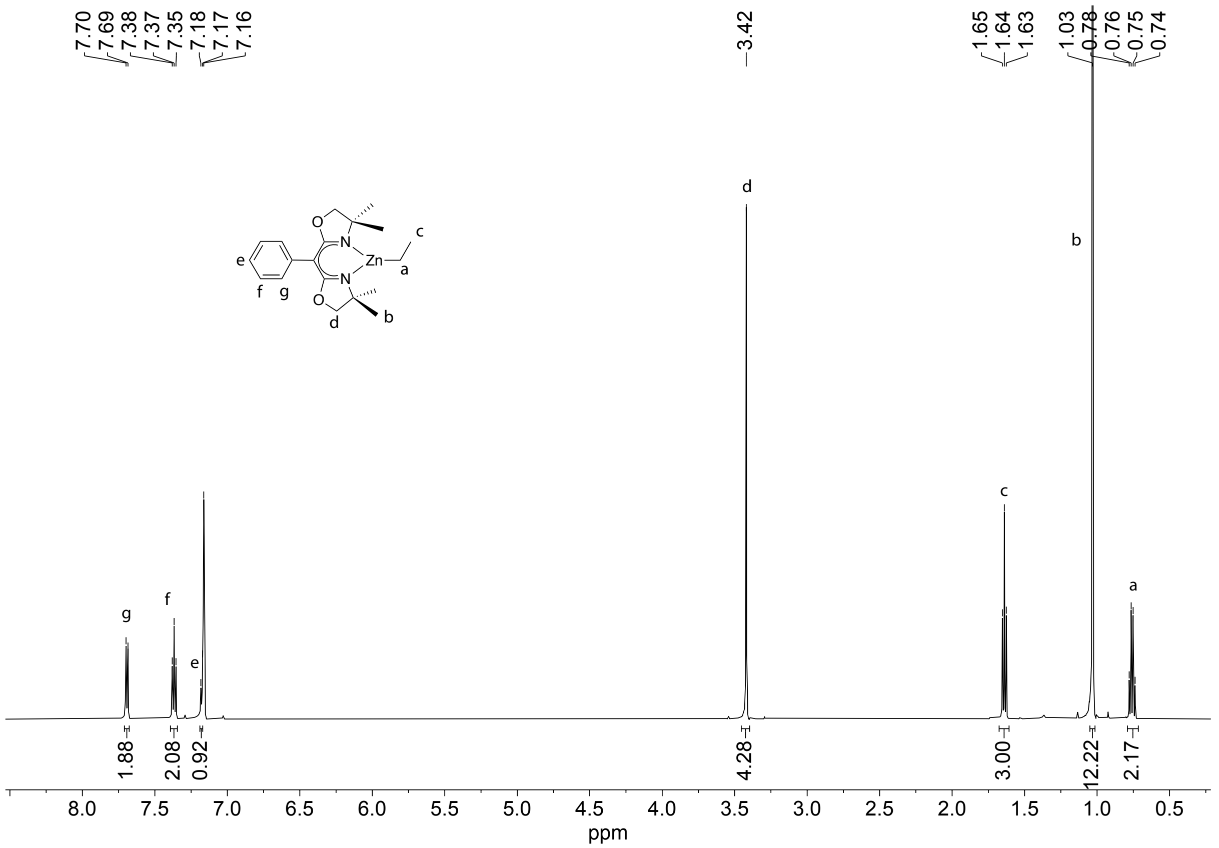

Figure S5. ^{1}H NMR spectrum of ^{Ph}BOX^{Me2}ZnEt (**2b**) in acquired in benzene-d_6.

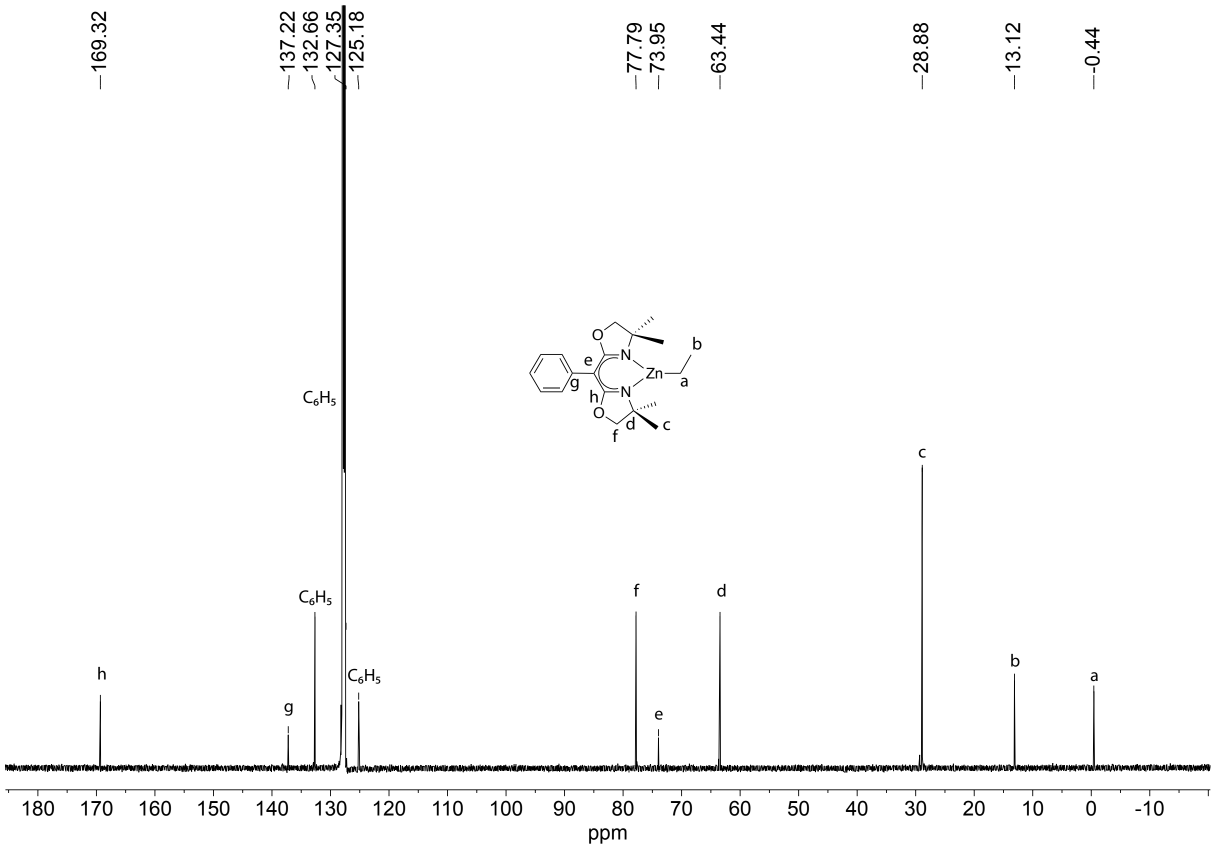

Figure S6. $^{13}C\{^{1}H\}$ NMR spectrum of $^{Ph}BOX^{Me2}ZnEt$ (**2b**) acquired in benzene-d_6.

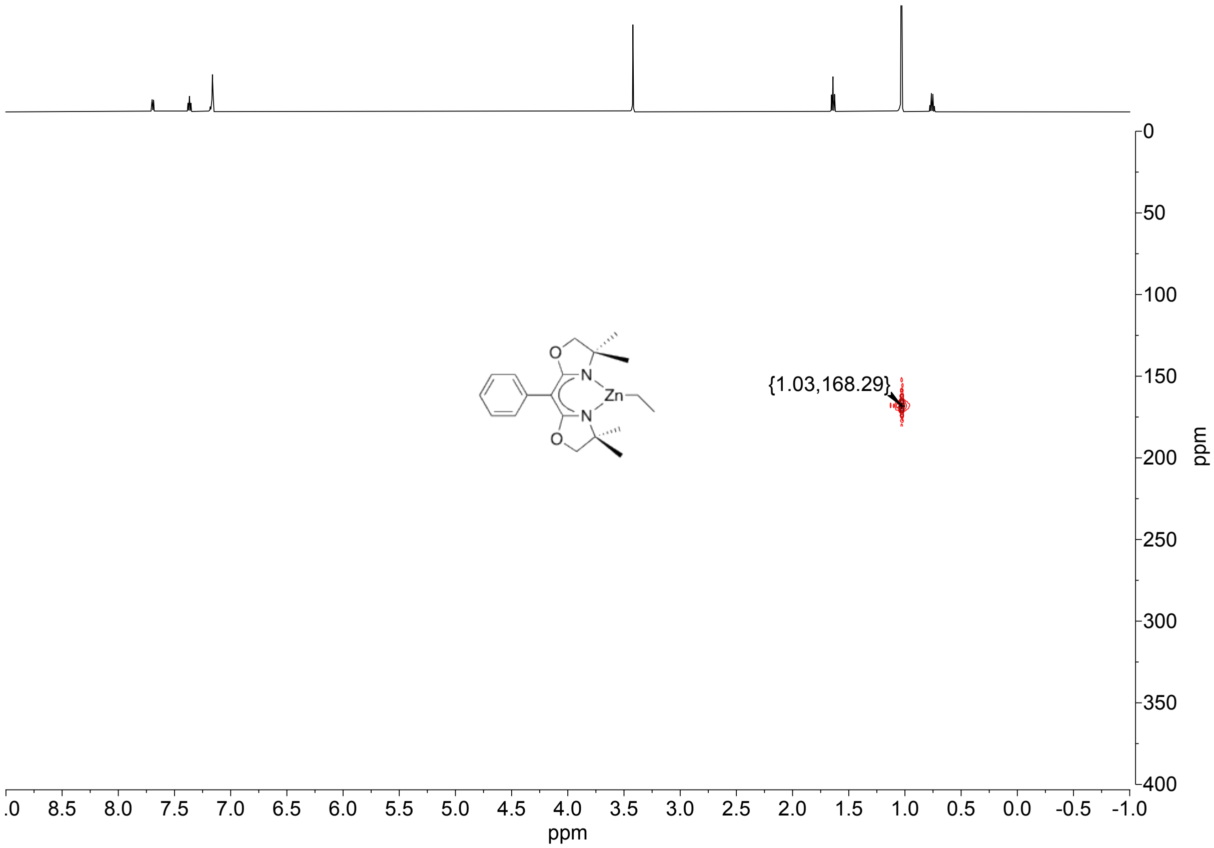

Figure S7. ^{15}N-^{1}H HMBC spectrum of ^{Ph}BOX^{Me2}ZnEt (**2b**) acquired in benzene-d_6. Recalibration to nitromethane as an external standard gives $\delta_N = -214$.

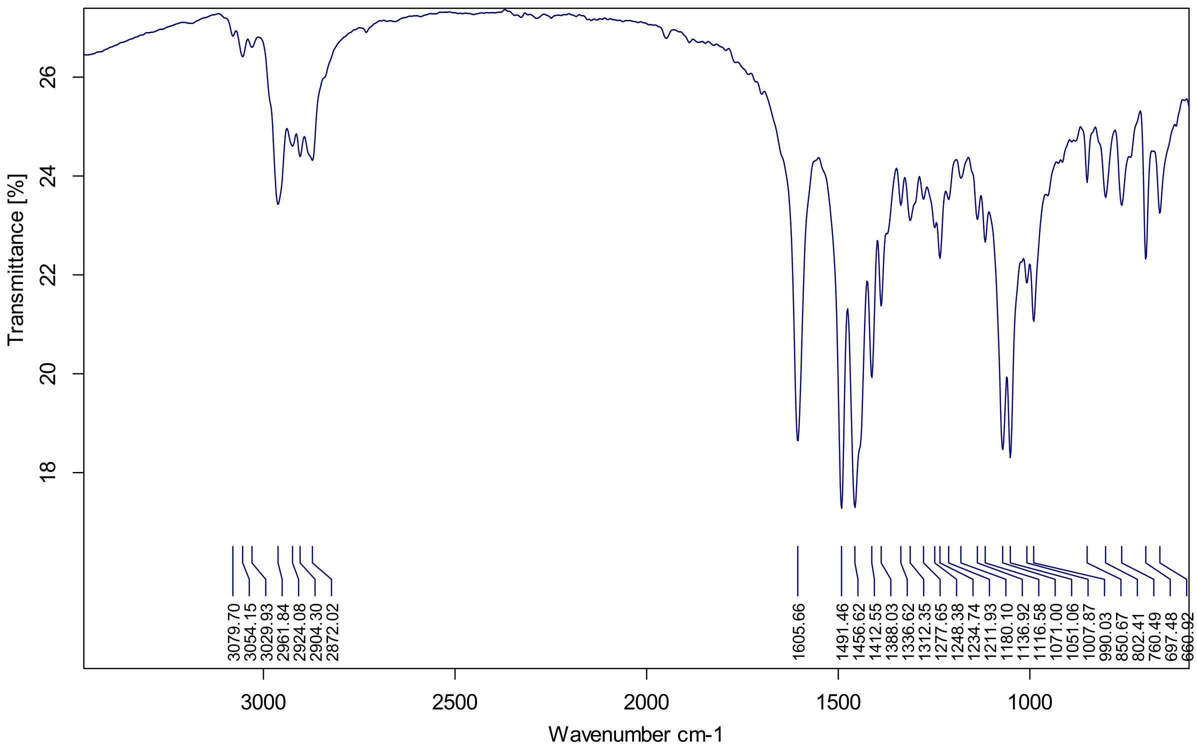

Figure S8. Infrared spectrum of ^{Ph}BOX^{Me2}ZnEt (**2b**, KBr).

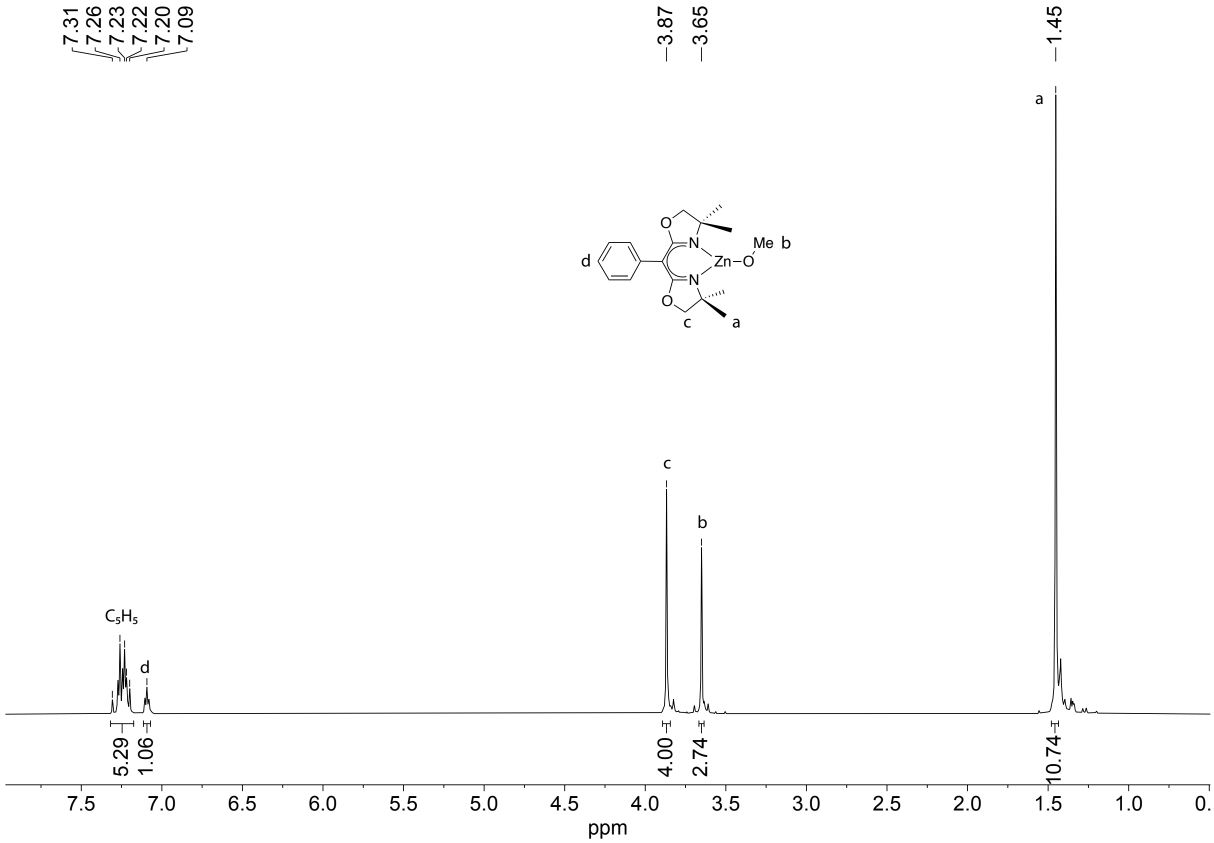

Figure S9. ^{1}H NMR spectrum of $^{Ph}BOX^{Me2}ZnOMe$ (**3a**) acquired in chloroform-*d*.

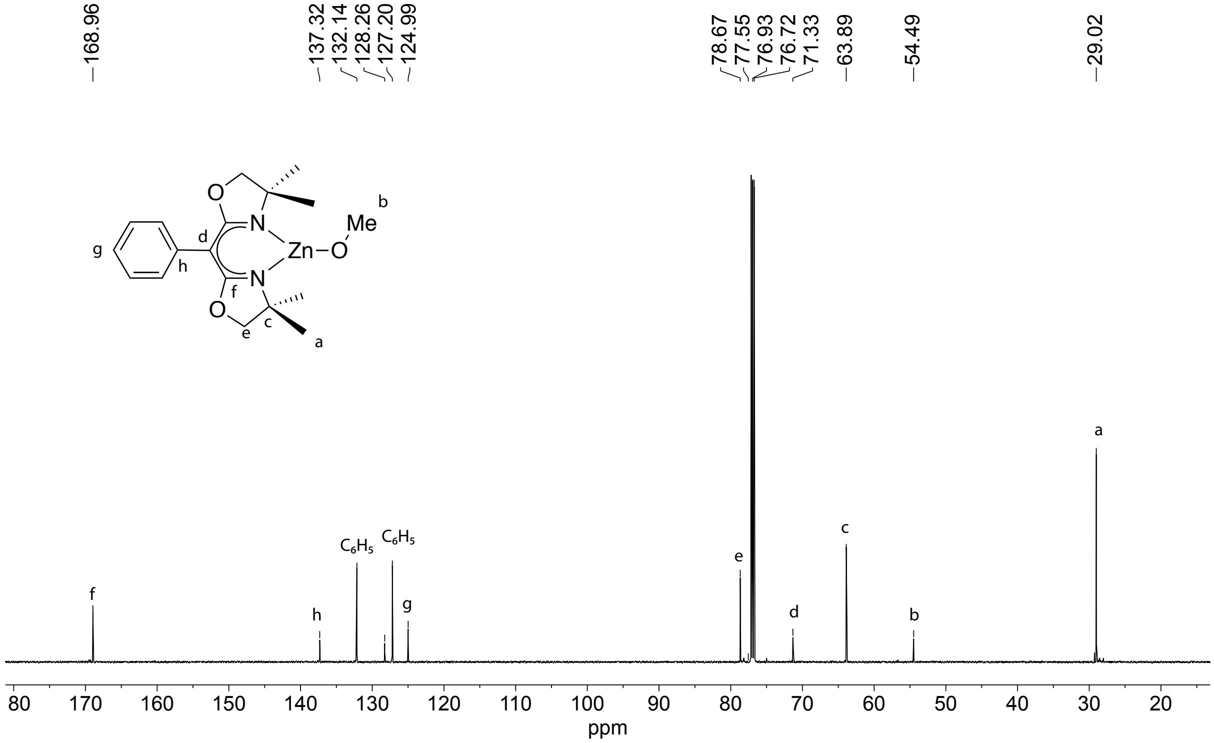

Figure S10. $^{13}C\{^1H\}$ NMR spectrum of $^{Ph}BOX^{Me2}ZnOMe$ (**3a**) acquired in chloroform-*d*.

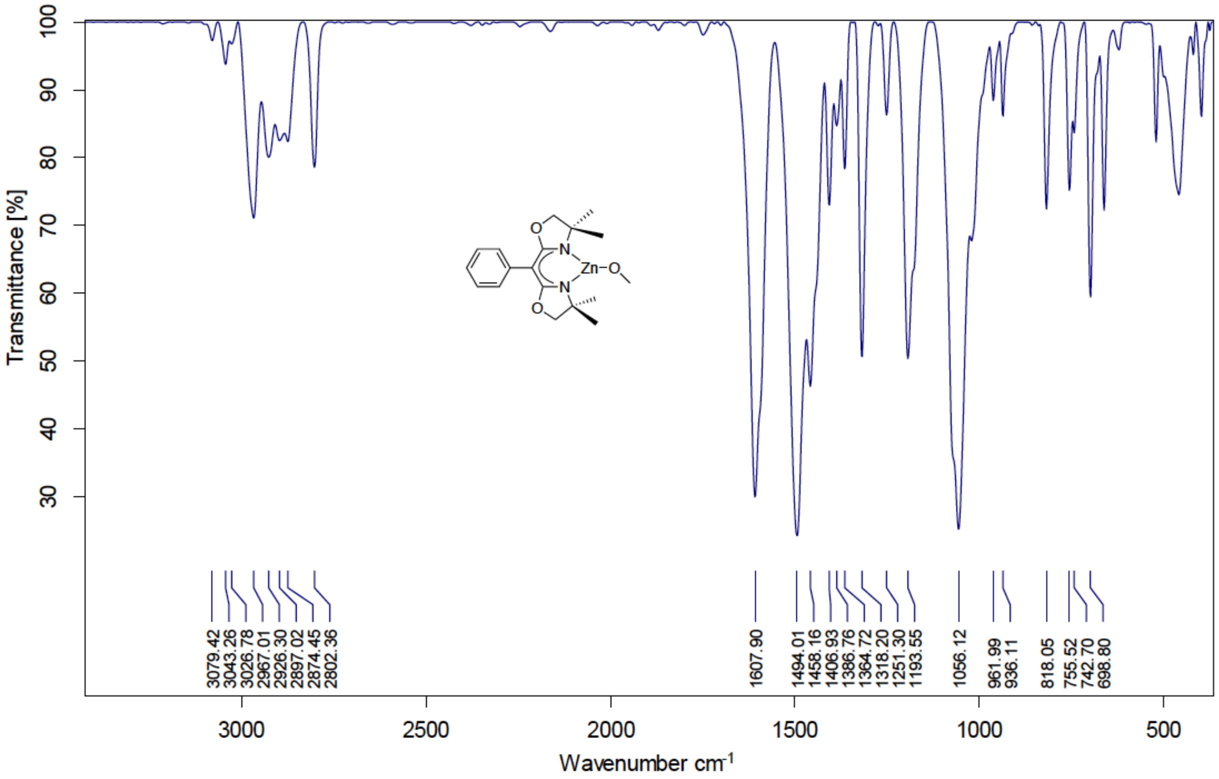

Figure S11. Infrared spectrum of $^{Ph}BOX^{Me2}ZnOMe$ (**3a**, KBr).

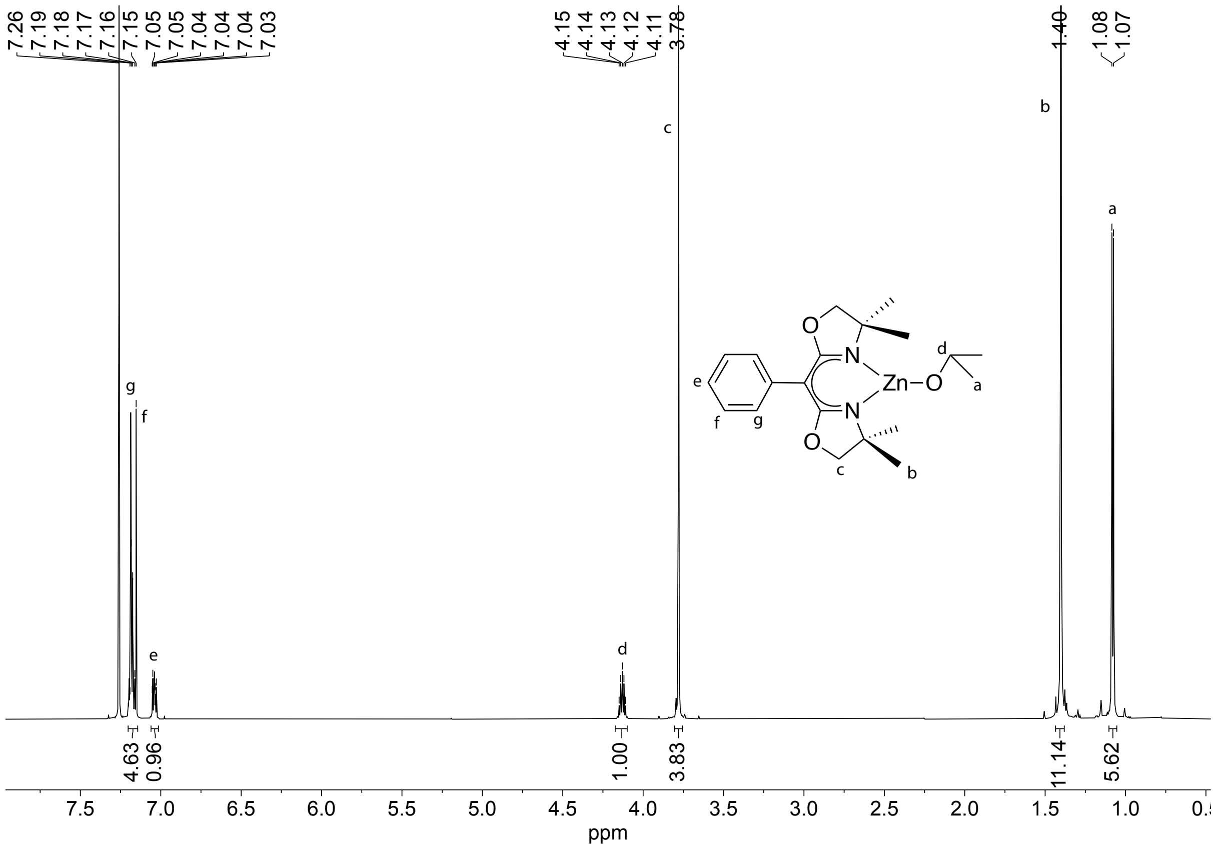

Figure S12. ^{1}H NMR spectrum of $^{Ph}BOX^{Me2}ZnO\textit{i}Pr$ (**3b**) acquired in chloroform-*d*.

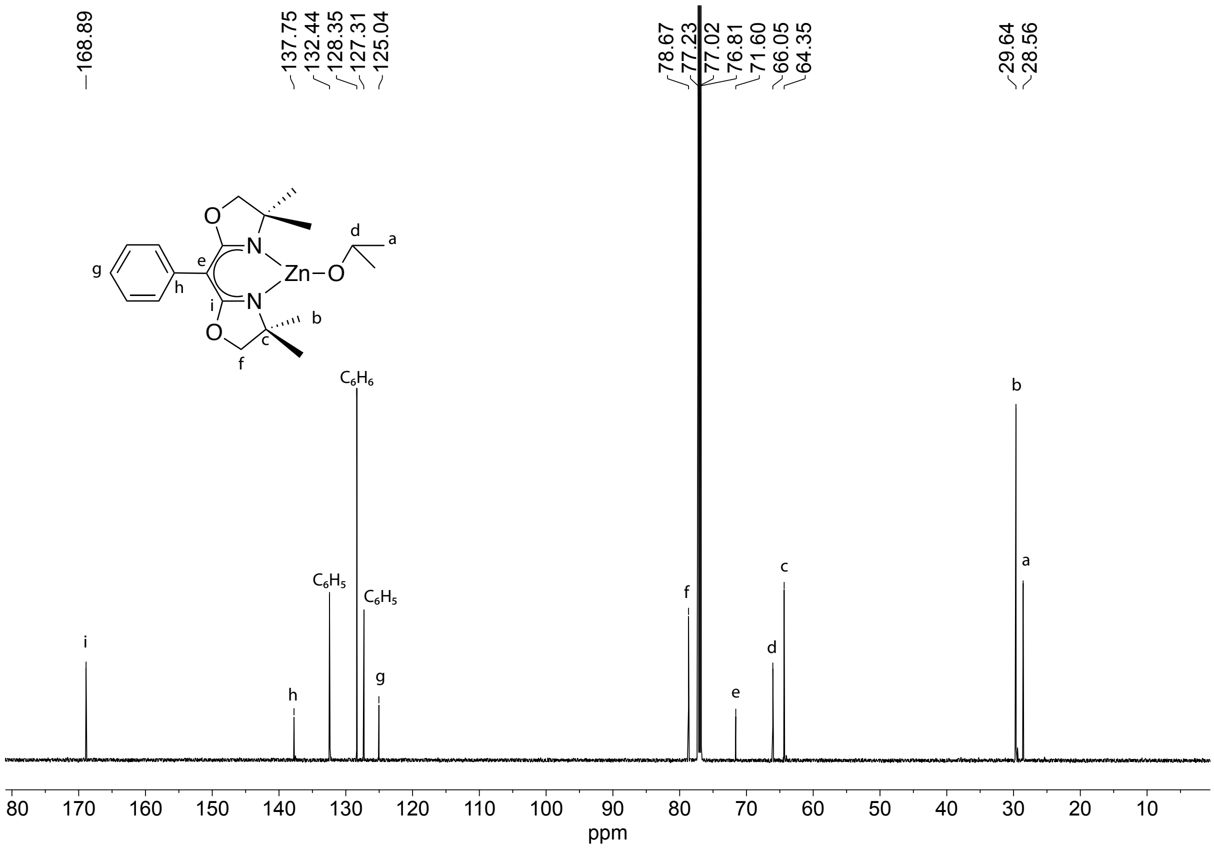

Figure S13. $^{13}C\{^1H\}$ NMR spectrum of $^{Ph}BOX^{Me2}ZnO\textit{i}Pr$ (**3b**) acquired in chloroform-*d*.

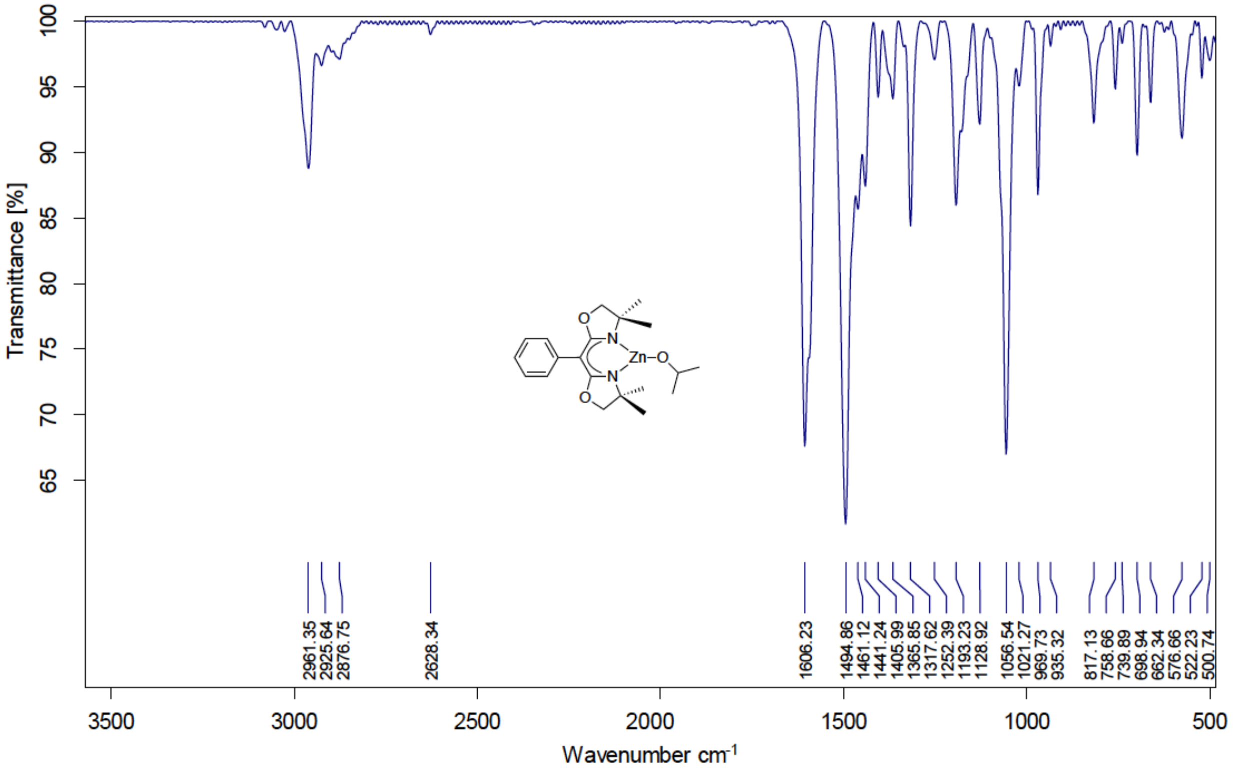

Figure S14. Infrared spectrum of ^{Ph}BOX^{Me2}ZnO*i*Pr (**3b**, KBr).

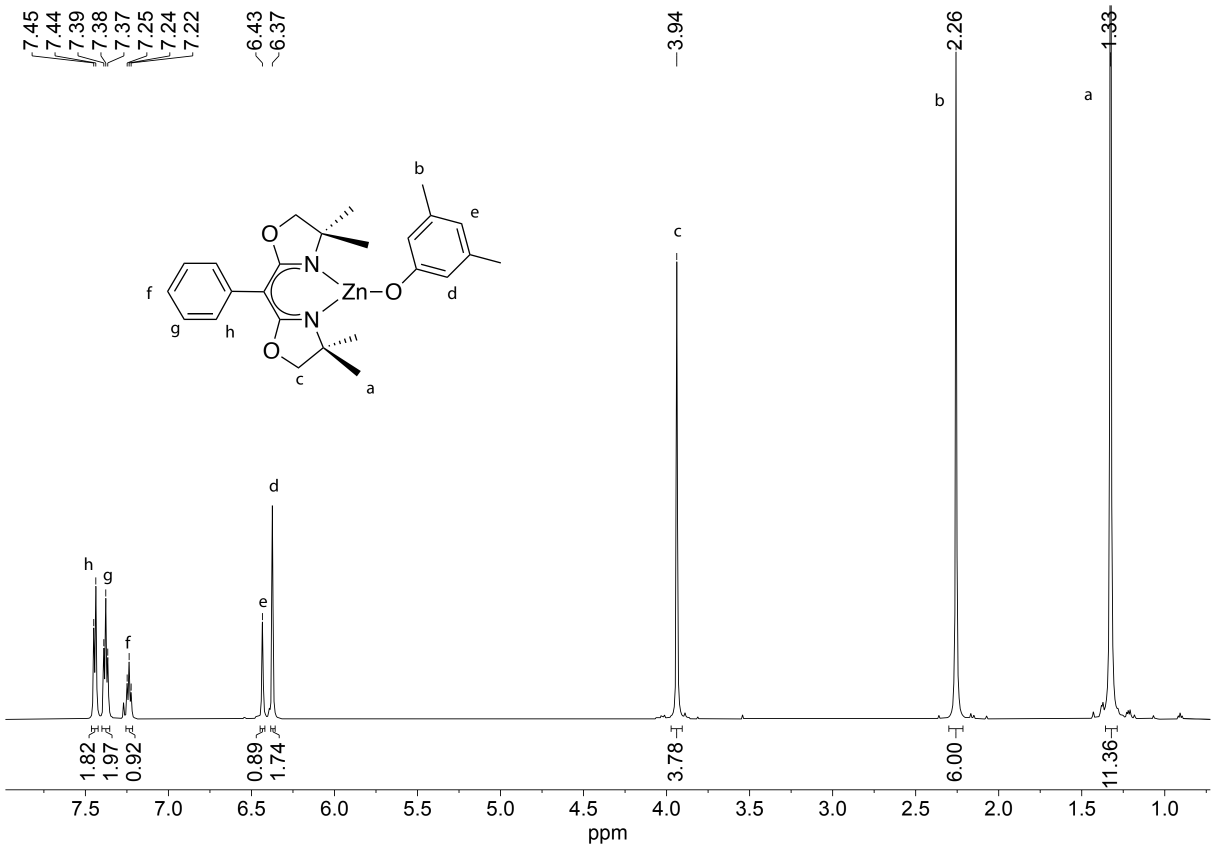

Figure S15. ^{1}H NMR spectrum of $^{Ph}BOX^{Me2}ZnOC_6H_3Me_2$ (**3c**) acquired in chloroform-*d*.

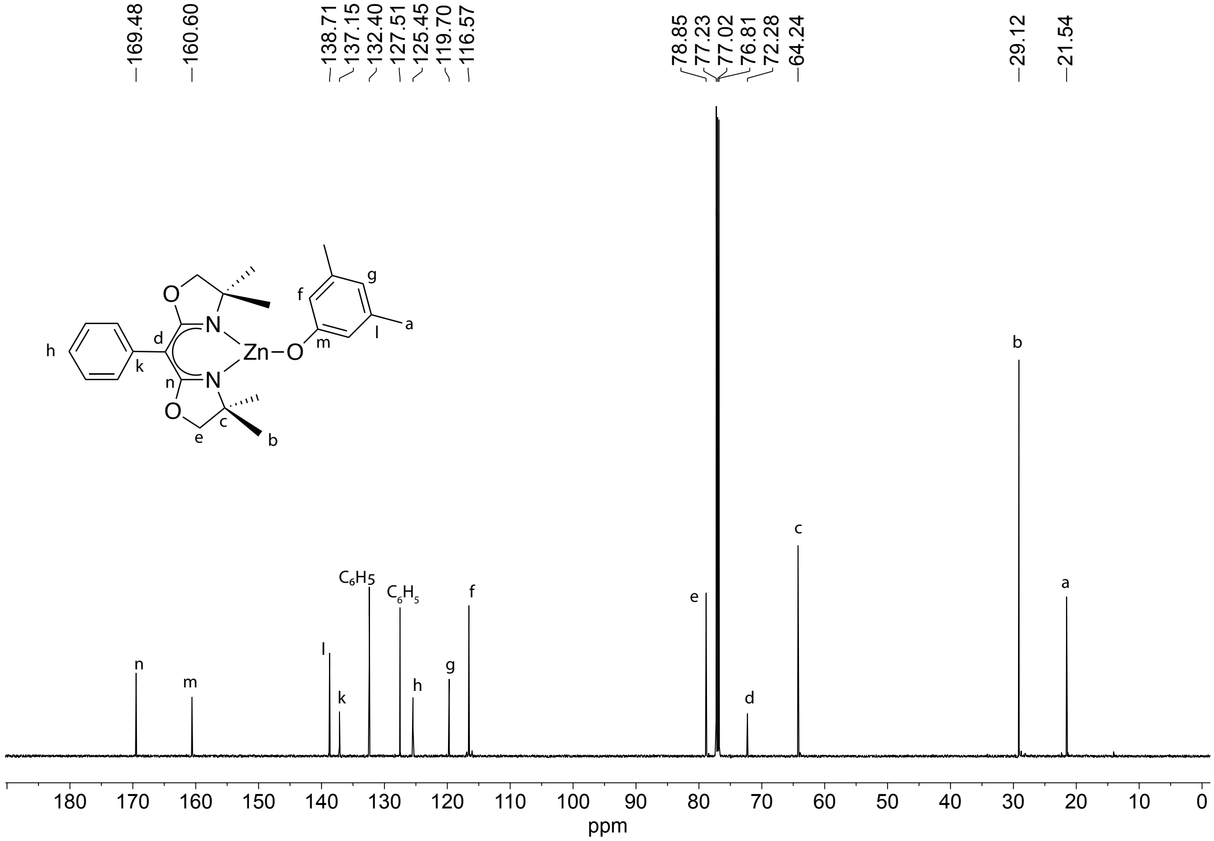

Figure S16. $^{13}C\{^1H\}$ NMR spectrum of $^{Ph}BOX^{Me2}ZnOC_6H_3Me_2$ (**3c**) acquired in chloroform-*d*.

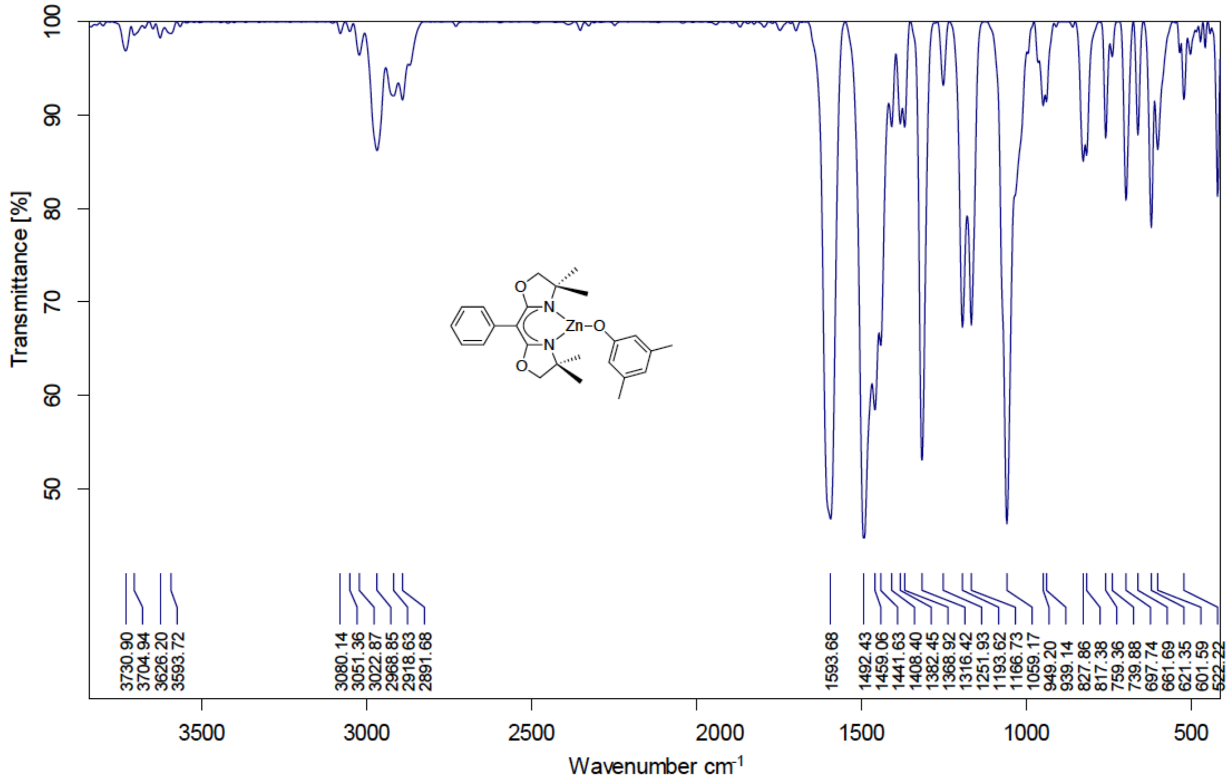

Figure S17. Infrared spectrum of $^{Ph}BOX^{Me2}ZnOC_6H_3Me_2$ (**3c**, KBr).

NMR Spectra of Silane Products

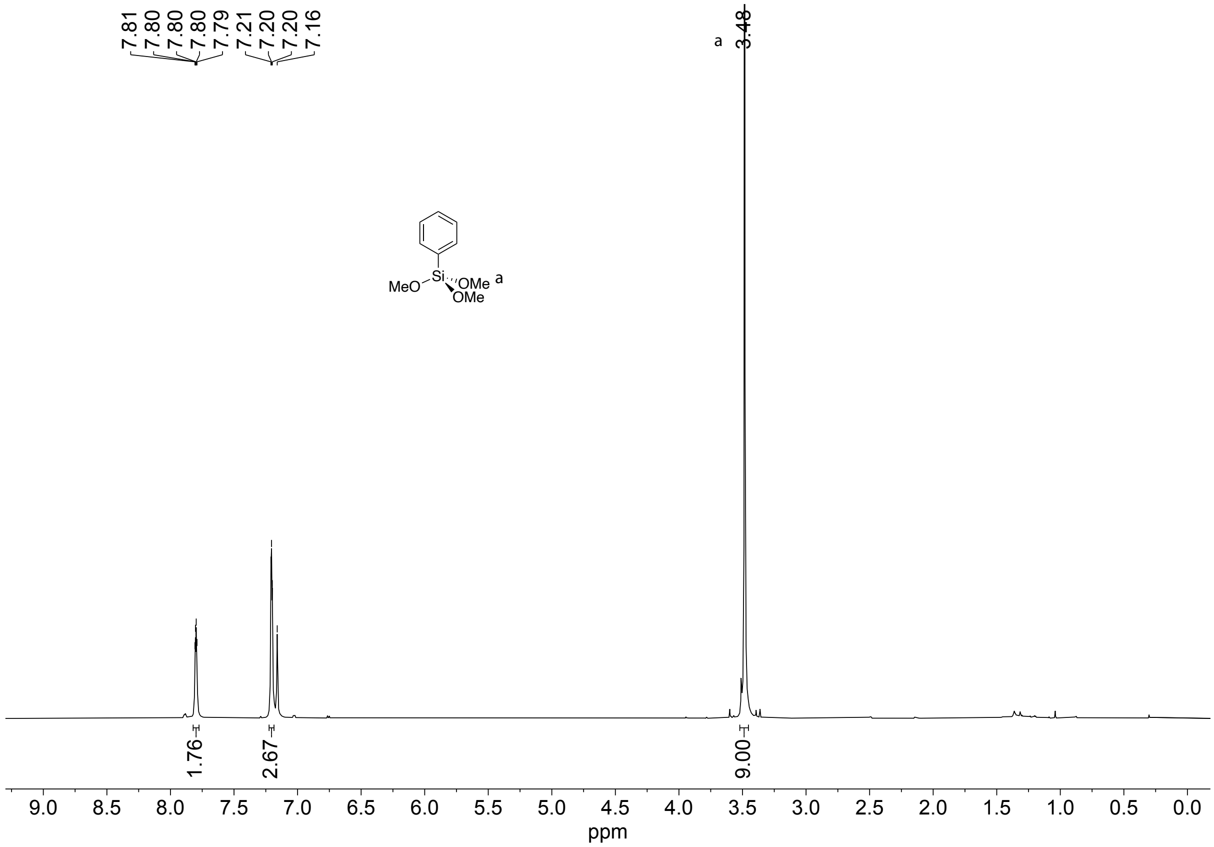

Figure S18. ^{1}H NMR spectrum of $PhSi(OMe)_3$ acquired in benzene-d_6 obtained from ^{Ph}**BOX**^{Me2}**ZnMe**-catalyzed dehydrocoupling of phenyl silane and methanol.

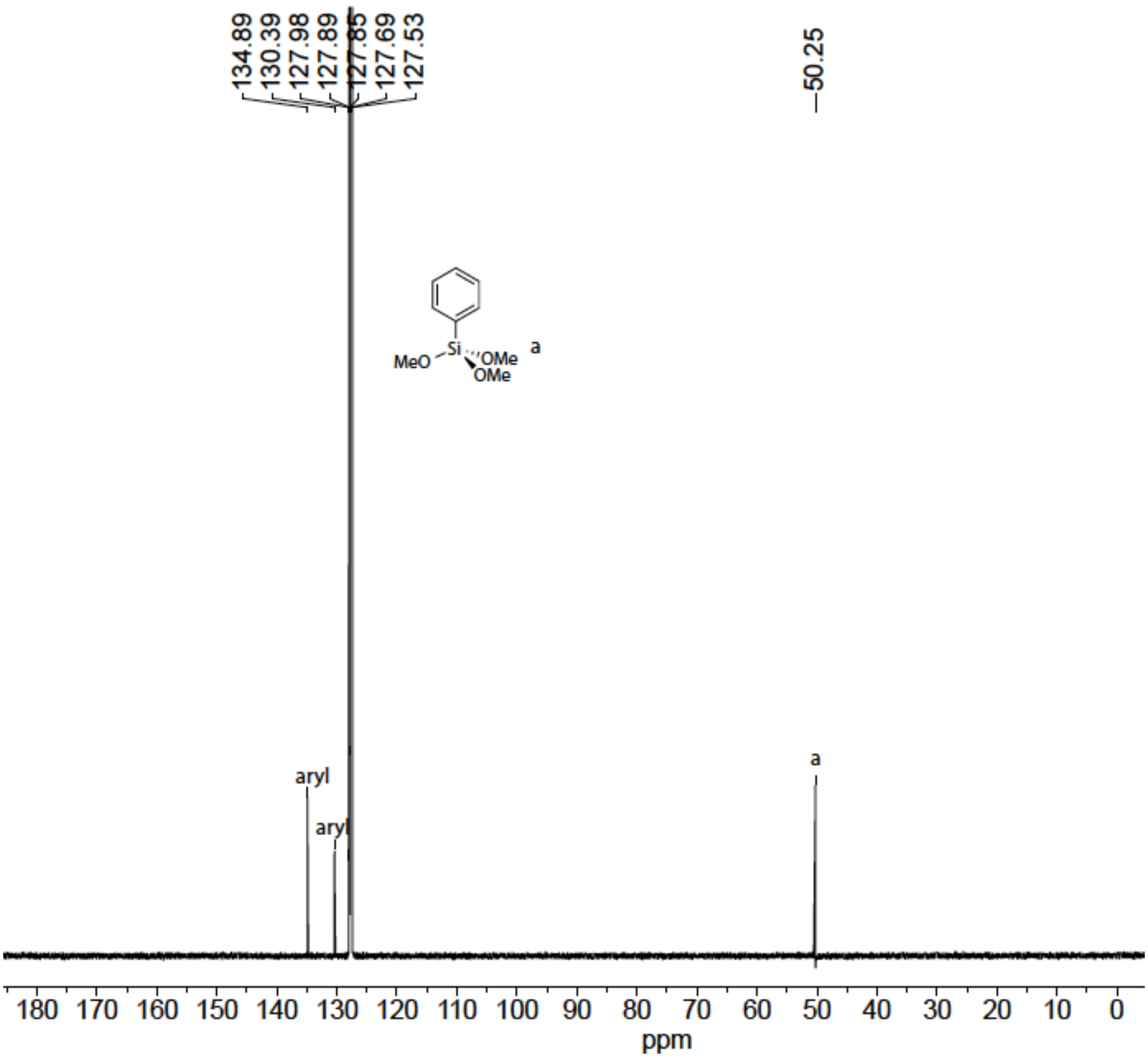

Figure S19. $^{13}C\{^1H\}$ NMR spectrum of $PhSi(OMe)_3$ acquired in benzene-d_6, obtained from ^{Ph}**BOX**^{Me2}**ZnMe**-catalyzed dehydrocoupling of phenyl silane and methanol.

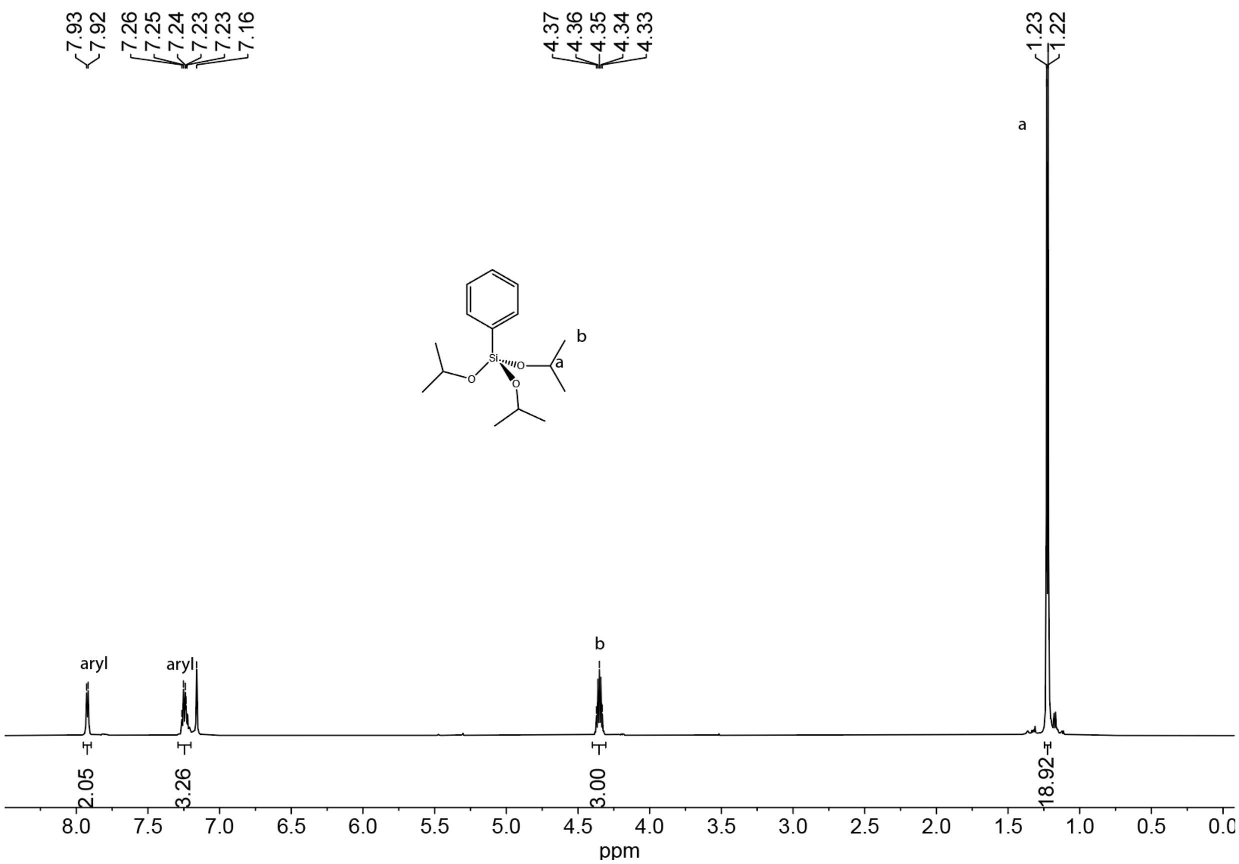

Figure S20. ^{1}H NMR spectrum of PhSi(O*i*Pr)$_3$ acquired in benzene-d_6, obtained from **^{Ph}BOX^{Me2}ZnMe**-catalyzed dehydrocoupling of phenyl silane and isopropanol.

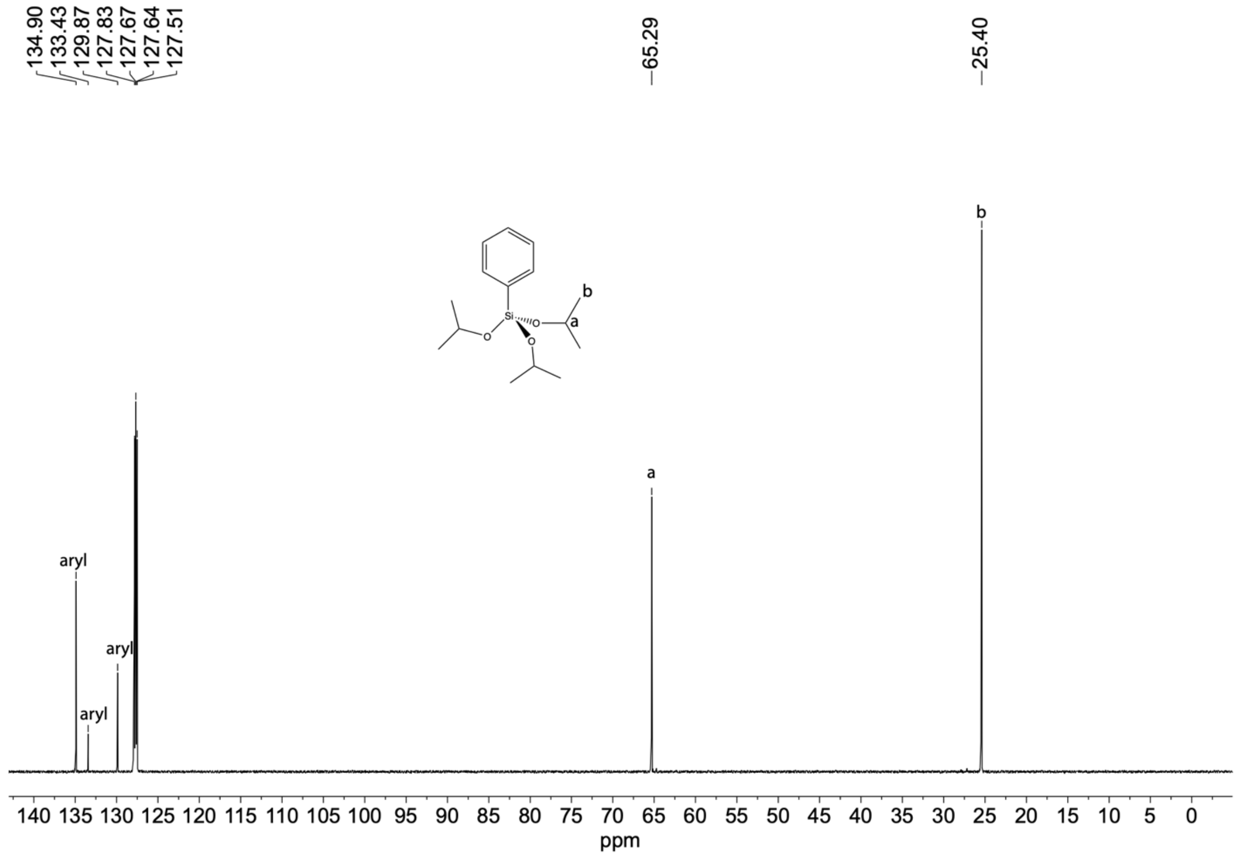

Figure S21. $^{13}C\{^{1}H\}$ NMR spectrum of PhSi(O*i*Pr)$_3$ acquired in benzene-d_6, obtained from **^{Ph}BOX^{Me2}ZnMe**-catalyzed dehydrocoupling of phenyl silane and isopropanol.

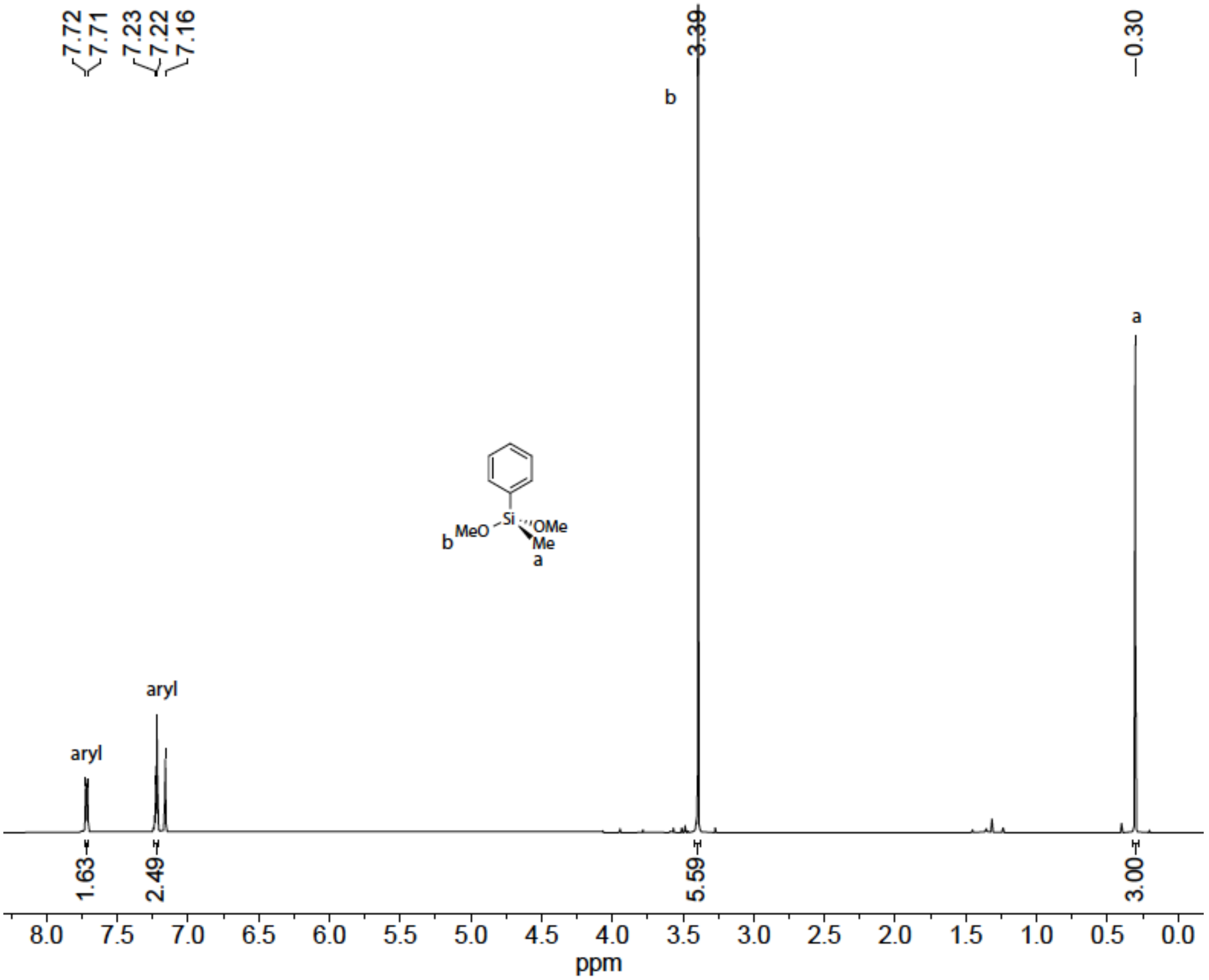

Figure S22. ^{1}H NMR spectrum of PhMeSi(OMe)$_2$ acquired in benzene-d_6, obtained from **^{Ph}BOX^{Me2}ZnMe**-catalyzed dehydrocoupling of phenyl methyl silane and methanol.

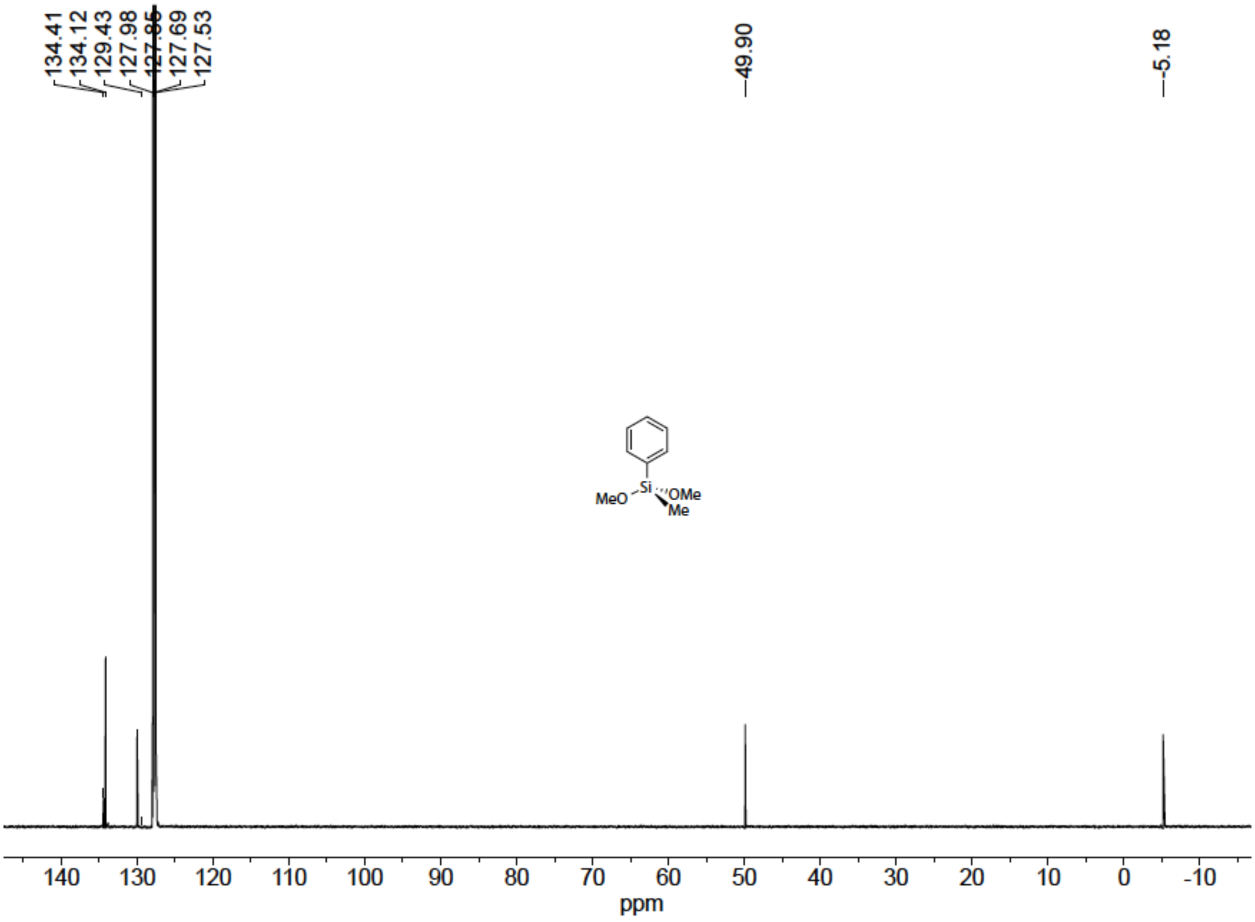

Figure S23. $^{13}C\{^1H\}$ NMR spectrum of $PhMeSi(OMe)_2$ acquired in benzene-d_6, obtained from **$^{Ph}BOX^{Me2}ZnMe$**-catalyzed dehydrocoupling of phenyl methyl silane and methanol.

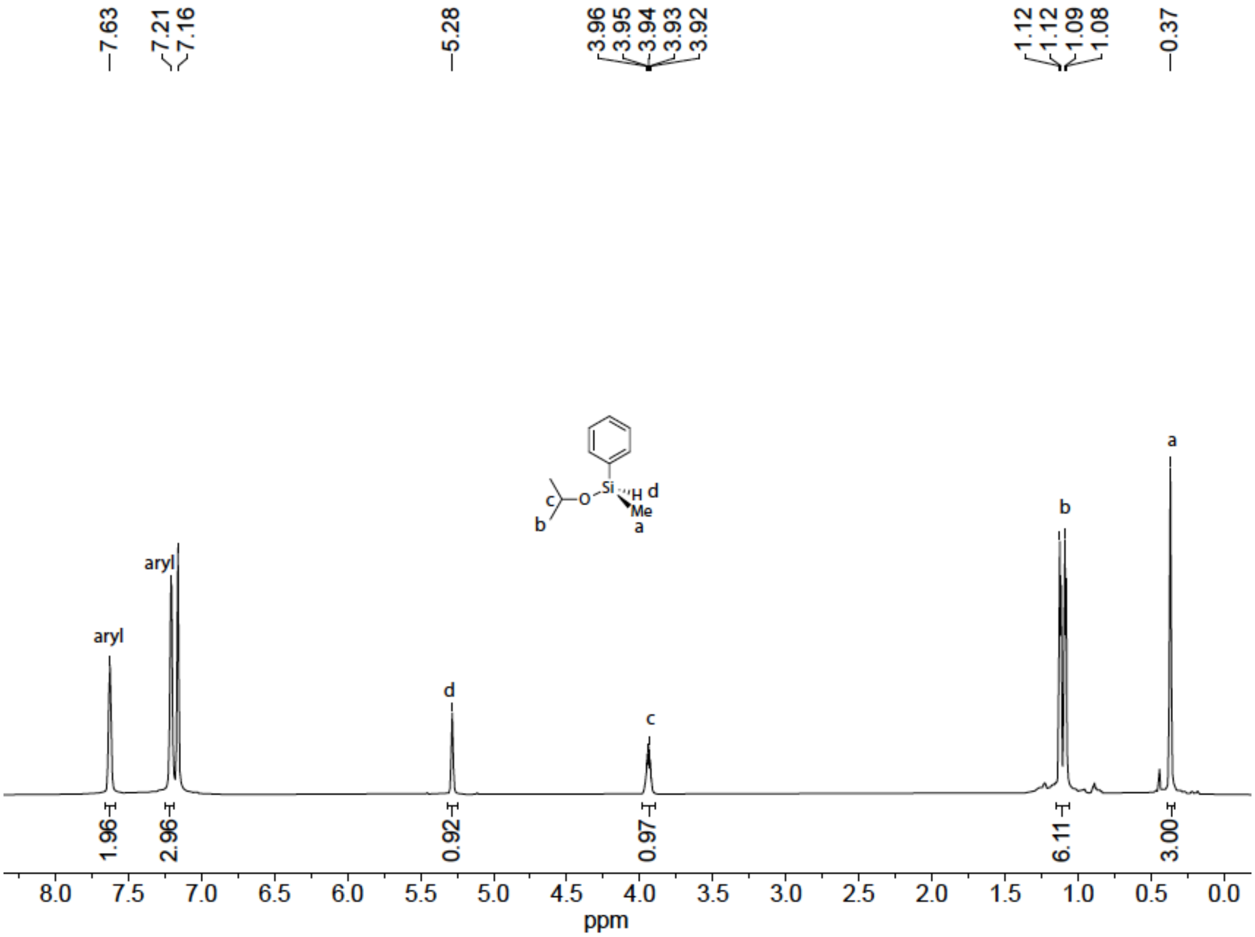

Figure S24. ^{1}H NMR spectrum of PhMeHSiO*i*Pr acquired in benzene-d_6, obtained from ^{Ph}**BOX^{Me2}ZnMe**-catalyzed dehydrocoupling of phenyl methyl silane and isopropanol.

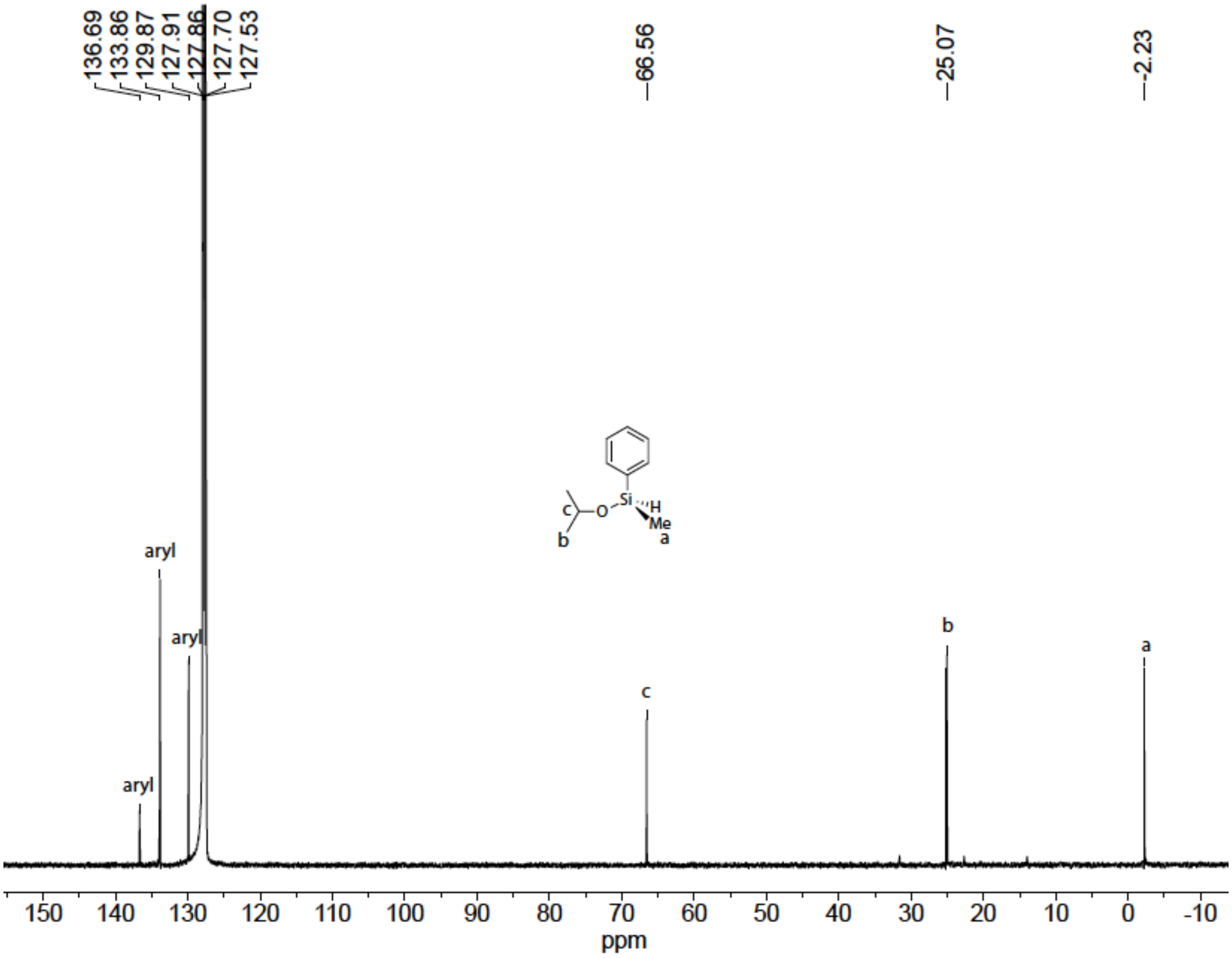

Figure S25. $^{13}C\{^{1}H\}$ NMR spectrum of PhMeHSiO*i*Pr acquired in benzene-d_6, obtained from $^{\mathbf{Ph}}$**BOX**$^{\mathbf{Me2}}$**ZnMe**-catalyzed dehydrocoupling of phenyl methyl silane and isopropanol.

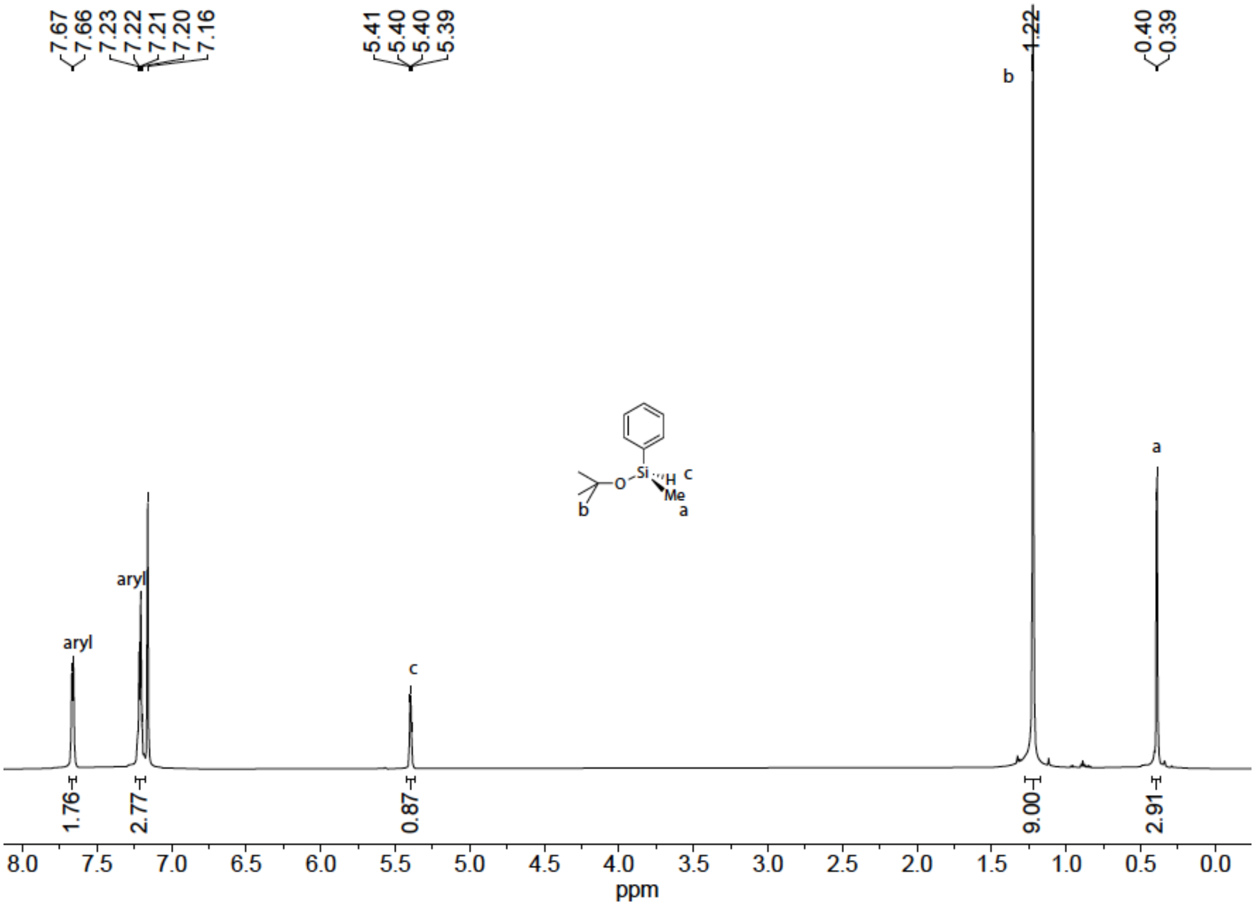

Figure S26. ^{1}H NMR spectrum of PhMeHSiO*t*Bu acquired in benzene-d_6, obtained from ^{Ph}**BOX**$^{\mathbf{Me2}}$**ZnMe**-catalyzed dehydrocoupling of phenyl methyl silane and tert-butanol.

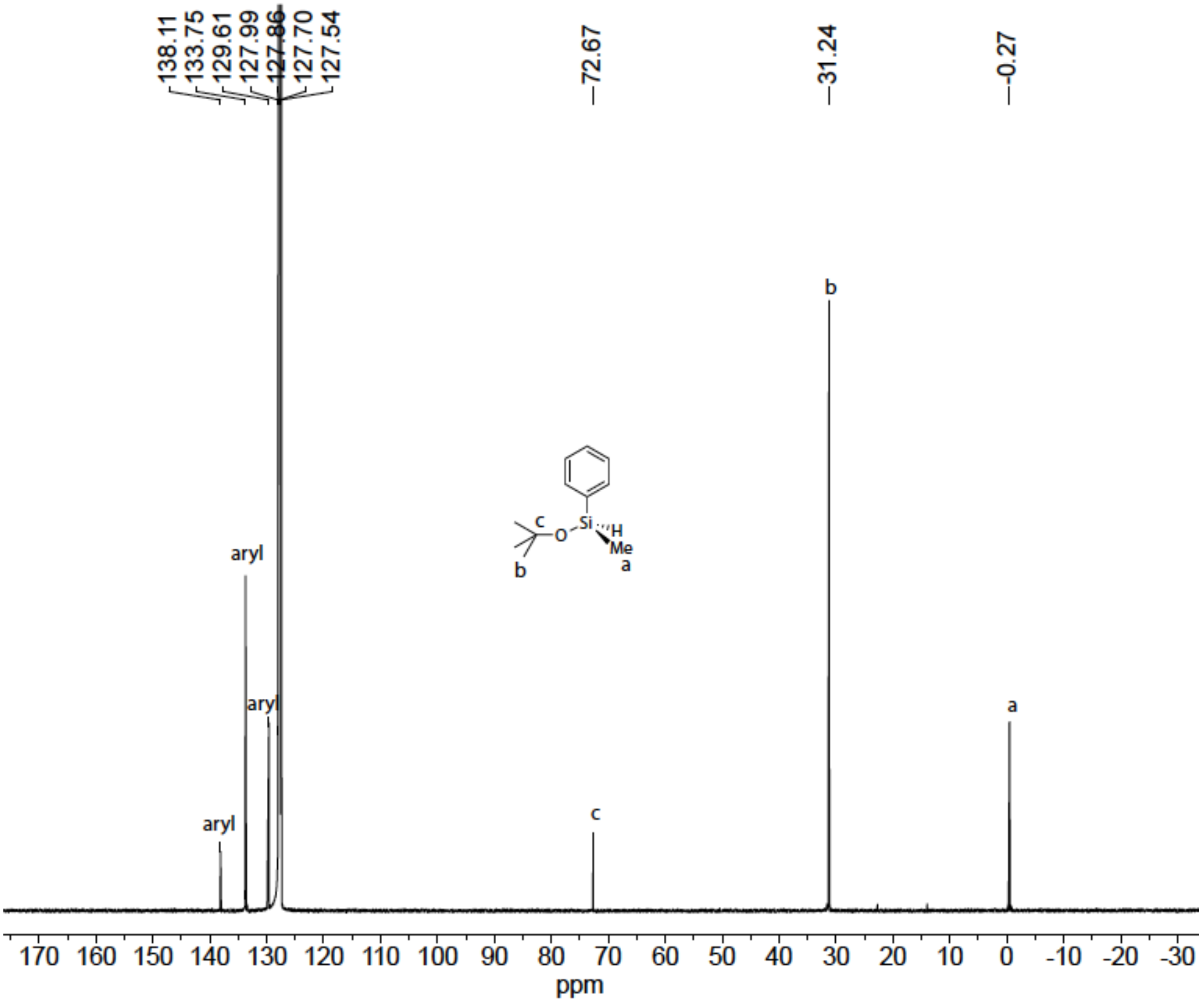

Figure S27. $^{13}C\{^1H\}$ NMR spectrum of PhMeHSiO*t*Bu acquired in benzene-d_6, obtained from ^{Ph}**BOX**^{Me2}**ZnMe**-catalyzed dehydrocoupling of phenyl methyl silane and tert-butanol.

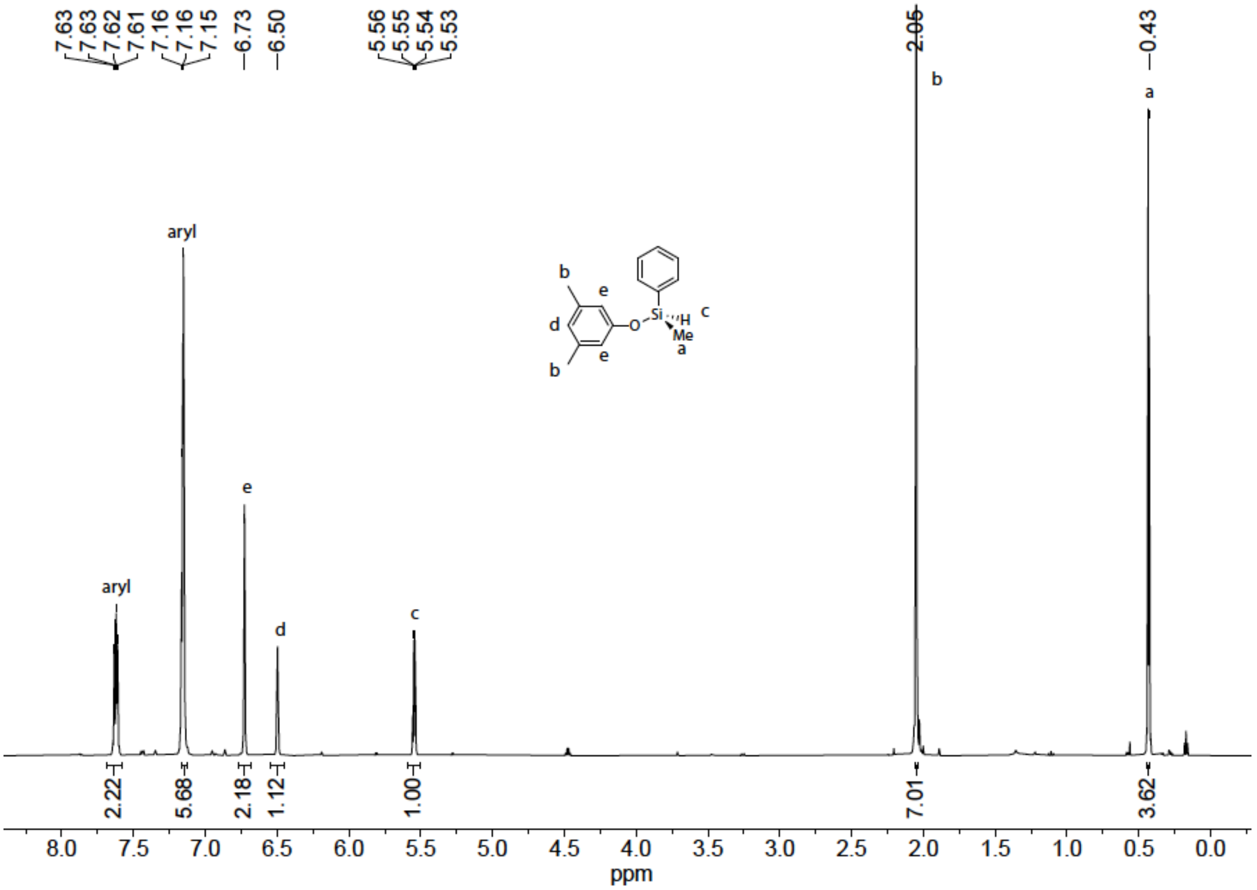

Figure S28. ^{1}H NMR spectrum of $PhMeHSiOC_6H_3Me_2$ acquired in benzene-d_6, obtained from **$^{Ph}BOX^{Me2}ZnMe$**-catalyzed dehydrocoupling of phenyl methyl silane and 2,5-dimethylphenol.

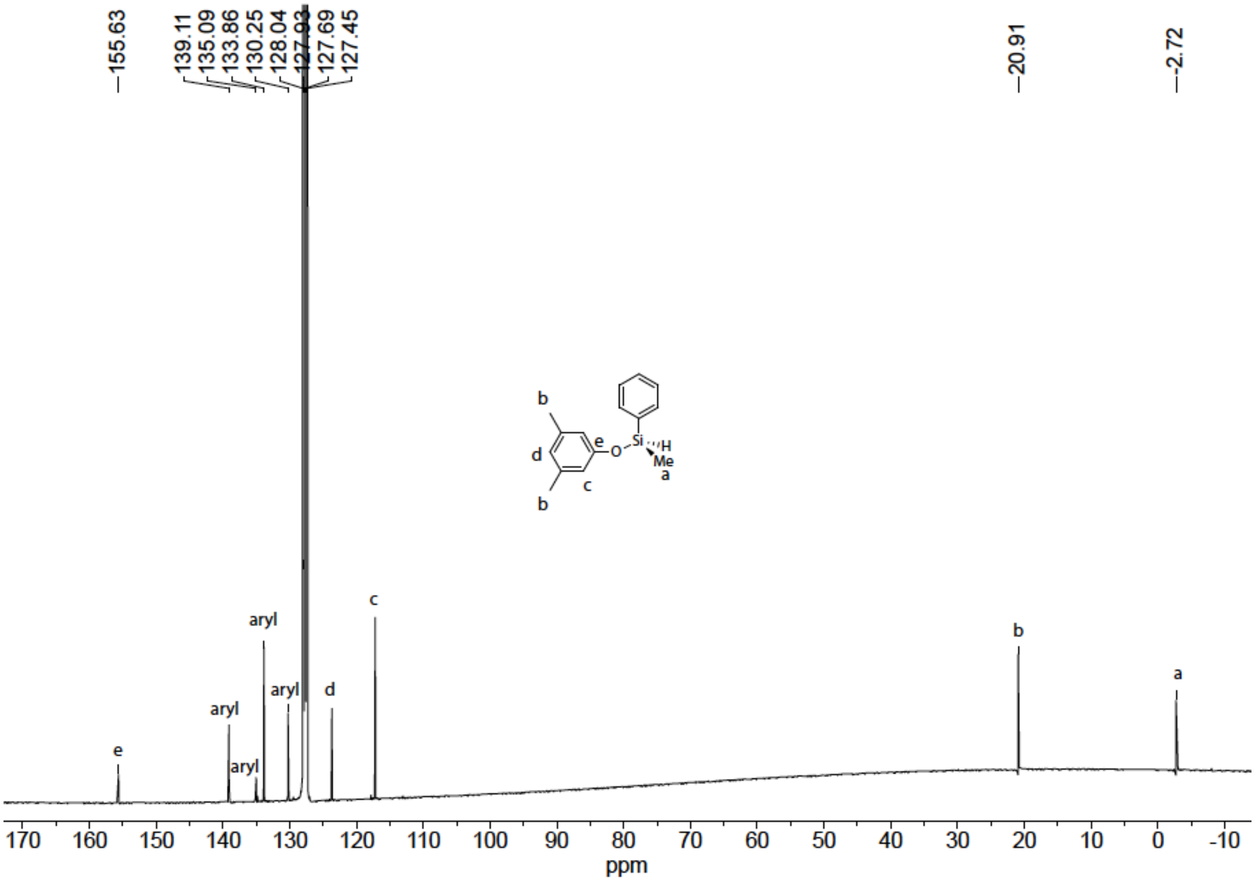

Figure S29. $^{13}C\{^{1}H\}$ NMR spectrum of $PhMeHSiOC_6H_3Me_2$ acquired in benzene-d_6, obtained from ^{Ph}**BOX**^{Me2}**ZnMe**-catalyzed dehydrocoupling of phenyl methyl silane and 2,5-dimethylphenol.

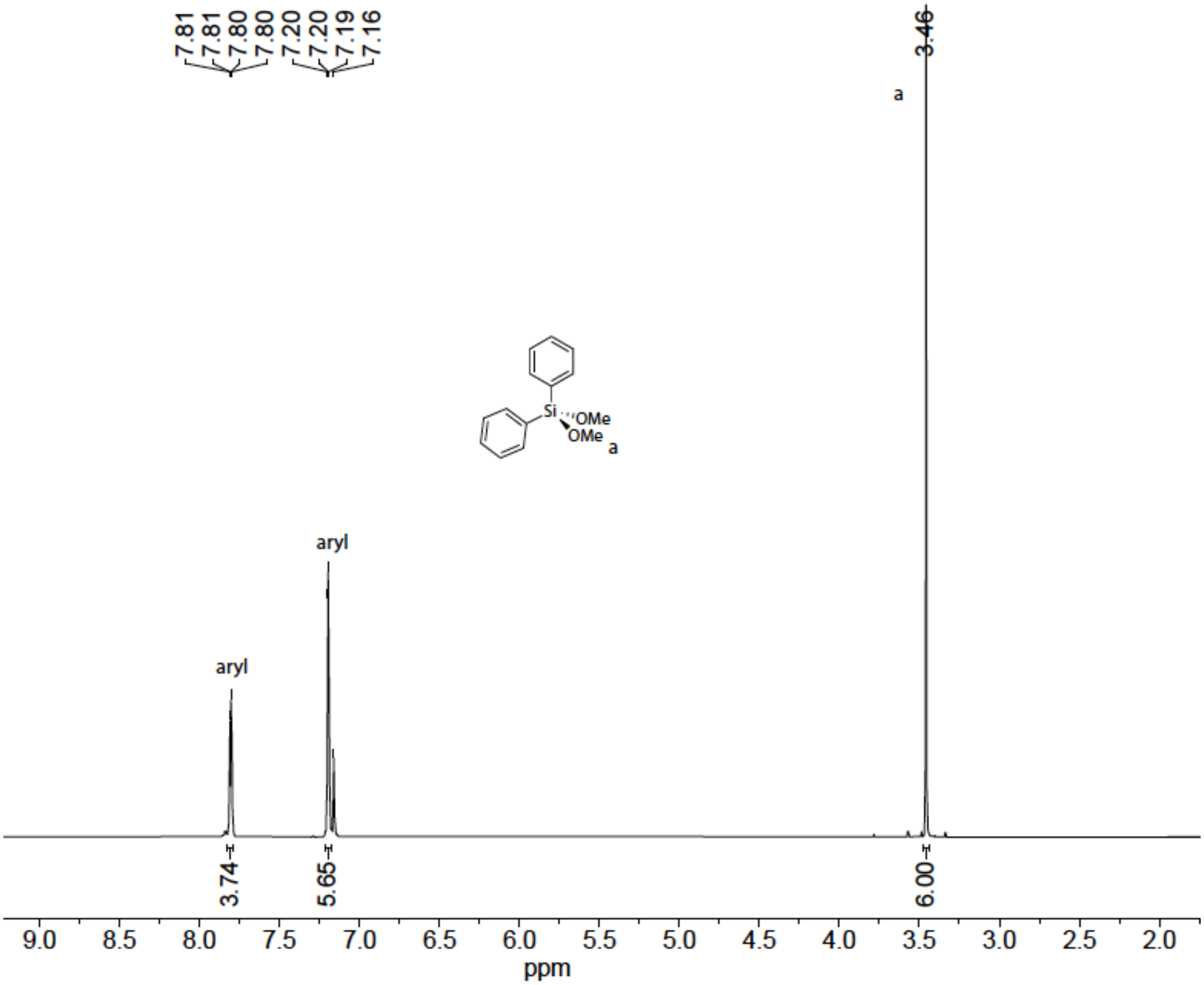

Figure S30. ^{1}H NMR spectrum of $Ph_2Si(OMe)_2$ acquired in benzene-d_6, obtained from **^{Ph}BOX^{Me2}ZnMe**-catalyzed dehydrocoupling of diphenyl silane and methanol.

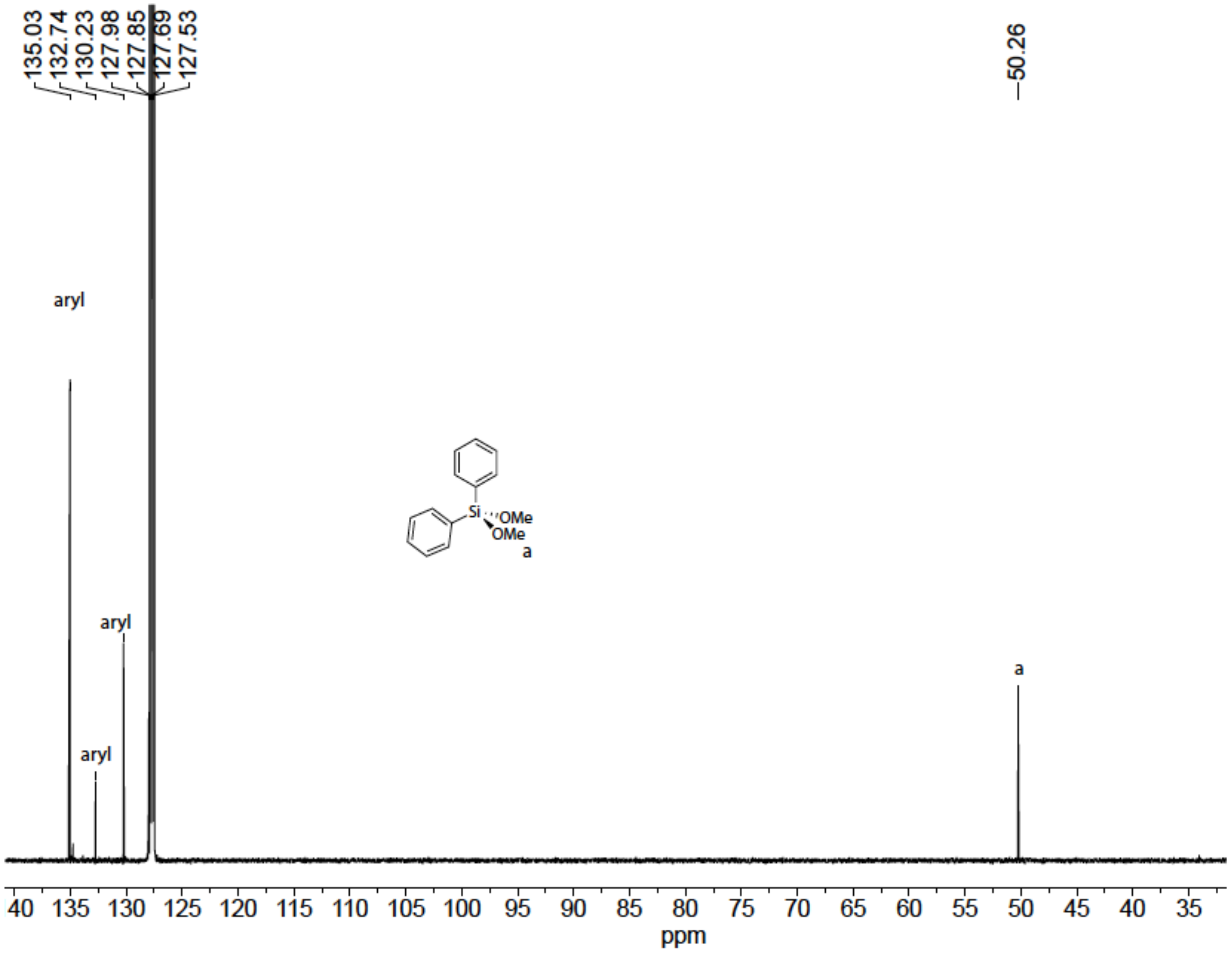

Figure S31. $^{13}C\{^{1}H\}$ NMR spectrum of $Ph_2Si(OMe)_2$ acquired in benzene-d_6, obtained from **^{Ph}BOX^{Me2}ZnMe**-catalyzed dehydrocoupling of diphenylsilane and methanol.

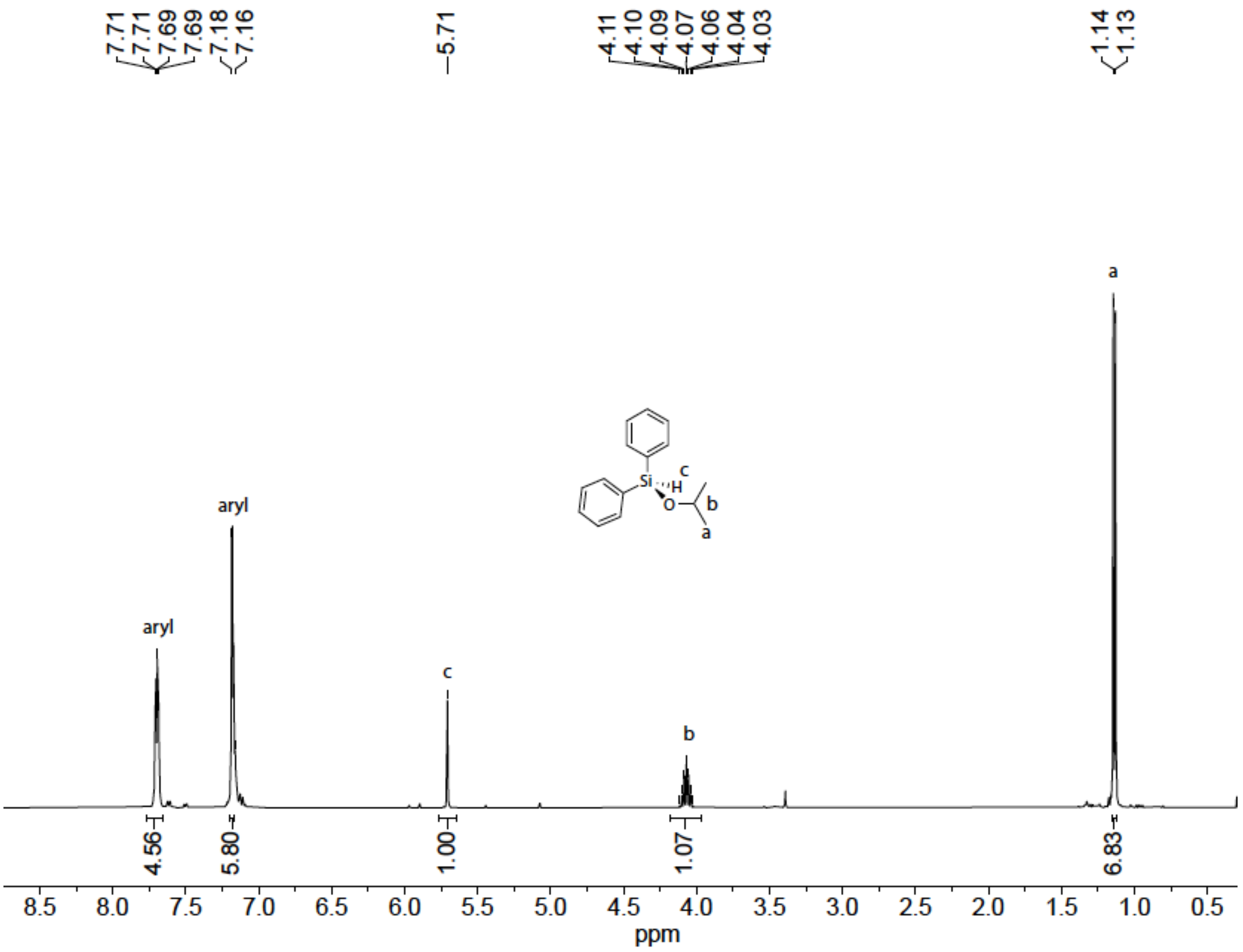

Figure S32. ^{1}H NMR spectrum of Ph_2HSiO*i*Pr acquired in benzene-d_6, obtained from **^{Ph}BOX^{Me2}ZnMe**-catalyzed dehydrocoupling of diphenylsilane and isopropanol.

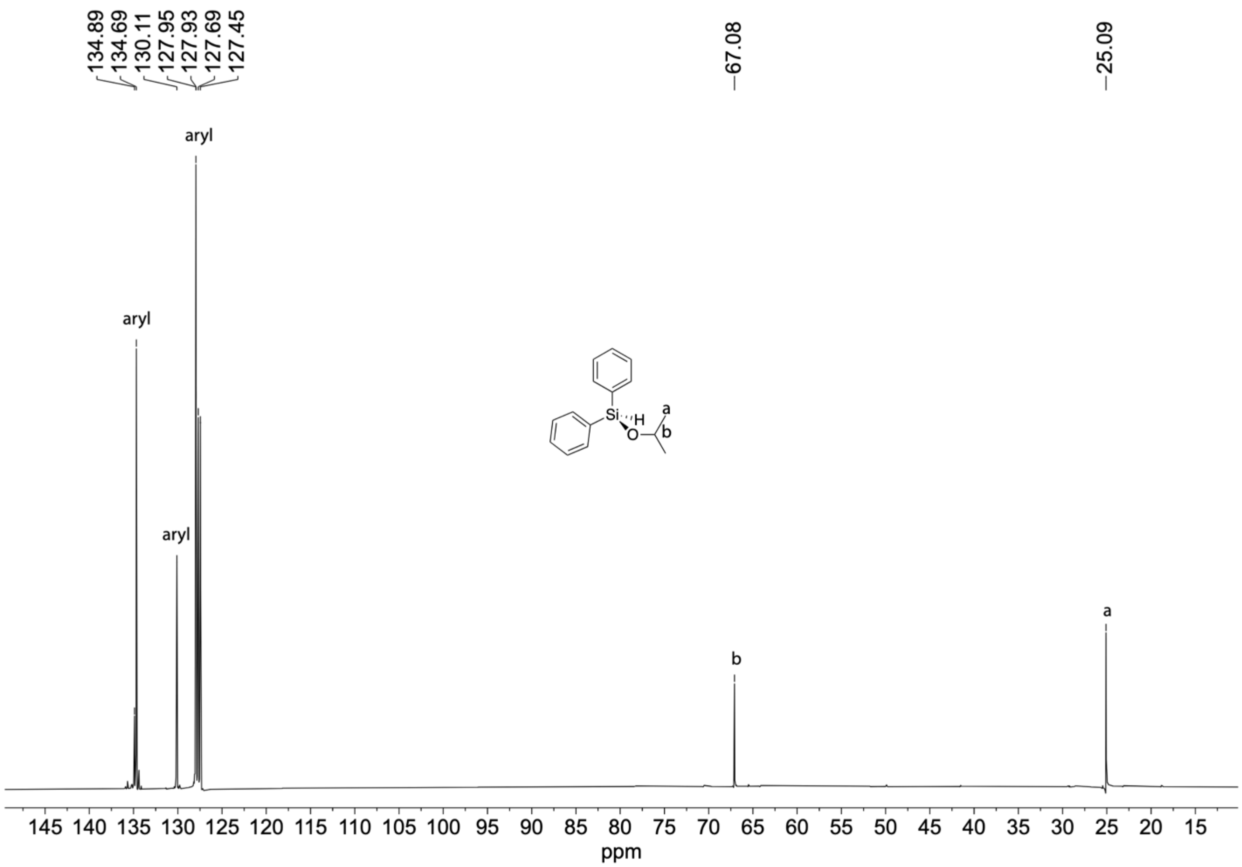

Figure S33. $^{13}C\{^1H\}$ NMR spectrum of $Ph_2HSiO\textit{i}Pr$ acquired in benzene-d_6, obtained from **$^{Ph}BOX^{Me2}ZnMe$**-catalyzed dehydrocoupling of diphenylsilane and isopropanol.

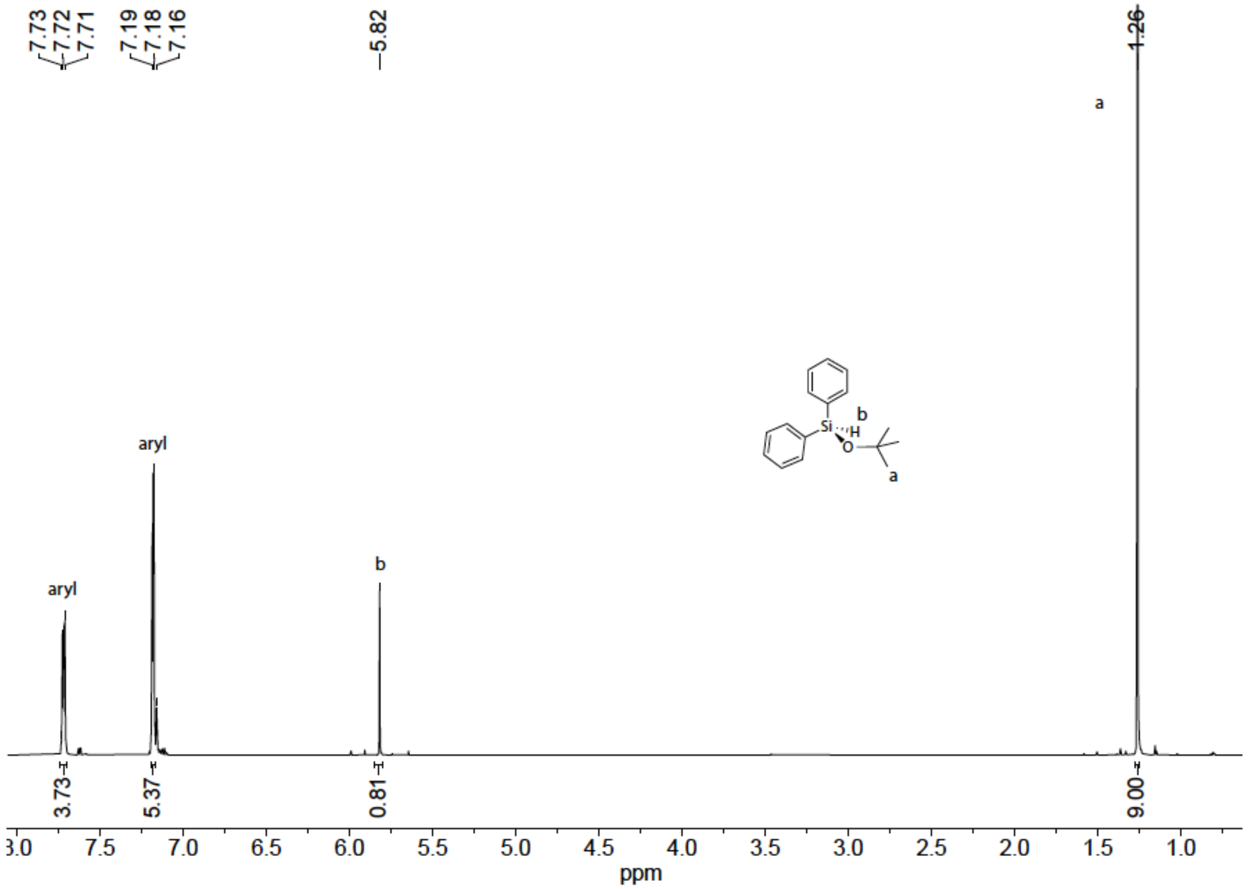

Figure S34. ^{1}H NMR spectrum of $Ph_2HSiO\textit{t}Bu$ acquired in benzene-d_6, obtained from **^{Ph}BOX^{Me2}ZnMe**-catalyzed dehydrocoupling of diphenylsilane and tert-butanol.

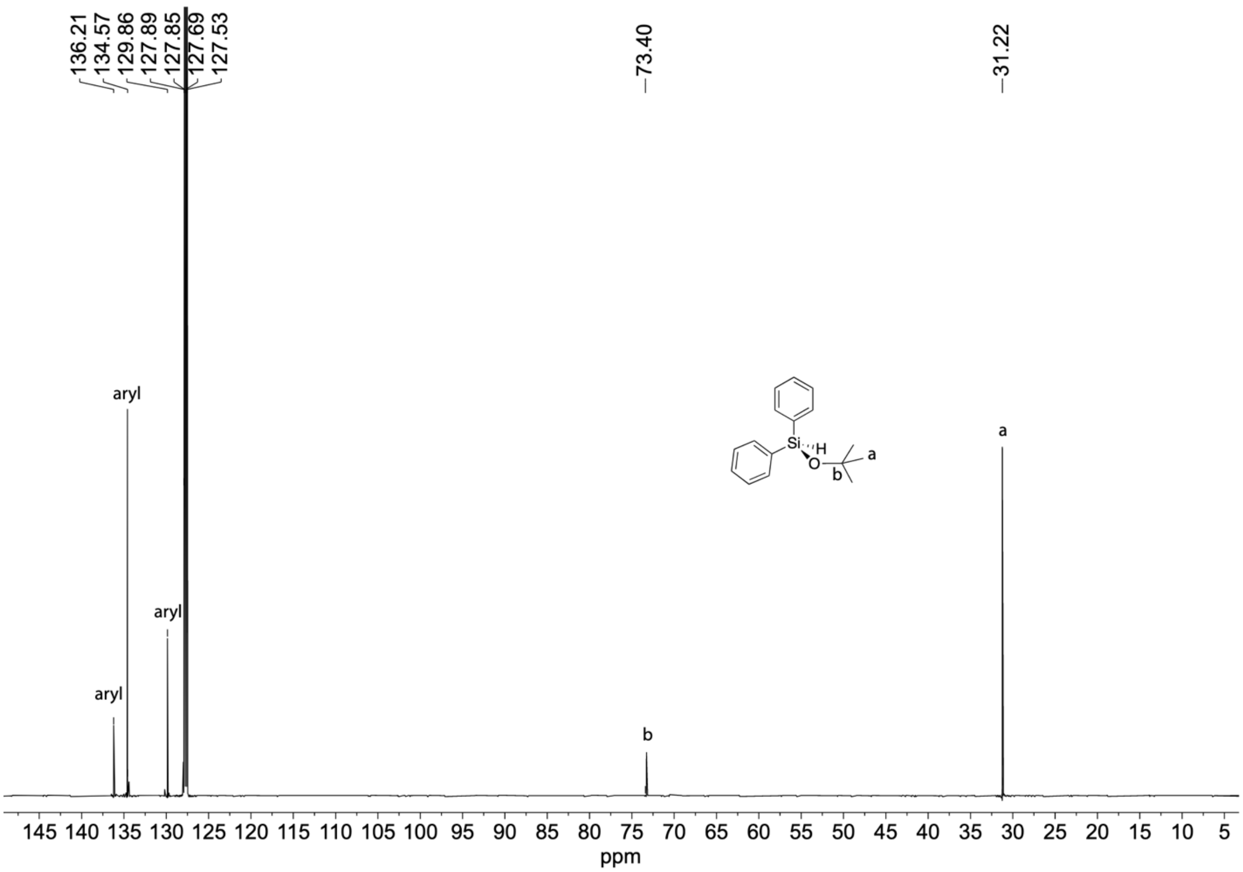

Figure S35. $^{13}C\{^{1}H\}$ NMR spectrum of $Ph_2HSiOtBu$ acquired in benzene-d_6, obtained from ^{Ph}**BOX**^{Me2}**ZnMe**-catalyzed dehydrocoupling of diphenyl silane and tert-butanol.

Procedure for Kinetic Experiments

General. Reactions were monitored by ^{1}HNMR spectroscopy using Bruker Avance-III 600 NMR spectrometer. The temperature of the NMR probe was preset to 333.2 K before each experiment and was calibrated using a thermocouple inserted through a septum into an NMR tube containing benzene. The concentration of $^{Ph}BOX^{Me2}ZnMe$ pre-catalyst was determined by integration vs. a standard of accurately known concentration of tetrakis(trimethylsilyl)silane $Si(SiMe_3)_4$ dissolved in benzene-d_6 prior to the addition of substrate. During catalytic conversions, single-scan ^{1}HNMR spectra were acquired at preset intervals (1 min or 3 min). Peak areas of signals assigned to reactants, products, and the $Si(SiMe_3)_4$ standard was used as the basis to calculate concentrations. Concentrations of $PhMeSiH_2$ or ArylOH vs time were fit via non-linear least squares regression analysis to appropriate second-order, first-order (exponential), or zero-order equations.

Representative kinetic experiment for the catalytic dehydrocoupling of $PhMeSiH_2$ and 3,5-dimethylphenol using $^{Ph}BOX^{Me2}ZnMe$. The samples were prepared by adding a measured volume (0.5 mL) of a stock benzene-d_6 solution of $Si(SiMe_3)_4$ (10.0 mM) to pre-weighed $^{Ph}BOX^{Me2}ZnMe$, giving solutions of pre-catalyst ranging from 0.61 mM to 25.1 mM. The NMR spectrometer probe was preset and calibrated to 60.1 °C. The pre-catalyst solution was placed in a septa-capped NMR tube and inserted into the NMR spectrometer to determine the concentration of the precatalyst. The NMR tube was ejected from the spectrometer. A substrate mixture (0.1 mL) containing 0.09 mL of $PhMeSiH_2$ and 0.1 g of 3,5-dimethylphenol in 1 mL of the stock benzene-d_6 solution of $Si(SiMe_3)_4$ was added to the NMR tube through the septa using microliter syringe. The hole in the septa was then covered with silicon grease to provide an additional seal. The sample was re-inserted into the pre-heated NMR spectrometer probe to commence the kinetics experiment. The concentration of $PhMeSiH_2$ and 3,5-dimethylphenol were determined by comparison of weighted integrals of appropriate resonances to the known concentration of the internal standard $Si(SiMe_3)_4$.

Moderate to high concentration of 3,5-dimethylphenol (>1.5 equiv. vs. $PhMeSiH_2$). Here we describe the procedure for kinetic measurements giving initial rates of 3,5-dimethylphenol product formation (d[P]/dt) vs [ArylOH]. Saturation behavior was tested by measuring these initial rates with increasing concentrations of 3,5-dimethylphenol. The samples were prepared by adding a measured volume (0.6 mL) of a stock solution of $Si(SiMe_3)_4$ in benzene-d_6 to $^{Ph}BOX^{Me2}ZnMe$ to

generate a 0.011 M pre-catalyst solution. The sample was transferred to an NMR tube, capped with a septum, and 3,5-dimethylphenol was added (in quantites ranging from 0.013 g to 0.183 g) by injecting through the rubber septum. The NMR tube was placed in a pre-heated NMR probe at 60 °C, and a ^{1}H NMR spectrum was acquired to determine a precise initial concentration of 3,5-dimethylphenol. The sample was removed from the probe, $PhMeSiH_2$ (0.112 ± 0.005 M) was added through the septum using a microliter syringe into NMR tube, the tube was quickly returned to the probe, and single scan spectra were acquired automatically at preset time intervals. The concentration of products at each time point was determined by integration of appropriate resonances relative and the internal standard. Linear least squares regression analysis of $PhMeHSiOC_6H_3Me_2$ concentration vs time provided rates d[P]/dt for each initial 3,5-dimethylphenol concentration. Then, the plot of d[P]/dt vs $[C_6H_3Me_2OH]_{ini}$ (Figure 5) revealed saturation, then decrease in rate as $[C_6H_3Me_2OH]_{ini}$ increased.

Moderate to high concentration of $PhMeSiH_2$. A related method, first adding excess $PhMeSiH_2$ to the solution of $^{Ph}BOX^{Me2}ZnMe$ containing $Si(SiMe_3)_4$ as an internal standard, measuring its initial concentration, then adding 3,5-dimethylphenol, and acquiring spectra at preset time intervals provided initial rates for saturation experiments in $PhMeSiH_2$. Linear least squares regression analysis of $PhMeHSiOC_6H_3Me_2$ concentration vs time provided rates d[P]/dt for each initial $PhMeSiH_2$ concentration. Then, the plot of d[P]/dt vs $[PhMeSiH_2]_{ini}$ (Figure 7) revealed saturation in rate as $[PhMeSiH_2]_{ini}$ increased.

Chart S1. Concentration ranges for Kinetic Regimes 1 and 2 and their corresponding experimental rate laws. k^{1}_{obs} rate constants referring to Kinetic Regime 1 and k^{2}_{obs} rate constants referring to Kinetic Regime 2, with primed (') experimental rate constants obtained from the slope of k_{obs} vs. [^{Ph}BOX$^{\mathrm{Me2}}$ZnMe].

A.

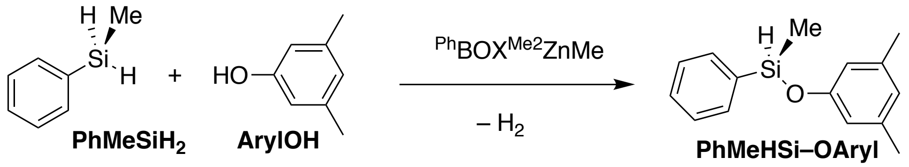

B. Rate laws and reactant concentration ratios

experimental rate laws

high [ArylOH]

Kinetic Regime 1:
[ArylOH]:[PhMeSiH$_2$] > 1.5:1

rate ~ $k^{1\mathrm{INHIB}}_{obs}$[ZnMe]^1[PhMeSiH$_2$]^1[ArylOH]$^{-1}$

rate ~ $k^{1\mathrm{SAT}'}_{obs}$[ZnMe]^1[PhMeSiH$_2$]^1[ArylOH]^0

rate ~ $k^{1'}_{obs}$[ZnMe]^1[PhMeSiH$_2$]^1[ArylOH]^1

Kinetic Regime 2:
[ArylOH]:[PhMeSiH$_2$] < 1.4:1

rate ~ $k^{2'}_{obs}$[ZnMe]^1[PhMeSiH$_2$]^1[ArylOH]^0

rate ~ $k^{2\mathrm{SAT}'}_{obs}$[ZnMe]^1[PhMeSiH$_2$]^0[ArylOH]^0

high [PhMeSiH$_2$]

C. Relationship between experimental and derived rate constants

Kinetic Regime 1:

$$k^{1'}_{obs} = \frac{k^{1A}k^{1B}}{k^{1A}[\mathrm{ArylOH}] + k^{-1A} + k^{1B}[\mathrm{PhMeSiH_2}] + K_{\mathrm{i}}[\mathrm{ArylOH}]^2}$$

$$k^{1\mathrm{SAT}'}_{obs} = k^{1B} \qquad k^{1A}[\mathrm{ArylOH}] \gg k^{-1A} + k^{1B}[\mathrm{PhMeSiH_2}] + K_{\mathrm{i}}[\mathrm{ArylOH}]^2$$

$$k^{1\mathrm{INHIB}'}_{obs} = \frac{k^{1A}k^{1B}}{K_{\mathrm{i}}[\mathrm{ArylOH}]} \qquad k^{1A}[\mathrm{ArylOH}] + k^{-1A} + k^{1B}[\mathrm{PhMeSiH_2}] \ll K_{\mathrm{i}}[\mathrm{ArylOH}]^2$$

Kinetic Regime 2:

$$k^{2'}_{obs} = \frac{k^{2A}k^{2B}}{k^{2A}[\mathrm{PhMeSiH_2}] + k^{-2A} + k^{2\mathrm{B}}} = \frac{k^{2B}}{[\mathrm{PhMeSiH_2}] + (k^{-2A} + k^{2\mathrm{B}})/k^{2\mathrm{A}}} \qquad [\mathrm{PhMeSiH_2}] \ll (k^{-2A} + k^{2\mathrm{B}})/k^{2\mathrm{A}}$$

$$k^{2\mathrm{SAT}'}_{obs} = k^{2B} \qquad [\mathrm{PhMeSiH_2}] \gg (k^{-2A} + k^{2\mathrm{B}})/k^{2\mathrm{A}}$$

Representative Kinetic Plots

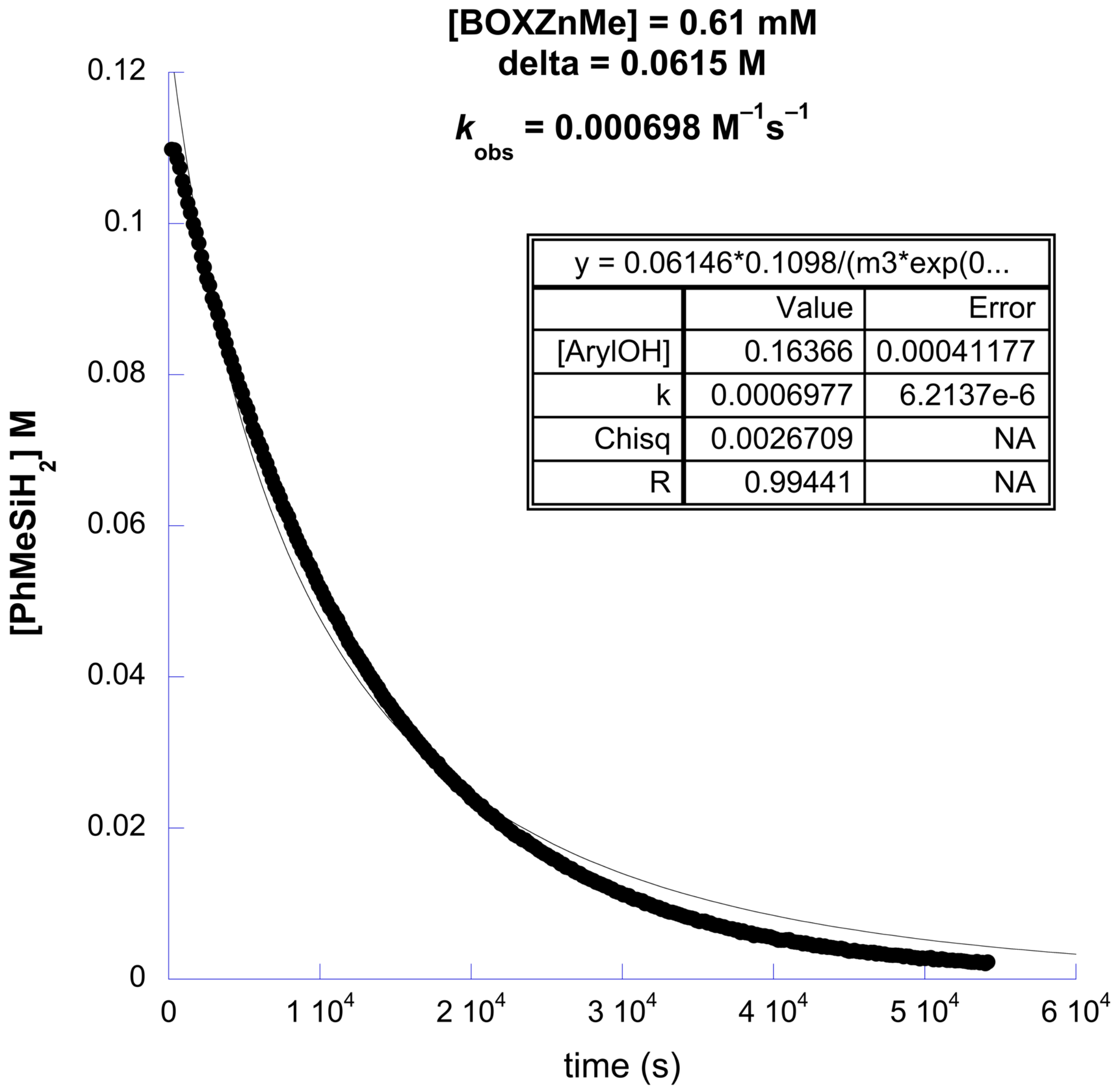

Figure S36. Representative plot of [$PhMeSiH_2$] vs time, showing second-order behavior. [$^{Ph}BOX^{Me2}ZnMe$] = 0.00061 M, [$PhMeSiH_2$]$_{ini}$ = 0.1098 M, [ArylOH]$_{ini}$ = 0.171 M, at 60.1 °C in benzene-d_6, monitored by ^{1}H NMR spectroscopy.

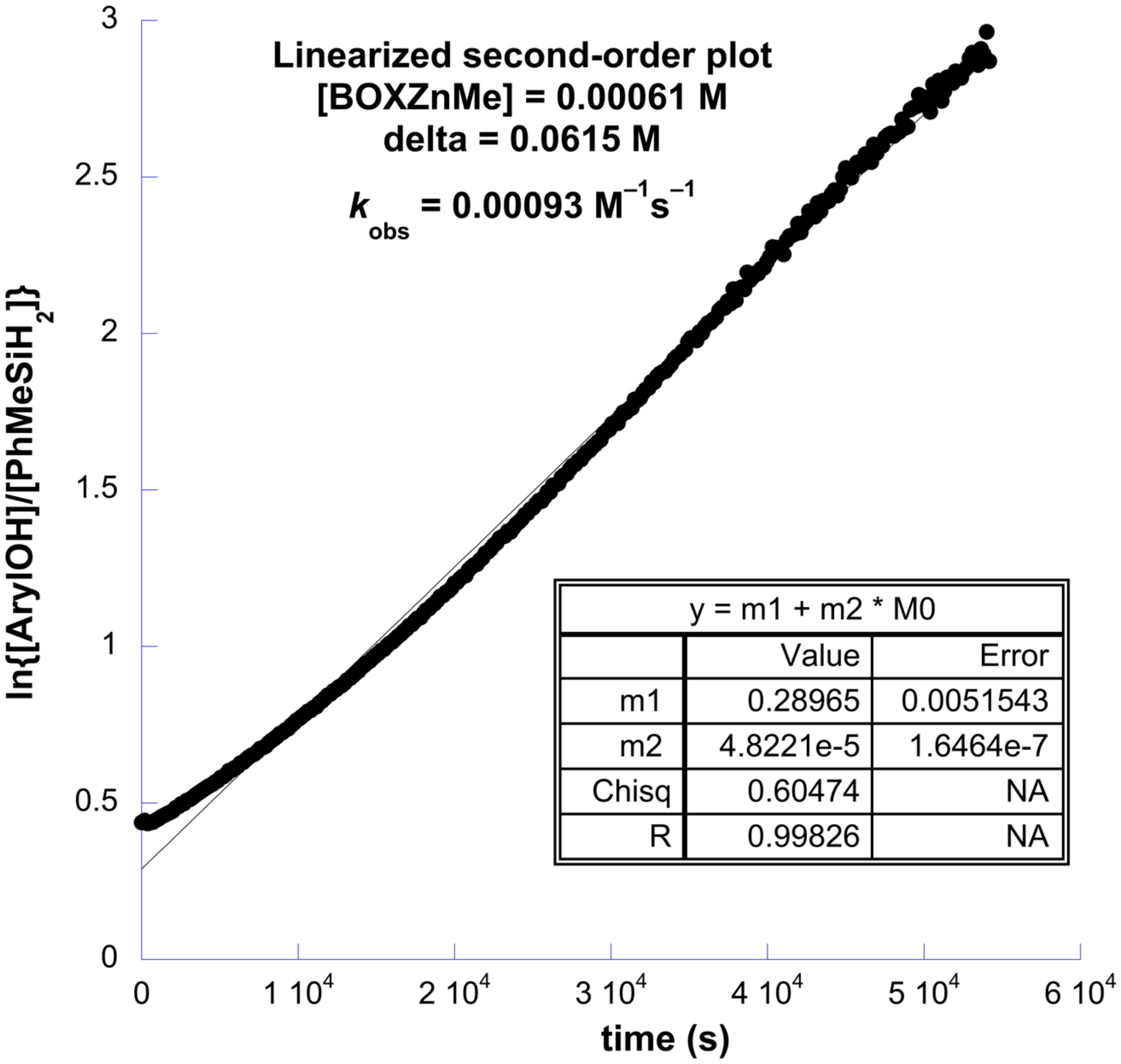

Figure S37. Plot of [$PhMeSiH_2$] vs time, showing second-order behavior. [$^{Ph}BOX^{Me2}ZnMe$] = 0.00061 M, [$PhMeSiH_2$]$_{ini}$ = 0.1098 M, [ArylOH]$_{ini}$ = 0.171 M, at 60.1 °C in benzene-d_6, monitored by ^1H NMR spectroscopy.

2.26 M

y = m1 + m2 * M0		
	Value	Error
m1	0.001762	0.00070232
m2	2.4256e-5	6.2882e-7
Chisq	8.4557e-6	NA
R	0.99732	NA

1.54 M

y = m1 + m2 * M0		
	Value	Error
m1	-0.0010152	0.00025483
m2	1.7721e-5	2.2817e-7
Chisq	1.1133e-6	NA
R	0.99934	NA

0.976 M

y = m1 + m2 * M0		
	Value	Error
m1	0.00031533	0.00061795
m2	2.2849e-5	5.5329e-7
Chisq	6.5462e-6	NA
R	0.99766	NA

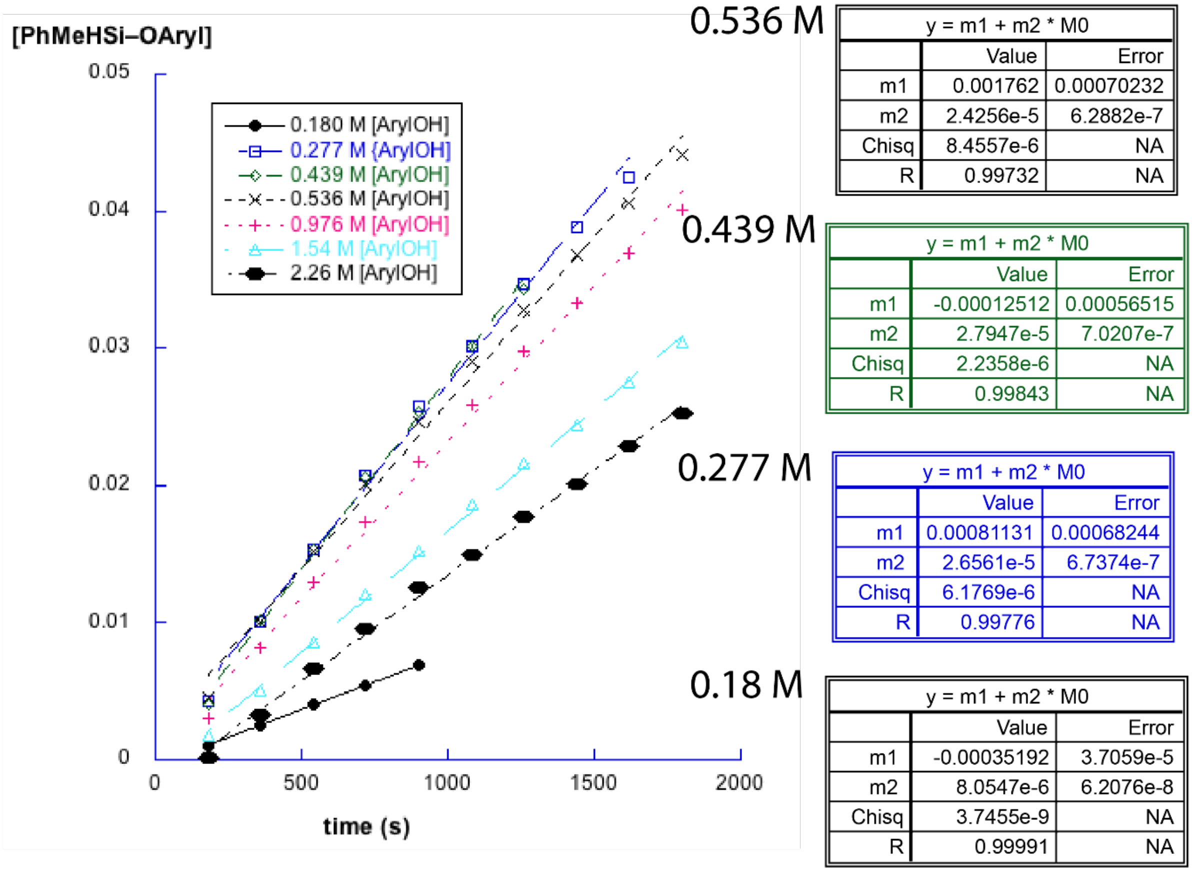

0.536 M

y = m1 + m2 * M0		
	Value	Error
m1	0.001762	0.00070232
m2	2.4256e-5	6.2882e-7
Chisq	8.4557e-6	NA
R	0.99732	NA

0.439 M

y = m1 + m2 * M0		
	Value	Error
m1	-0.00012512	0.00056515
m2	2.7947e-5	7.0207e-7
Chisq	2.2358e-6	NA
R	0.99843	NA

0.277 M

y = m1 + m2 * M0		
	Value	Error
m1	0.00081131	0.00068244
m2	2.6561e-5	6.7374e-7
Chisq	6.1769e-6	NA
R	0.99776	NA

0.18 M

y = m1 + m2 * M0		
	Value	Error
m1	-0.00035192	3.7059e-5
m2	8.0547e-6	6.2076e-8
Chisq	3.7455e-9	NA
R	0.99991	NA

Figure S38. Plot of the concentration of the product, PhMeHSiOAryl, vs time, used to determine slopes of *d*[P]/*d*t for initial rates. Data from this plot correspond to the rates plotted vs [ArylOH] in Figure 5 of the main text, describing saturation behavior of ArylOH in Regime 1.

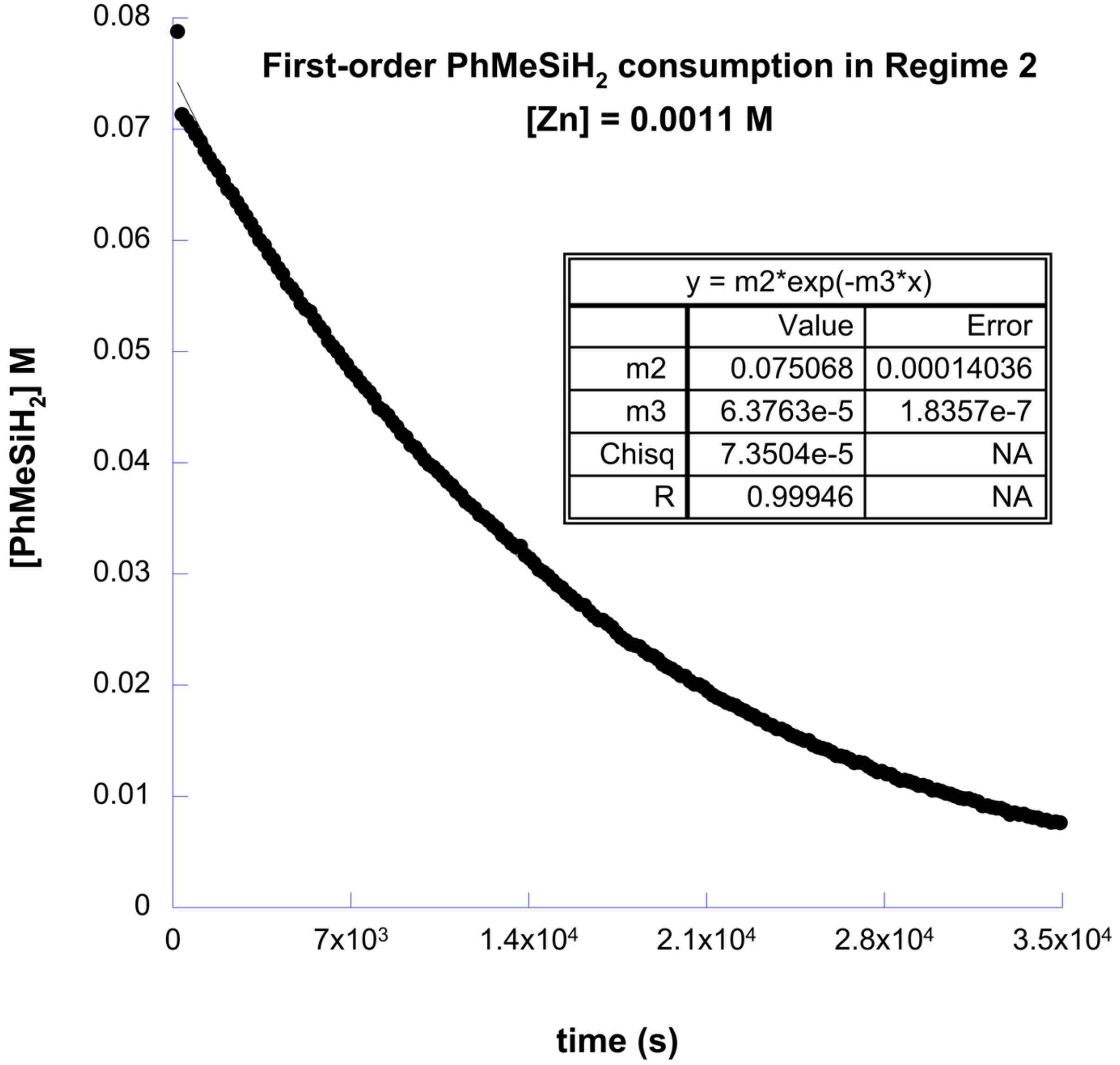

Figure S39. Plot of [$PhMeSiH_2$] vs time for ^{Ph}BOX^{Me2}ZnMe-catalyzed dehydrocoupling reaction with 3,5-dimethylphenol. [^{Ph}BOX^{Me2}ZnMe] = 0.0011 M, [ArylOH]$_{ini}$ = 0.096 M. The first-order rate constant k^2_{obs} is 6.37×10^{-5} s^{-1}, determined from non-linear least squares regression analysis.

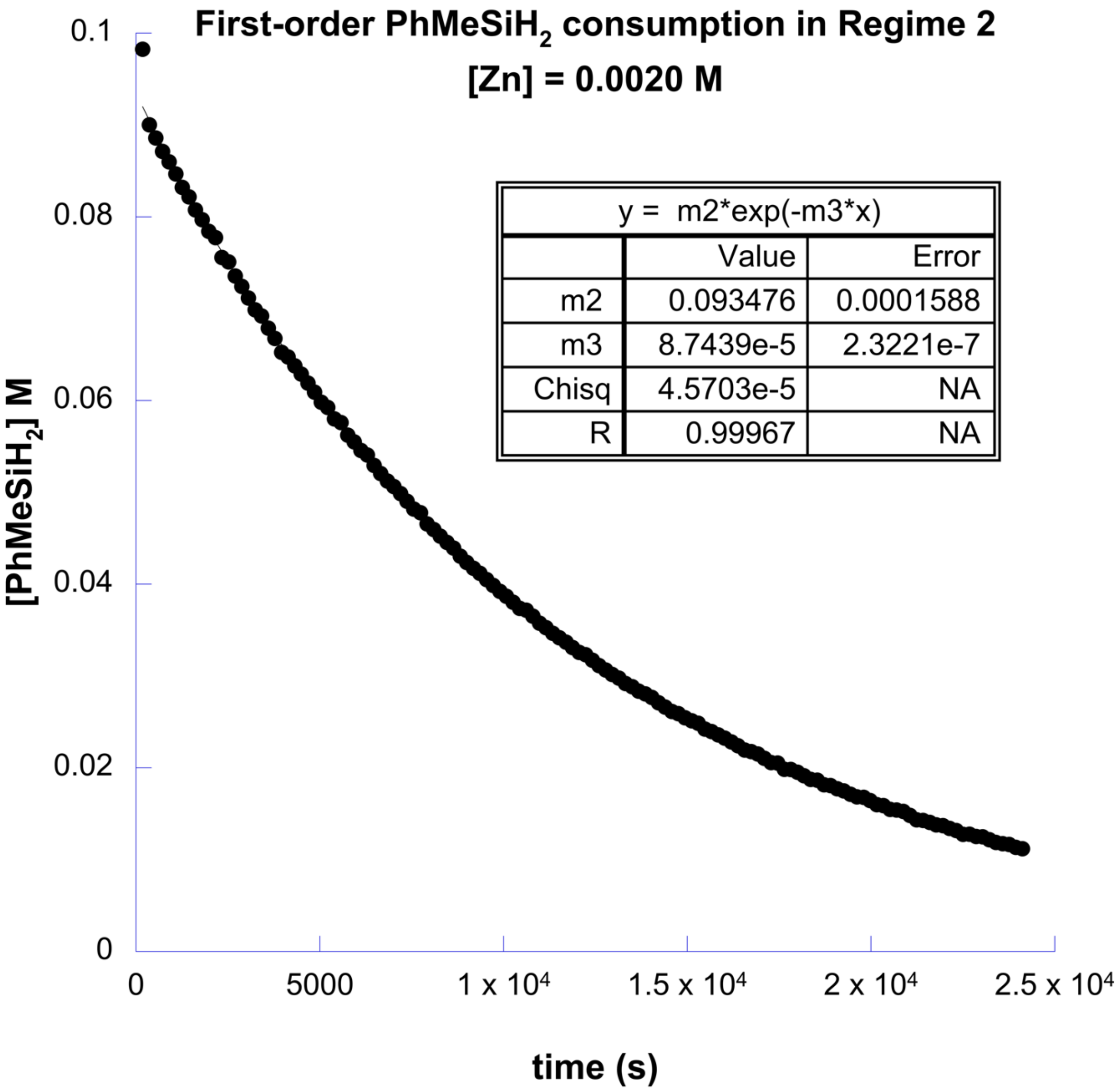

Figure S40. Plot of [$PhMeSiH_2$] vs time for $^{Ph}BOX^{Me2}$ZnMe-catalyzed dehydrocoupling reaction with 3,5-dimethylphenol. [$^{Ph}BOX^{Me2}$ZnMe] = 0.0020 M, [ArylOH]$_{ini}$ = 0.132 M. The first-order rate constant k^2_{obs} is 8.74 × 10^{-5} s^{-1}, determined from non-linear least squares regression analysis.

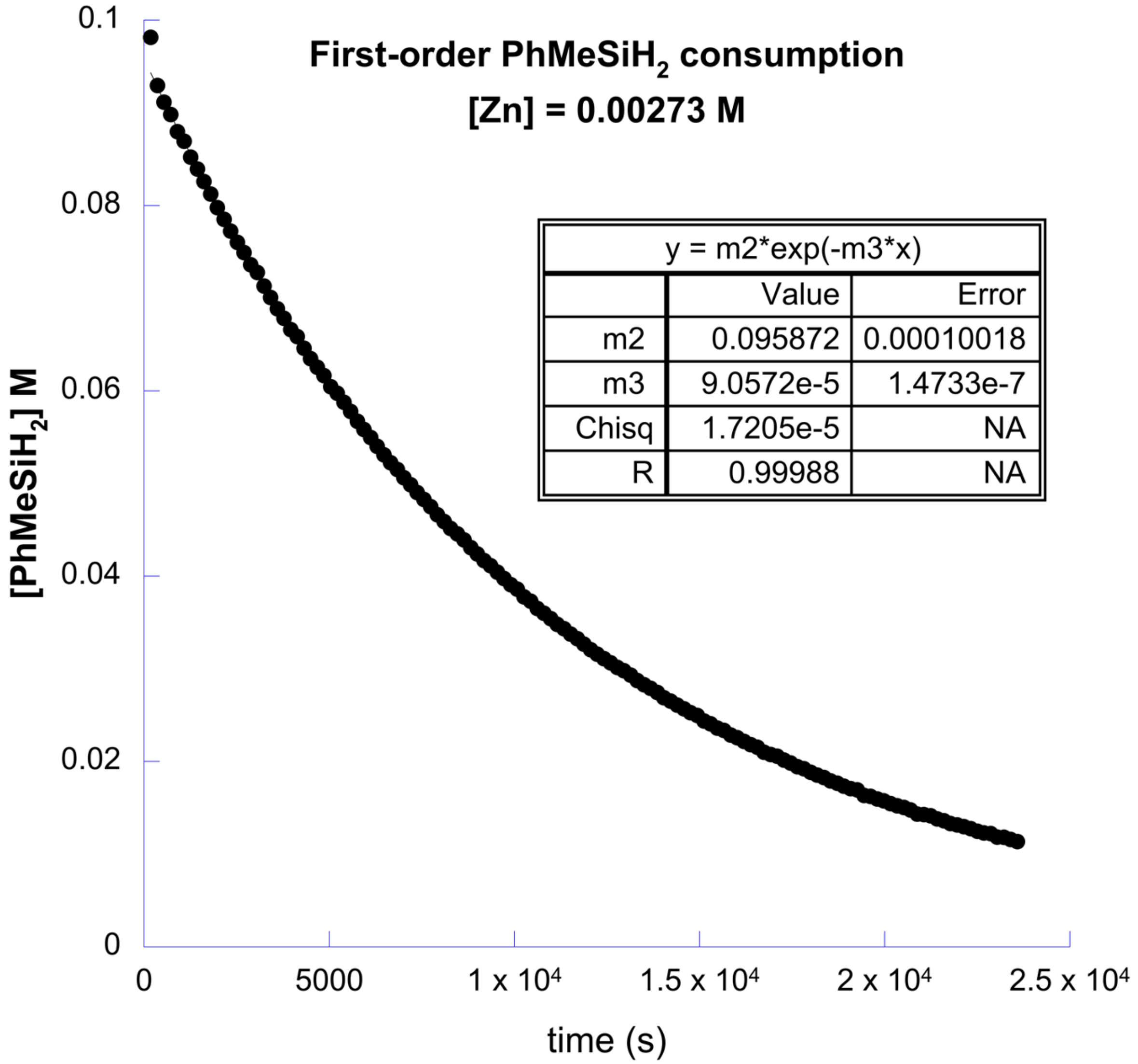

Figure S41. Plot of [$PhMeSiH_2$] vs time for $^{Ph}BOX^{Me2}$ZnMe-catalyzed dehydrocoupling reaction with 3,5-dimethylphenol. [$^{Ph}BOX^{Me2}$ZnMe] = 0.00273 M, $[ArylOH]_{ini}$ = 0.127 M. The first-order rate constant k^2_{obs} is 9.06×10^{-5} s^{-1}, determined from non-linear least squares regression analysis.

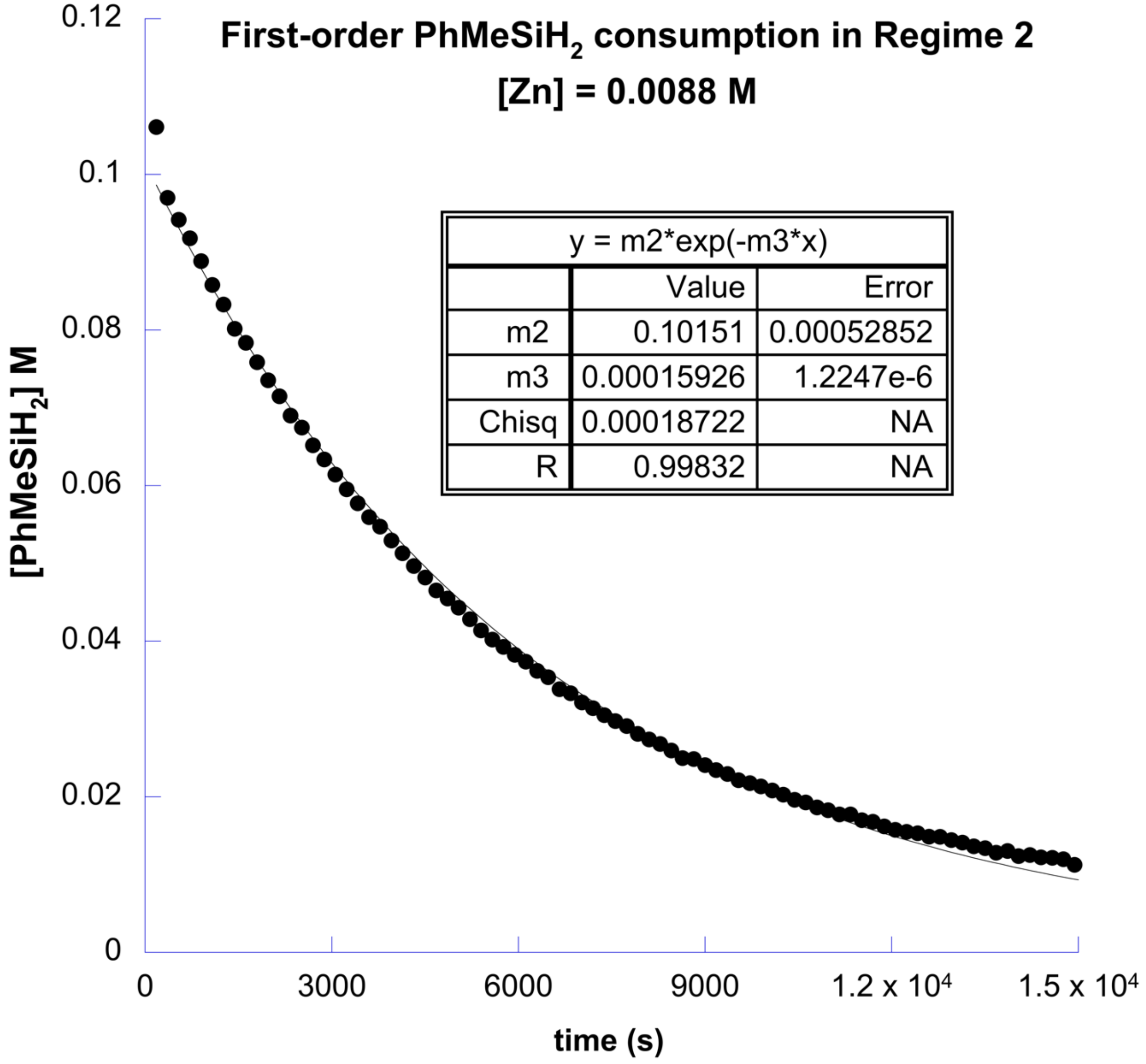

Figure S42. Plot of [$PhMeSiH_2$] vs time for $^{Ph}BOX^{Me2}ZnMe$-catalyzed dehydrocoupling reaction with 3,5-dimethylphenol. [$^{Ph}BOX^{Me2}ZnMe$] = 0.0088 M, $[ArylOH]_{ini}$ = 0.137 M. The first-order rate constant k^2_{obs} is 1.59×10^{-4} s^{-1}, determined from non-linear least squares regression analysis.

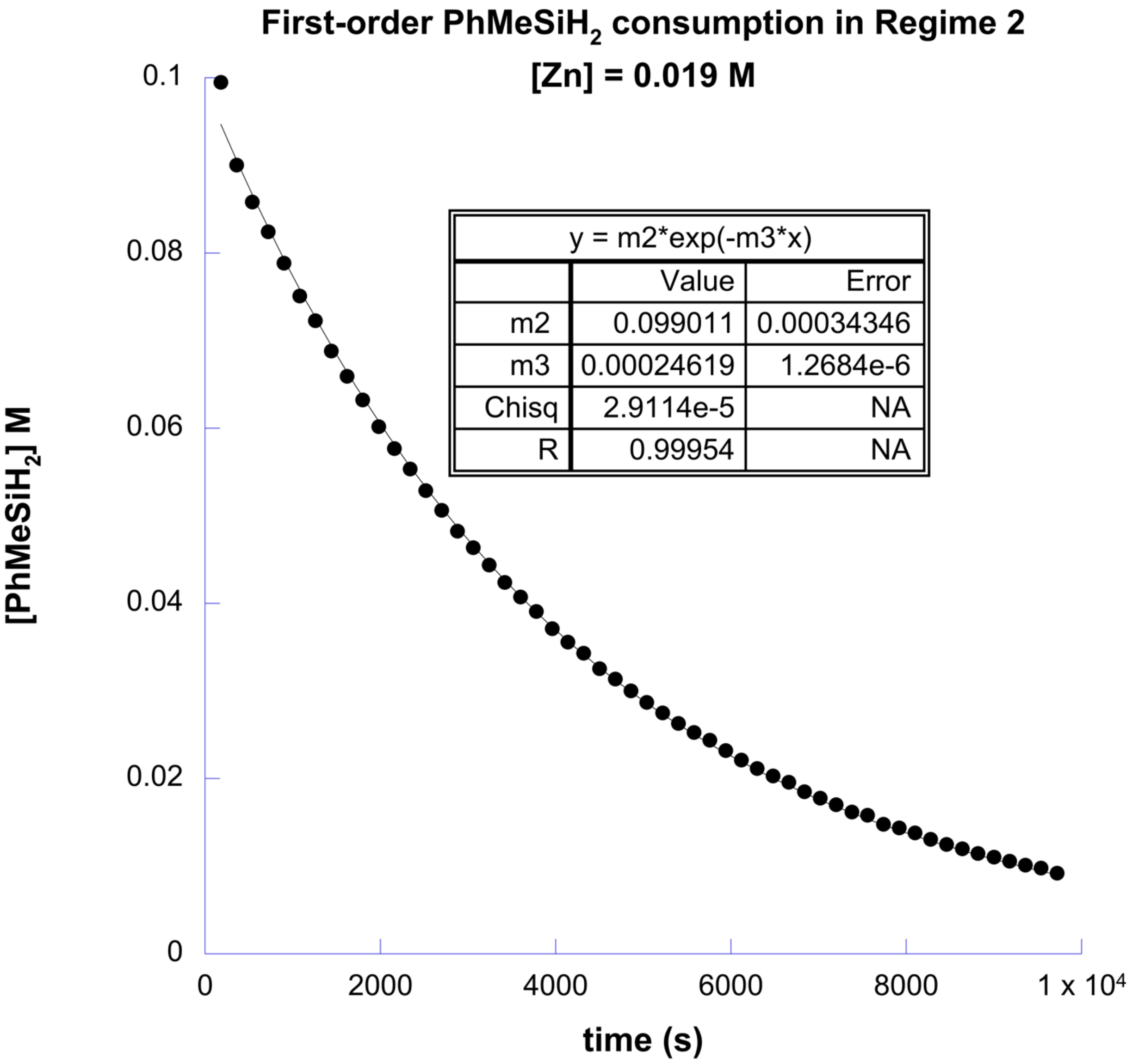

Figure S43. Plot of [$PhMeSiH_2$] vs time for $^{Ph}BOX^{Me2}ZnMe$-catalyzed dehydrocoupling reaction with 3,5-dimethylphenol. [$^{Ph}BOX^{Me2}ZnMe$] = 0.019 M, $[ArylOH]_{ini}$ = 0.128 M. The first-order rate constant k^2_{obs} is 2.46×10^{-4} s^{-1}, determined from non-linear least squares regression analysis.

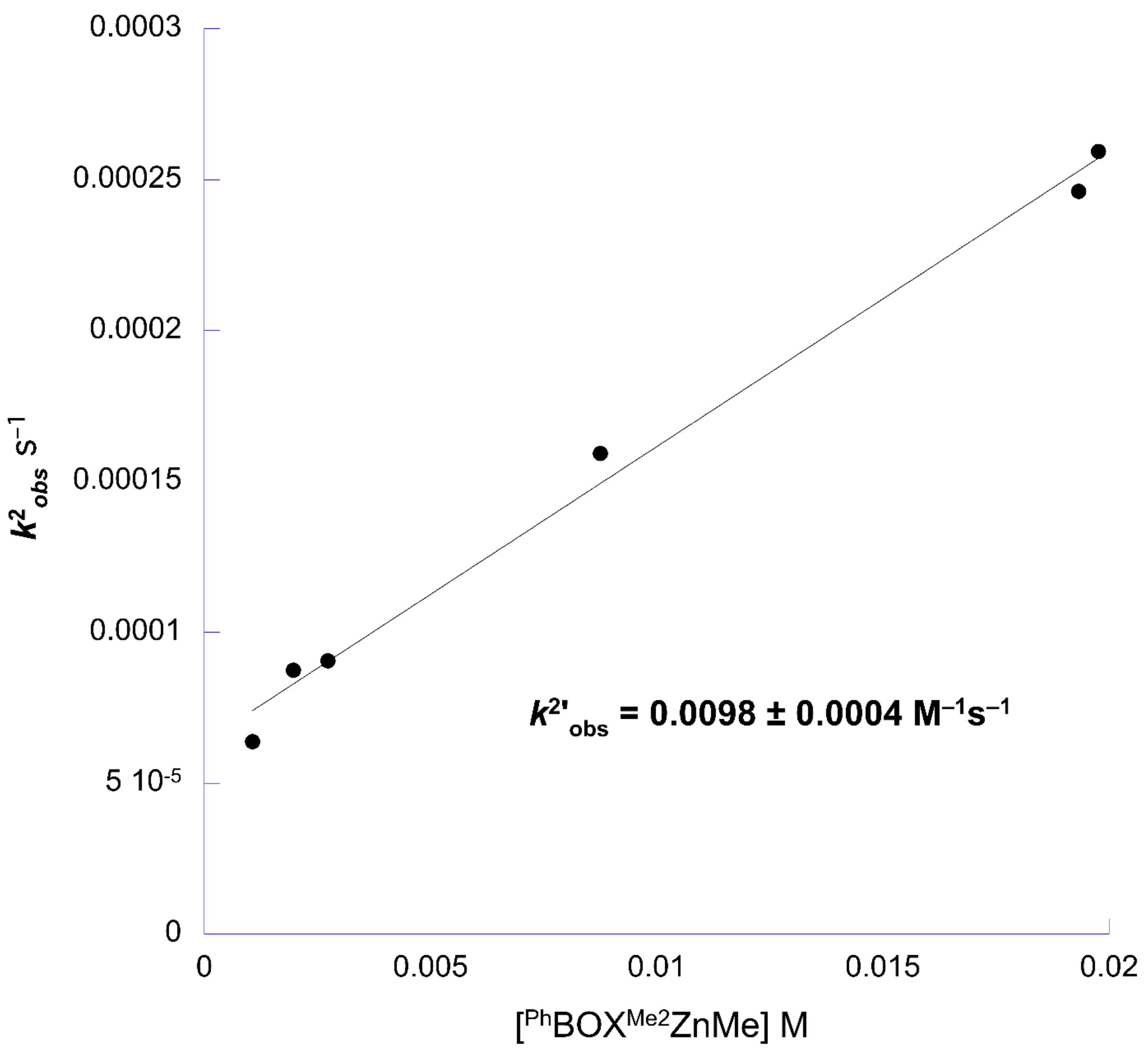

Figure S44. Plot of [^{Ph}BOX^{Me2}ZnMe] vs k^2_{obs}, showing first-order dependence on catalyst concentration (in Kinetic Regime 2). From the slope, the observed second-order rate constant $k^{2'}_{obs}$ equals $9.8 \pm 0.4 \times 10^{-3}$ M^{-1}s^{-1}.

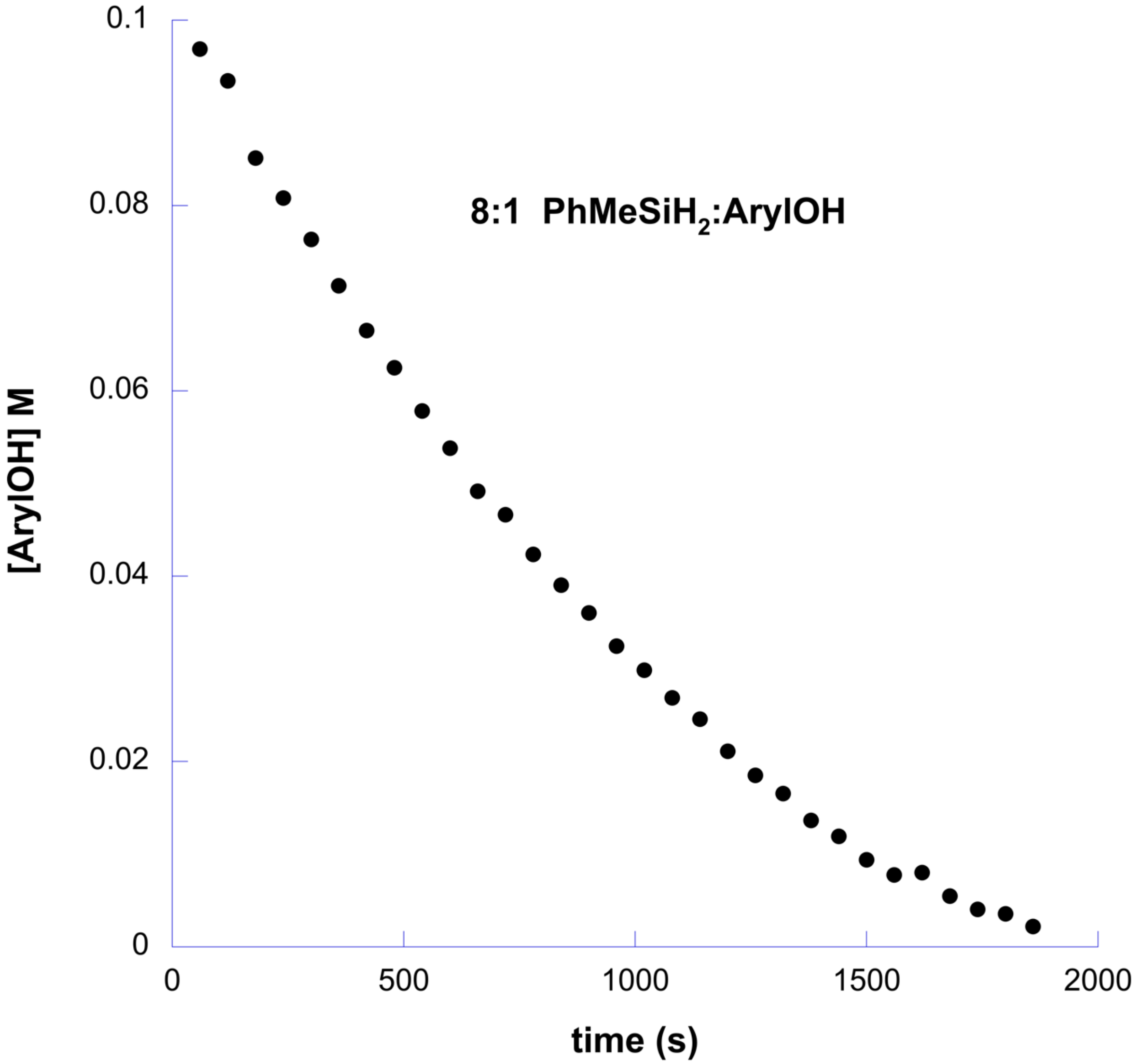

Figure S45. Plot of [ArylOH] vs. time under conditions with excess $PhMeSiH_2$ (0.85 M, 8.8 equiv. with respect to ArylOH), at a concentration range in between first-order and zero-order dependence for [$PhMeSiH_2$] (neither linear regression analysis nor non-linear regression analysis to an exponential decay gives a good fit).

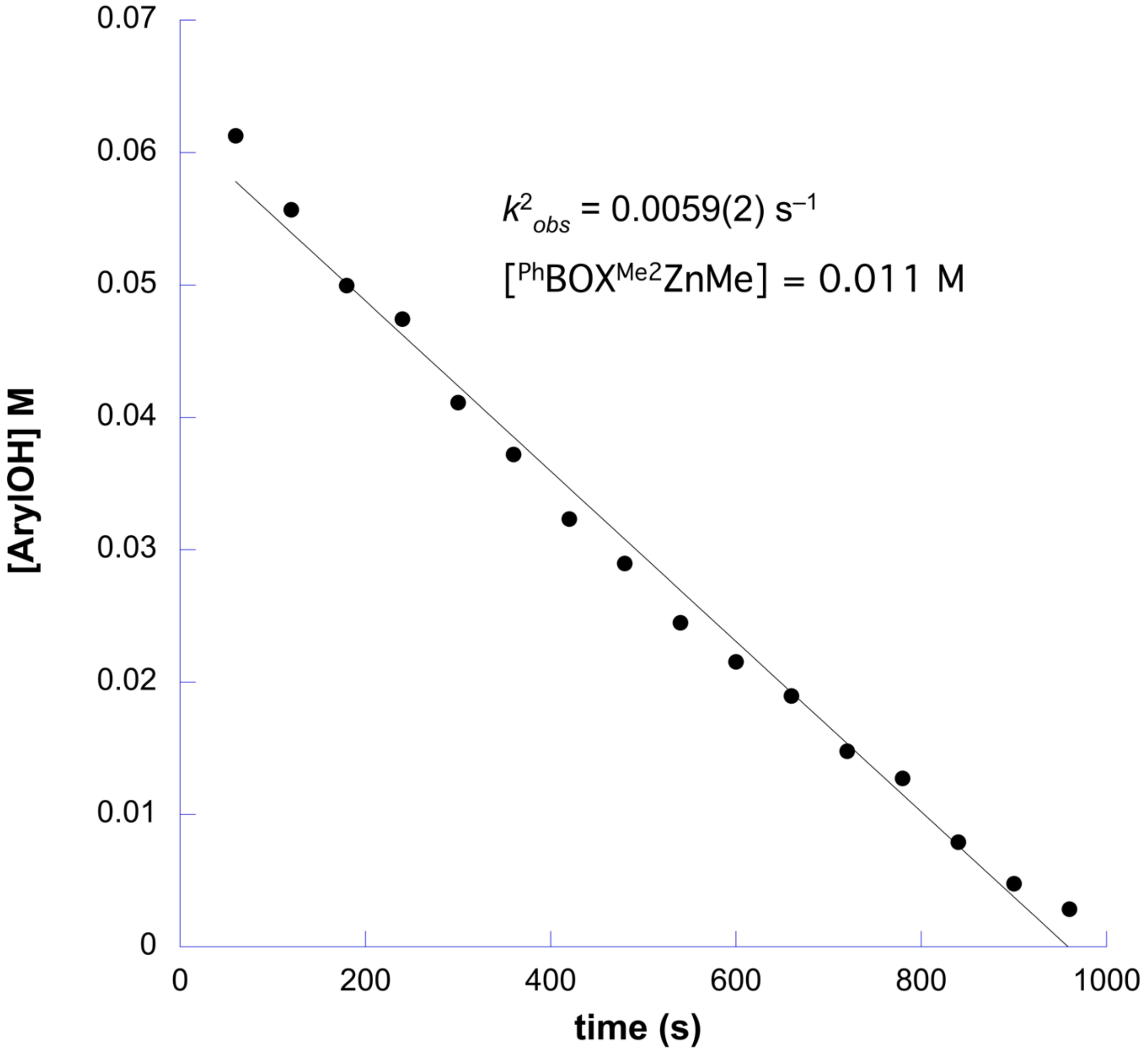

Figure S46. Plot of [ArylOH] vs. time under conditions with excess $PhMeSiH_2$ (1.26 M, 20 equiv. with respect to ArylOH), showing zero-order dependence on both [ArylOH] and [$PhMeSiH_2$]. k^2_{obs} = –slope / [^{Ph}BOX^{Me2}ZnMe]; [^{Ph}BOX^{Me2}ZnMe] = 0.011 M.

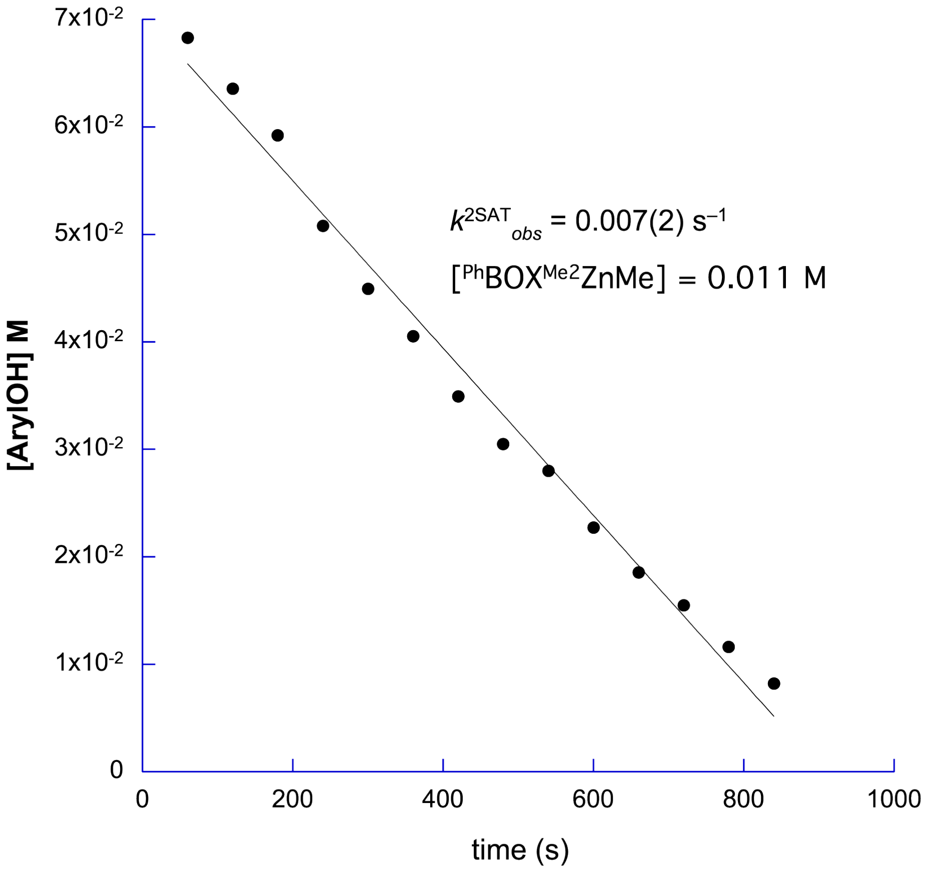

Figure S47. Plot of [ArylOH] vs. time under conditions with excess $PhMeSiH_2$ (1.88 M, 27 equiv. with respect to ArylOH), showing zero-order dependence on both [ArylOH] and [$PhMeSiH_2$]. k^{2SAT}_{obs} = –slope / [$^{Ph}BOX^{Me2}ZnMe$]; [$^{Ph}BOX^{Me2}ZnMe$] = 0.011 M.

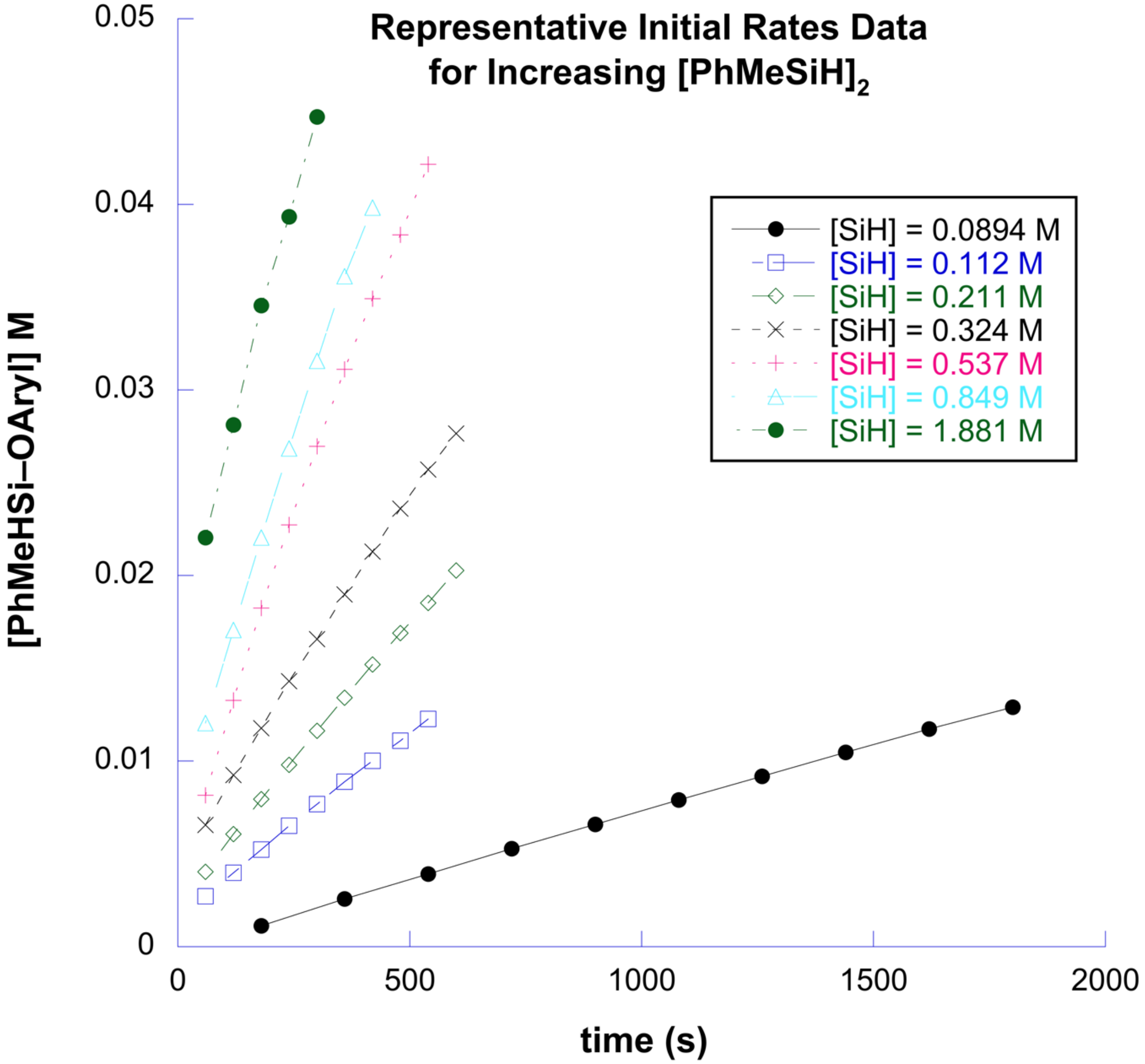

Figure S48. Representative plots of product concentration [PhMeHSi–OAryl] vs time. Variation of $[PhMeSiH_2]_{ini}$ leads to saturation in rate *d*[PhMeHSi–OAryl]/*dt*, shown in Figure 7 of the main text.

References

[1] A. Walli, S. Dechert, F. Meyer, *Eur. J. Org. Chem.* **2013**, 7044-7049.

[2] S. Lin, Y. Hou, X. Deng, H. Wang, S. Sun, X. Zhang, *RSC Adv.* **2015**, *5*, 41017-41024.

[3] M. B. Reuter, M. P. Cibuzar, J. Hammerton, R. Waterman, *Dalton Trans.* **2020**, *49*, 2972-2978.

[4] L. Mei, H. B. Yang, S. Song, *Res. Chem. Intermed.* **2010**, *36*, 181-191.

[5] D. Gasperini, A. K. King, N. T. Coles, M. F. Mahon, R. L. Webster, *ACS Catal.* **2020**, *10*, 6102-6112.
[6] S. Xu, Y. Magoon, R. R. Reinig, B. M. Schmidt, A. Ellern, A. D. Sadow, *Organometallics* **2015**, *34*, 3508-3519.
[7] M. Skrodzki, M. Zaranek, S. Witomska, P. Pawluc, *Catalysts* **2018**, *8*, 618.
[8] M. A. Esteruelas, M. Oliván, A. Vélez, *Inorg. Chem.* **2013**, *52*, 12108-12119.
[9] Y. Zhao, D. G. Truhlar, *Theo. Chem. Accounts* **2008**, *120*, 215-241.
[10] M. Valiev, E. J. Bylaska, N. Govind, K. Kowalski, T. P. Straatsma, H. J. J. Van Dam, D. Wang, J. Nieplocha, E. Apra, T. L. Windus, W. A. de Jong, *Comput. Phys. Commun.* **2010**, *181*, 1477-1489.
[11] P. J. Hay, W. R. Wadt, *J. Chem. Phys.* **1985**, *82*, 270-283.
[12] S. Grimme, *J. Chem. Phys.* **2006**, *124*, 034108.
